# Next Generation Sequence Analysis and Computational Genomics Using Graphical Pipeline Workflows

**DOI:** 10.3390/genes3030545

**Published:** 2012-08-30

**Authors:** Federica Torri, Ivo D. Dinov, Alen Zamanyan, Sam Hobel, Alex Genco, Petros Petrosyan, Andrew P. Clark, Zhizhong Liu, Paul Eggert, Jonathan Pierce, James A. Knowles, Joseph Ames, Carl Kesselman, Arthur W. Toga, Steven G. Potkin, Marquis P. Vawter, Fabio Macciardi

**Affiliations:** 1 Department of Psychiatry and Human Behavior, University of California, Irvine, CA 92617, USA; E-Mails: ftorri@uci.edu (F.T.); sgpotkin@uci.edu (S.G.P.); 2 Biomedical Informatics Research Network (BIRN), Information Sciences Institute, University of Southern California, Los Angeles, CA 90292, USA; E-Mails: ivo.dinov@loni.ucla.edu (I.D.D.); jdames@uci.edu (J.A.); carl@isi.edu (C.K.); toga@loni.ucla.edu (A.W.T.); 3 Laboratory of Neuro Imaging (LONI), University of California, Los Angeles, CA 90095, USA; E-Mails: Alen.Zamanyan@loni.ucla.edu (A.Z.); shobel87@gmail.com (S.H.); alexgenco@gmail.com (A.G.); Petros.Petrosyan@loni.ucla.edu (P.P.); zhizhong.liu@loni.ucla.edu (Z.L.); eggert@cs.ucla.edu (P.E.); jonathan.pierce@loni.ucla.edu (J.P.); 4 Zilkha Neurogenetic Institute, USC Keck School of Medicine, Los Angeles, CA 90033, USA; E-Mails: clarkap@usc.edu (A.P.C.); knowles@med.usc.edu (J.A.K.); 5 Department of Computer Science, University of California, Los Angeles, CA 90095, USA; 6 Functional Genomics Laboratory, Department of Psychiatry And Human Behavior, School of Medicine, University of California, Irvine, CA 92697, USA; E-Mail: mvawter@uci.edu

**Keywords:** Next Generation Sequencing (NGS), LONI pipeline, SNPs, CNVs, workflow, bioinformatics

## Abstract

Whole-genome and exome sequencing have already proven to be essential and powerful methods to identify genes responsible for simple Mendelian inherited disorders. These methods can be applied to complex disorders as well, and have been adopted as one of the current mainstream approaches in population genetics. These achievements have been made possible by next generation sequencing (NGS) technologies, which require substantial bioinformatics resources to analyze the dense and complex sequence data. The huge analytical burden of data from genome sequencing might be seen as a bottleneck slowing the publication of NGS papers at this time, especially in psychiatric genetics. We review the existing methods for processing NGS data, to place into context the rationale for the design of a computational resource. We describe our method, the Graphical Pipeline for Computational Genomics (GPCG), to perform the computational steps required to analyze NGS data. The GPCG implements flexible workflows for basic sequence alignment, sequence data quality control, single nucleotide polymorphism analysis, copy number variant identification, annotation, and visualization of results. These workflows cover all the analytical steps required for NGS data, from processing the raw reads to variant calling and annotation. The current version of the pipeline is freely available at http://pipeline.loni.ucla.edu. These applications of NGS analysis may gain clinical utility in the near future (e.g., identifying miRNA signatures in diseases) when the bioinformatics approach is made feasible. Taken together, the annotation tools and strategies that have been developed to retrieve information and test hypotheses about the functional role of variants present in the human genome will help to pinpoint the genetic risk factors for psychiatric disorders.

## 1. Review of the Current Methodologies and Tools for NGS DNA-Sequencing Data Analysis

The power and widespread availability of next-generation sequencing (NGS) platforms, has significantly broadened the scale of many DNA-sequencing (DNA-Seq) applications, from detecting single nucleotide polymorphisms (SNPs) [1] or copy number variations (CNVs) [2], to assembling (new) genomes or transcriptomes [3], developing quantitative RNA-sequencing (RNA-Seq) analysis [4], or detecting epigenetic changes [5]. Among many various NGS applications, we focus this review on the existing methods for processing NGS DNA-Seq data.

NGS technology allows sequencing short fragments of DNA across the whole genome, producing single end (SE) or paired end (PE) reads of 50–700 base-pairs (bp). The reads might need some pre-processing conversion step (e.g., conversion between solexa and fastq format for data produced with version of the Illumina Pipeline previous than 1.8). The resulting raw DNA-Seq read data must then be analyzed following two computational macro-processes: (1) mapping and assembling, quality control, quality score re-calibration, realignment in “difficult” regions of the genome; and (2) advanced steps focused on variant calling (SNPs, insertions-deletions (Indels) and CNVs) and annotation. These macro-processes are briefly reviewed to provide a background for the software algorithms embedded in NGS analysis. The main software involved in NGS DNA-Seq are reviewed in Table 1 and briefly described below.

**Table 1:** Review of the most used software in next-generation sequencing (NGS) data analysis. Which includes two major computational macro-processes: (1) a primary step related to mapping and assembling, with alignment quality control, quality score re-calibration, realignment in “difficult” regions of the genome; and (2) secondary, advanced steps focused on variant (single nucleotide polymorphisms (SNPs), insertions-deletions (Indels) and copy number variations (CNVs)) calling and annotation. These macro-processes are briefly reviewed to provide a background for the software algorithms embedded in DNA-Seq analysis.

Process	Software & Algorithms	Website
**Preprocessing step**	homemade script	(N/A)
**(1.1) Alignment**	MAQ	http://maq.sourceforge.net
	BWA	http://bio-bwa.sourceforge.net/bwa.shtml
	BWA-SW (SE only)	http://bio-bwa.sourceforge.net/bwa.shtml
	PERM	http://code.google.com/p/perm/
	BOWTIE	http://bowtie-bio.sourceforge.net
	SOAPv2	http://soap.genomics.org.cn
	MOSAIK	http://bioinformatics.bc.edu/marthlab/Mosaik
	NOVOALIGN	http://www.novocraft.com/
**(1.2) *De novo* Assembly**	VELVET	http://www.ebi.ac.uk/%7Ezerbino/velvet
	SOAPdenovo	http://soap.genomics.org.cn
	ABYSS	http://www.bcgsc.ca/platform/bioinfo/software/abyss
**(1.3) Basic QC**	SAMTOOLS	http://sourceforge.net/projects/SAMtools/files/
	PICARD	http://picard.sourceforge.net/command-line-overview.shtml
**(1.4) Advanced QC**	GATK	http://www.broadinstitute.org/gsa/wiki/index.php/The_Genome_Analysis_Toolkit
	PICARD	http://picard.sourceforge.net/
	SAMTOOLS	http://sourceforge.net/projects/SAMtools/files/
	IGVtools	http://www.broadinstitute.org/igv/igvtools
**(2.1a) Variant Calling and annotation**		
*Sequence Variant Analyzer v1.0, for hg18 annotations*	SVA	http://www.svaproject.org/
	SAMTOOLS	http://sourceforge.net/projects/SAMtools/files/
	ERDS	http://www.duke.edu/~mz34/erds.htm
*SAMTOOLS and ANNOVAR for annotation*	SAMTOOLS	http://sourceforge.net/projects/SAMtools/files/
	ANNOVAR	http://www.openbioinformatics.org/annovar/
*UnifiedGenotyper and ANNOVAR for annotation*	GATK	http://www.broadinstitute.org/gsa/wiki/index.php/The_Genome_Analysis_Toolkit
	ANNOVAR	http://www.openbioinformatics.org/annovar/
**(2.1b) CNVs**		
CNVseq	CNVseq	http://tiger.dbs.nus.edu.sg/cnv-seq/
	R	http://www.r-project.org/
*SAMTOOLS/ERDS/Sequence variant analyzer v1.0 ERDS*	SVA	http://www.svaproject.org/
	SAMTOOLS	http://sourceforge.net/projects/SAMtools/files/
	ERDS	http://www.duke.edu/~mz34/erds.htm
*CNVer*	CNVer	http://compbio.cs.toronto.edu/CNVer/
	BOWTIE	http://bowtie-bio.sourceforge.net
	SAVANT	http://compbio.cs.toronto.edu/savant/
**Simulated data generation tool**	dwgsim	http://sourceforge.net/projects/dnaa/

### 1.1. Alignment

The first process in DNA-Seq data analysis involves *alignment* and *assembly*. *Alignment* is the process of mapping DNA-Seq reads to a reference genome. Many sequence alignment software tools that are available today use two main algorithms: the *hash-based* and the *Burrows-Wheeler Transform* methods. 

Some hash-based algorithms build their hash table on the set of input reads (MAQ [6], Illumina’s ELAND unpublished algorithm, SHRiMP [7], ZOOM [8]). Another set of tools build their hash table on the reference genome (SOAPv2 [9], BFAST, http://genome.ucla.edu/bfast/, MOSAIK http://bioinformatics.bc.edu/marthlab/Mosaik/, Novoalign http://www.novocraft.com/main/index.php, PERM [10]). After building the hash-table these methods can either use the reference genome to scan the hash table of input reads, or use the set of input reads to scan the hash table of the reference genome.

Many recent algorithms rely on the theory of string matching using Burrows-Wheeler Transform (BWT). BWT algorithms (BOWTIE [11], BWA [12], SOAPv2 [9]) typically create a suffix array from the BWT transformed sequence, rather than from the original sequence. In the first step, the sequence order of the reference genome is modified using the BWT, a reversible process (*i.e.*, the original genome sequence can be reconstructed backwards) that reorders the genome grouping together in the data structure the sequences that appear multiple times. Next, the final index is created and is used for rapid read placement on the genome. The main advantage of BWT algorithms is their speed, as they are much faster than hash-based algorithms at the same sensitivity level [3]. 

### 1.2. Assembly

*Assembly* starts from aligned DNA-Seq reads to reconstruct the original DNA sequence computationally, which generates large, continuous regions of DNA sequence [3]. Many alignment software provide tools to perform the assembly after the read alignment (e.g., MAQ), or standalone resources can be used (SAMTOOLS [13], Emboss [14]) or commercial packages like Geneious (http://www.geneious.com) and CLC-Bio (http://www.clcbio.com). For organisms without a sequenced reference genome, it is not possible to perform any reference genome guided assembly of the reads, thus *de novo* assembly is always an essential step for data analysis. The majority of *de novo* assemblers that have been released follow two basic approaches: overlap graphs [15] and de Bruijn graphs [16]. The overlap graph method calculates all the pair-wise overlaps between the reads and report this information in a graph. The manipulation of the same overlap graph leads to a layout of reads and then to a consensus sequence of contigs using Celera Assembler [17] or Arachne [18] among others. This traditional approach is computationally intensive as the overlap graph is extremely large even for simple organisms. De Bruijn graphs algorithm is used by most assemblers (Velvet [19], SOAPdeNOVO [20], ABySS [21]) and reduces the computational charge by breaking reads into smaller sub-sequences of DNA, called k-mers, where the k parameter describes the length in bases of these sequences [22]. The *de novo* assembly can be used also to resolve complex genomic region (e.g., rapidly evolving or rich in repetitive elements) of organisms with a reference genome. In this case the contigs are aligned back to the reference genome and can undergo all the next analytical steps here described.

### 1.3. Quality Control Improvement of Reads

There are many issues that must be considered when dealing with NGS data, beginning with the alignment of short reads. As an example, since each read is aligned independently, many reads spanning Indels may be misaligned. The per-base quality scores (*i.e.*, the probability that the called base in the read is the true base [23]) may also be inaccurate due to systematic errors in sequencing technology, machine cycle, and sequence context [24,25,26]. Thus, following the alignment and/or assembly of reads, quality control steps are implemented before continuing in the downstream analyses (in Section 2.1).

#### 1.3.1. Basic Quality Control and File Formatting

A first basic quality control (QC) check involves formatting the aligned reads in a conventional format (e.g., Sequence Alignment/Map (SAM) or Binary Sequence Alignment/Map (BAM)). The output of this process is a clean, sorted, indexed file in BAM format that can be subjected to more advanced QC procedures, or be used directly in the downstream analyses. 

#### 1.3.2. Advanced QC

Additional advanced QC steps are strongly recommended since misaligned reads and inaccurate quality scores affect the reliability of the subsequent SNP discovery and genotyping steps, without correcting for such stochastic and systemic errors, the rate of false positive calls can be really high [27]. Even though we lack a gold standard for these procedures, there are computational tools to perform advanced QC on the data, in addition to some basic descriptive statistics and quality metrics visualization (SAMTOOLS [13], PICARD, http://picard.sourceforge.net/, Genome Analysis Toolkit (GATK) [27]). GATK supports locally realigning reads across regions enriched in Indels, recalibrating base quality scores of sequencing reads to correct for variation in quality with machine cycle and sequence context. This advanced QC of NGS data is probably the most important process to guarantee an accurate variant call, which is the immediate downstream analytical step.

### 1.4. Variant Calling and Annotation

Once the reads have been aligned and calibrated, SNPs, Indels and CNVs can be called. This step requires sensitive and specific statistical models and tools [6,9,12,13,28,29,30,31,32,33,34,35,36,37,38,39,40,41,42,43,44,45,46,47,48], named in Table 1.

#### 1.4.1. SNPs and Indels Calling and Annotation

There are many algorithms that may be used to call SNPs from NGS data (SAMTOOLS [13], GATK, MAQ, SOAPv2, UnifiedGenotyperV2 within the GATK suite) and some recommended analytical and statistical frameworks [49], even if a gold standard for variant calling is still lacking as statistical methods for analyzing the data are constantly being released [49]. The SNPs and Indels are exported from these tools in variant call format (VCF), with much information related to each variant (*i.e.*, quality score, coverage, estimated genotype). Once the variants have been called they need to be annotated, and this is a step that, until recently, only a few computational tools were able to accomplish like ANNOVAR [50], Sequence Variant Analyzer (SVA) [51], and GATK [27].

#### 1.4.2. CNVs Calling

Furthermore, the field of computational methods for discovering structural variation on NGS data is still an open computational and bioinformatics challenge [2]. The CNVs discovery methods operate following a framework that allows detecting anomalous “signatures” or patterns, then calls the related variants using mainly four different approaches [2]: (1) read pair methods; (2) read-depth methods; (3) split read approaches; (4) *de novo* assembly. 

### 1.5. Statistical and Variant Prioritization Analysis

Additional software such as PLINKseq (http://atgu.mgh.harvard.edu/plinkseq/) implement statistical models to analyze variants called from NGS experiments, testing for association with continuous or dichotomous traits and assessing an unusual distribution for rare variation across different functional categories [52]. Some other tools like PolyPhen2 [53] and VAAST [54] can be used afterwards for functional variant annotation and prioritization providing hints on the biology and pathophysiology of psychiatric disorders. Also, alternative annotation tools and strategies have been proposed [55] to retrieve information and test hypotheses about the functional role of variants present by chance in any single human genome or enriched in the genome of people affected by a psychiatric disease [56,57].

### 1.6. Graphical Workflows

The development and release of algorithms and software for analysis of NGS data has seen exponential growth in the last two years, requiring a huge investment in terms of time, expertise and computer infrastructure. 

From this brief review of tools (Table 1), it is evident that analyzing NGS data is a challenging and time consuming operation for scientists. Ideally, these tools must be up to date and easy to use, and their sequential combination should optimize performance and accuracy, with each program producing output files compatible with the input requirements of the software performing the following operation. Such processes sequentially linked together build what is generally called a workflow. An increasingly large number of workflows are available today to manage high-throughput genomics sequencing data, from basic data processing to high-quality visualization of results. Examples include shell-scripts [58,59], tool-specific graphical interfaces [60,61], and graphical workflow environments [62,63,64,65]. The graphical workflow environment are emerging as useful for constructing, modifying, interconnecting and executing computational genomics protocols using data processing workflows, also described as “pipelines” once the processes have been connected (Table 2). 

**Table 2:** Comparison of several Graphical Workflow Environments to manage pipelines. Most workflow environments provide graphical solutions (infrastructures) for the interactive handling of data, with several advantageous features compared to the management of the same processes via command line or scripting interfaces. When adding new software tools, some of these architectures require software recompilation and some do not. Yet, there is significant variation of the status reports generated during or after workflow execution. Data storage, internal or external, operating system and local hardware dependencies and utilization of available grid managers also vary between the different workflow environments. There are many synergies between the Pipeline and various alternative environments for software tool integration and interoperability, with also some valuable differences. The Laboratory of Neuro Imaging (LONI) pipeline infrastructure provides computational workflow execution capability with or without the use of local hardware or administrative support. Adding new software tools to the pipeline library of tools is efficient, does not require recompiling the programs, and requires only a brief description of the tool invocation syntax using the client “module description” dialog. Thus, the LONI pipeline offers a flexibility and simplicity in design of novel workflow solutions that is not available in the other two most used systems for NGS data analysis, Taverna and Galaxy. Similarly, the LONI pipeline allows workflow pausing and resuming, and provides explicit controls ensuring that processes are only instantiated when the complete upstream activities have successfully completed execution. Additionally, the available Taverna and Galaxy services have restrictive upper limits on storage (100 GB) and per-process RAM (64 GB), when they are deployed on Amazon Web-Services/Cloud creating bottlenecks with data staging to/from the servers and computational runs. The Pipeline service provides a pair of dedicated open-access servers (http://genomics.loni.ucla.edu) each with 40-cores and 1.4 TB of shared RAM.

Workflow Management System	Module concatenation and interoperability	Asynchronous Task Management	Requires Tool Recompiling	Data Storage	Platform Independent	Client-Server Model	Grid Enabled
LONI Pipeline [57]	Y	Y	N	External	Y	Y	Y
**pipeline.loni.ucla.edu**							
Taverna [61]	Y	N	Y	Internal(MIR)	Y	N	Y
**taverna.sourceforge.net**							
Kepler [54]	Y	N	Y	Internal(actors)	Y	N	Y
**kepler-project.org**							
Triana [66]	Y	N	Y	Internal data structure	Y	N	Y
**trianacode.org**							
Workflow Navigation System [67]	N	N	N/A	External	Y	N	N
**wns.nig.ac.jp**							
Galaxy [55]	N	N	Y	External	N	Y	N
**usegalaxy.org**							
VisTrails [55]	Y	N	Y	Internal	N	N	N
www.vistrails.org							

*Workflow Management System*: list of the compared graphical workflow environments. *Module concatenation and interoperability: Asynchronous Task Management*: ability to submit new workflows and report the status of executing or completed workflows asynchronously, e.g., constant interruptions of network connectivity. *Requires Tool Recompiling*: requirement to recompile new computational libraries or software tools against the graphical environment libraries, and to restart the environment when provisioning these new services. *Data Storage*: ability of environment to store data (raw, processed and derived) internally (RAM/DB) or externally (NFS/Services). *Platform Independent*: dependency of the environment on the local hardware and operating system. *Platform independence refers to the workflow environment itself, not the computational library of tools that are accessible via that environment. For environments with Client-Server architecture, this is irrelevant, as the platform-independent clients can always connect to, submit data, process protocols and monitor the status of executing pipeline workflows by connecting to (possibly platform dependent) back-end pipeline servers where specific operating systems (most commonly Linux) may be required by many informatics and genomics computing libraries. Client-Server Model*: independent server and clients that can be broadly interconnected provided. *Grid Enabled*: use of a Grid Engine/Grid Job Manager. Legend: Y = yes, N = no.

There are several additional features in graphical workflow environments that simplify the data management. Table 2 lists some of the commonly used workflow environments and compares their core features. Each of the graphical environments described in Table 2 allows design and submission of new workflows. 

The most commonly used systems in NGS analysis are Taverna [68] and Galaxy [64]. The latest beta-version of the Galaxy (http://galaxy.psu.edu/) platform offers a NGS computational framework that embeds single processing units to be invoked as web-services but it still lacks the functionality of interlinking the outputs of one process into a subsequent module. The available Taverna and Galaxy services have restrictive upper limits on storage and per-process RAM, when they are deployed on web-services, creating bottlenecks with data staging to/from the servers and computational runs. Also, the library of available Galaxy routines doesn’t allow adding new tools and is limited to a few alignment software tools (e.g., BWA and Bowtie), or on the quality control side, an incomplete beta-version of the PICARD suite. 

The LONI (Laboratory of Neuro Imaging) pipeline displays unique features for the implementation of new interactive and robust NGS analysis workflow protocols using a graphical environment. The design of novel workflow solutions within the LONI pipeline environment is simple and flexible [69], and allows embedding and connecting heterogeneous software within the same computational protocol without the need of advanced bioinformatics skills (Table 2).

The LONI pipeline architecture [70,71] is a distributed environment utilizing a client-server interface for design, validation, execution and dissemination of computational protocols as graphical workflows. Individual applications are represented as modules and may be linked to form complex network implementation of the desired analytical processes. Using a flexible, user friendly and customizable data processing and visualization system, the LONI pipeline environment provides access to distributed datasets, heterogeneous software tools and diverse web-services. Additional details about the LONI pipeline environment are available in [65].

Based upon the review of the NGS software (Table 1) and the graphical workflow environments to manage workflow (Table 2), we chose to develop within the LONI environment a graphical environment for genomics, called the Graphical Pipeline for Computational Genomics (GPCG) that covers many informatics analytical steps on NGS data. This effort was a joint collaboration between LONI at UCLA, BIRN (Biomedical Informatics Research Network) at UCI, Information Sciences Institute (ISI) at USC.

The GPCG is a set of workflows that may simplify and speed-up approaches for sequencing projects performed on Illumina/Solexa Genome Analyzer-HiSeq platform [72,73,74]. We have implemented workflows in GPCG that: (1.1) aligns reads (both in single and paired end) and (1.2) performs *de novo* assembly with multiple algorithms; (1.3a) performs basic formatting and quality control, followed by a (1.3b) more advanced and complex quality control to correct for sequencing biases; (2.1a) performs SNP-Indels calling and annotation, and (2.1b) CNVs calling. The Supplementary Materials S1 provides detailed information about our experiments and results. 

## 2. Description of the GPCG

**Figure 1:**
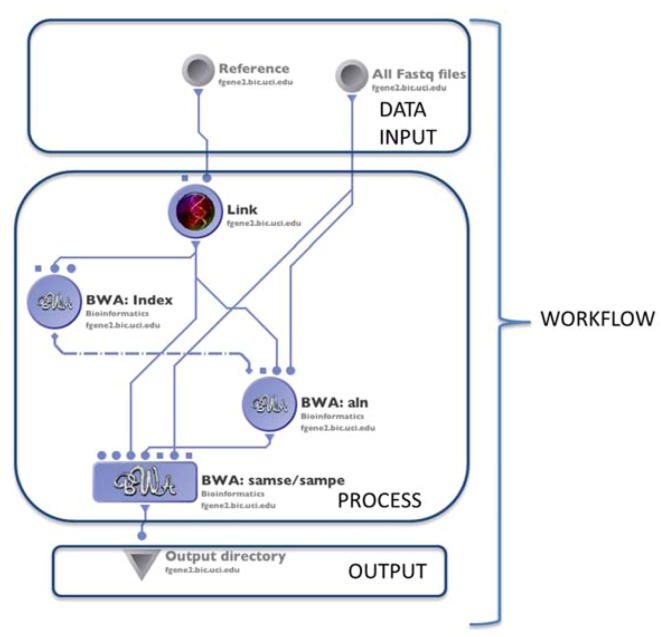
A snapshot of the general organization of a workflow within the LONI pipeline environment. This is an example of embedded modules into an alignment workflow based on BWA software. The user can simply set up the location of the input files in the data sources, manage the programs involved in the core modules, and indicate the location of the output files in the output data sink section. Every section can be interactively edited or modified through a menu of options accessed by right-clicking the mouse on the respective portion of the workflow.

The GPCG is a collection of “ready to use” workflows covering a broad spectrum of DNA-Seq data analysis steps. We have converted each single “command line” process into a module definition, and then connected those modules involved in the same process logically to form a workflow (Figure 1). We have generated a comprehensive Supplementary Materials Chapter S2 that includes all details to deploy the GPCG infrastructure, reproduce the workflow designs, and validate the results reported in this paper. The full description of all the workflows is reported in Supplementary Materials S1. In the data source section user can specify the location of input data, a process section where modules can be linked to perform complex analysis steps, and an output section where the name and location of the output files can be specified (Figure 1).

**Figure 2:**
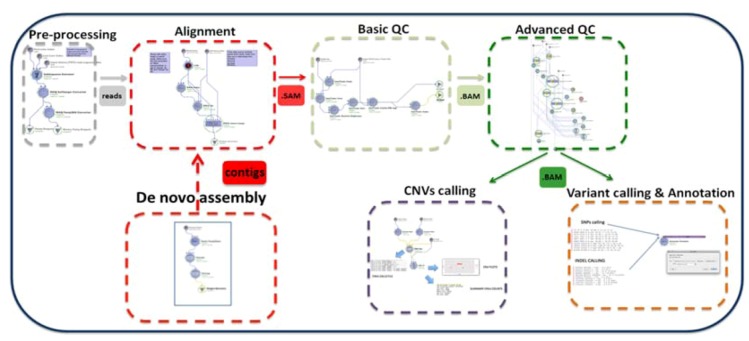
An example of the workflow approach to analyze DNA-Seq data in GPCG. Several alternative workflows can be run independently or connected in a logical flow. Once the reads have been pre-processed, they can be aligned (1.1), undergo (1.3) Basic and (1.4) Advanced QC, (2.1a) SNP/Indels and (2.1b) CNVs calling and annotation. The reads can also undergo (1.2) *de novo* assembly, and if a reference genome is available the contigs can be realigned back to the reference genome and then undergo the following computational processes.

A single workflow (Figure 1) can be run independently from others, or can be connected as illustrated by the analytical workflow protocols (Figure 2 and Table 3). For example, the user can reconnect workflows by dragging and dropping within the GPCG creating a new pipeline. Modularity, reuse and interconnectivity are key features within the LONI environment, which make the system flexible for the analytical needs of the researcher.

The current implementation of DNA-Seq workflows is summarized in Table 3. Together with its flexible connections, the GPCG incorporates alternative algorithms for the vast majority of the processes. The choice of the algorithm is critical, as different approaches might produce different results. 

Users may choose in fact the most suitable analytical model for their data or develop additional module descriptions interfacing other computational tools. Additional tools, workflows and updates are available on the LONI pipeline Navigator website: http://pipeline.loni.ucla.edu/services/library-navigator. As a large number of different file formats are involved in these workflows, we provide a glossary and examples of all the formats encountered in Supplementary Table S1.

We have implemented a preprocessing module that allows extracting a subset of reads to perform a validation and initial testing of pipeline modules before running an entire dataset. Embedded within this module are routines to convert read quality scores from Solexa FASTQ files to the Sanger scale (for data produced with Illumina pipeline versions previous than 1.8), and to binary FASTQ as requested by some aligners like MAQ [40,75]. Once the read subset is ready, it is possible to proceed to the two main computational processes previously described. We also embedded another module in the pipeline, “dwgsim”, that allows the user to generate simulated read datasets according to their analytical needs (see Supplementary Materials S1, Figure A1b, and Supplementary Materials S2).

**Table 3:** Review of the processes and related workflows currently implemented in the NGS Pipeline. All processes and workflows have been tested and validated and are available for use by interested scientists. A single pipeline can be run independently from others, or can be connected as illustrated by the analytical workflow protocols described in this Table.

Process	Process Description	Software & Algorithms	Input *	Output (Files)	Upstream Module Dependencies	Downstream Module Dependencies
**Preprocessing step**	**Test the NGS raw data and functionality**	homemade script	reads (original solexa format)	subset of reads (fastq format)	**none **	**(1.1) Alignment, (1.2) *De novo* assembly**
**(1.1) Alignment**	**Mapping the reads to the reference genome**	MAQ	reads (binary fastq format)	SAM	**Preprocessing**	**(1.2) Basic QC**
		BWA	reads (fastq format)	SAM		
		BWA-SW (SE only)	reads (solexa format)	SAM		
		PERM	reads (fastq format)	SAM		
		BOWTIE	reads (solexa format)	SAM		
		SOAPv2	reads (fastq format)	SAM		
		MOSAIK	reads (solexa format)	SAM		
		NOVOALIGN	reads (solexa format)	SAM		
**(1.2) *De novo* assembly**	**Build a *de novo* genome sequence**	VELVET	reads (fastq format)	contigs file	**Preprocessing**	**(1.1) Alignment or none ^$^**
		SOAPdenovo	reads (fastq format)	contigs file		
		ABYSS	reads (fastq format)	contigs file		
**(1.3) Basic QC**	**Basic Data formatting and quality control**	PICARD, SAMTOOLS	SAM	BAM	**(1.1) Alignment**	**(1.4) Advanced QC**
**(1.4) Advanced QC**	**QC for advanced issues**	PICARD, SAMTOOLS, GATK	BAM	BAM clean	**(1.3) Basic QC**	**(2.1a) Variant calling (2.1b) CNV analysis**
**(2.1a) Variant calling and annotation**	**Identify and visualize SNPs and Indels from the whole genome**	Sequence Variant Analyzer v1.0	BAM clean	csv files with variants and annotation	**(1.4) Advanced QC**	**Statistical analysis and visualization software ^#^**
		SAMTOOLS and ANNOVAR for annotation	BAM clean	txt files with variants		
		Unified genotyper and ANNOVAR for annotation	BAM clean	txt files with variants		
**(2.1b) CNVs calling**	**Analysis of CNVs (ins & del > 1 Kb)**	BOWTIE CNVer SAVANT	reads (solexa format)	txt file with the CNVs calls	**(1.4) Advanced QC**	**Statistical analysis and visualization software ^#^**
		CNVseq	SAM	txt file with the CNVs calls		**R (stat software)**
		SAMTOOLS ERDS Sequence variant analyzer ERDS v1.0	BAM clean	csv file with the CNVs calls		**Statistical analysis and visualization software ^#^**
**Simulated data generation tool**	Generate simulated reads according to the needs of the user	dwgsim	-	SE or PE .fastq files	-	**(1.1) Alignment, (1.2) *De novo* assembly**

* With solexa format we refer to the Phred quality score code used by the Illumina Pipeline version prior than 1.8 (Phred +64). The newer versions of the Illumina Pipeline produce reads file directly in Sanger format (Phred +33). To guarantee backwards compatibility with data produced by version of the Illumina Pipeline previous than 1.8 we have embedded a conversion step from Solexa FASTQ to Sanger FASTQ for the alignment software that don’t support the solexa format. The user can remove this step in case the conversion is not needed; ^#^ External software like PLINQseq for the statistical analysis or IGV for visualization are not embedded in the workflow; ^$^ If a reference genome is not available the contigs can be used like they are for further analysis. If a reference genome is available the contigs can be aligned back to the reference genome with BWA-SW.

The GPCG pipeline environment allows users to change execution parameters for each software tool represented as a module in the pipeline workflow. This architecture allows changing parameters, adding new parameters or execution controls (e.g., flags, options), modifying values of existent parameters, removing parameters, as well as inserting new processing modules or connections to/from data objects (data-sources/inputs or data-sinks/outputs). These workflow modifications are accomplished directly in the pipeline graphical interface by using mouse-selection and keyboard entries.

### 2.1. Alignment

**Figure 3:**
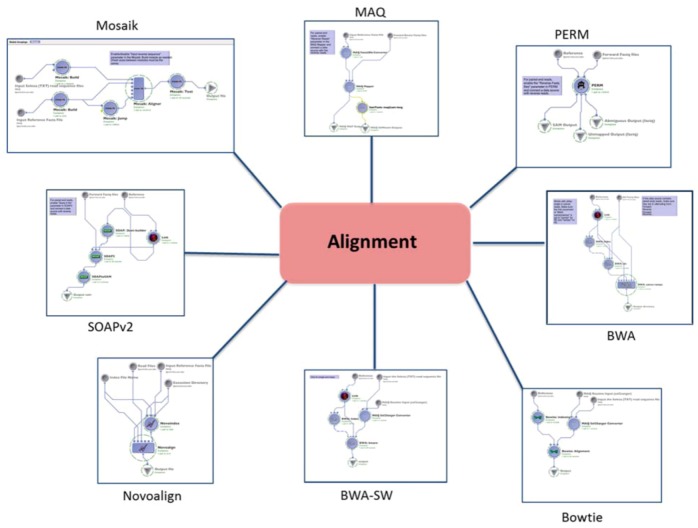
Schematic representation of alignment modules available for both single and paired-end data (2.1).

We incorporated into the LONI pipeline some of the most used alignment software tools (Figure 3). Through the “edit module” functionality of the graphical user interface (GUI) it is possible to visually manage all the parameters of the alignment software (e.g., number of allowed mismatches, read trimming, gap extension) encapsulated by the module itself. Each algorithm in a workflow (other than BWA-SW that runs only in single end) has a switch that allows the user to perform an alignment on either single end (SE) or paired end (PE) reads. The input reads are generally in FASTQ format. BWA accepts input read sequences also in BAM format. For paired-end data the pair reads need to be grouped together in one single BAM file. The final output is an alignment file is in SAM format (see Supplementary Materials S1, Figure A2). This file is ready to undergo quality control procedures. The choice of the alignment software is critical as different software might lead to different results in term of mapping and downstream variants calling. It is strongly suggested to perform the alignment using more than one software and identify strategies that may provide better fit for the user’s data. BWA and BOWTIE, for example, are among the fastest alignment algorithms for both single and paired end reads, and are particularly suitable for whole genome alignments. They generally report by only one alignment hit, and in the default mode do not guarantee finding the best hit nor if the found hit is unique. In particular, for users dealing with highly repetitive genomic regions, it is possible to force these algorithms to output all the possible hits with a high cost in terms of speed, or to use a probabilistic software like PERM that outputs all the found hits. The user could perform a *de novo* assembly (see next paragraph) for a better resolution in such regions. If the main interest is the detection of CNVs with paired-end reads, Novoalign is strongly recommended because of the high mapping accuracy, even if slower than the previously mentioned Burrows-Wheeler (BW) based software.

For selecting parameters settings, users can find hints about the meaning and possible values of each single parameters by double-clicking on a parameter icon or via the execution info menu-accessible as a right-click on the module of interest. For the parameters tuning the users have to refer to the software documentation as all the algorithms works differently. The LONI Pipeline workflow environment enhances the user’s abilities to optimize the selection of the parameters for each tool by allowing specification of a list containing a range of parameters spanning the support of each parameter of interest. This *parameter-optimization* strategy is exactly the basis of the development of meta-algorithms as workflow protocols [76,77,78].

The quality control process we used to determine the tools to be incorporated is based on an up to date and detailed investigation of the available NGS tools, including research papers with technical and analytical details, and the online discussions panel/forums if available to better follow the analytical trends and open issues. The testing with simulated and real data provided us further hints into the software reliability. Some information and guidelines to help in identifying the best set of software to analyze different type of data can be found in some about reference with comparisons among tools we provided [22,79].

### 2.2. Assembly

**Figure 4:**
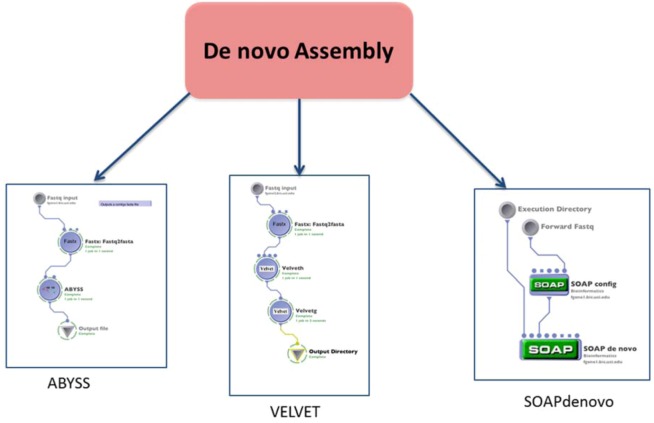
Schematic representation of the *de novo* assembly workflows available (2.2).

When a reference genome is not available for the species under investigation it is necessary to perform *de novo* assembly of the reads. *De novo* assembly is also the suggested approach for reads mapping in regions prone to rearrangements, rapidly evolving, or where the reference genome might not be informative. We have developed three workflows embedding the most common *de novo* assembly software based on de Bruijn graphs (Figure 4), which works both with single and paired end data. One of the most important parameters to be tuned in *de novo* assembly is the fixed-length of the subsequences used to build the graphs length, which is abbreviated as k-mer. The most efficient k-mer size for a particular assembly is determined by the read length as well as the error rate, and it may be estimated *a priori* using “ad hoc” tools provided by the software developer or by evaluating the results obtained through varying the k-mer size across different runs. Some assemblers are also able to output scaffolds (*i.e.*, a set of contigs with known relative orientation and distance) or supercontigs (*i.e.*, contigs in which gaps are allowed). When *de novo* assembly is used in complex region of organisms with a known reference genome, the contigs can be aligned back to it with the BWA-SW workflow, and then undergo the subsequent analytical steps.

### 2.3. Quality Control Improvement of Reads

#### 2.3.1. Basic Quality Control and File Formatting

**Figure 5:**
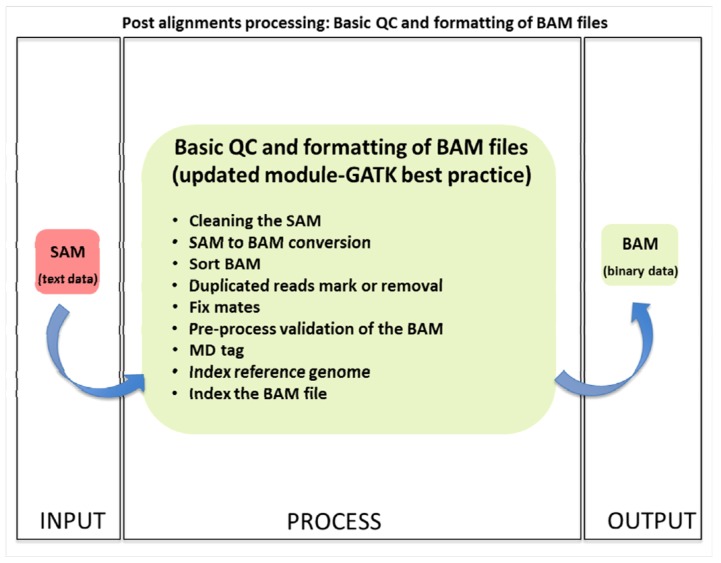
A snapshot of the general organization of the Basic QC workflow (2.1.3). After an initial file cleaning that performs various fix ups, the alignment file in Sequence Alignment/Map (SAM) format is converted in Binary Sequence Alignment/Map (BAM) file and sorted. The workflow takes care of the duplicated reads removing or marking the potential PCR duplicates. If multiple read pairs have identical external coordinates, it only retains the pair with highest mapping quality. This step is particularly suited for paired end data and the user can switch between the two options simply changing the REMOVE_DUPLICATES argument in the GUI related to this module. The removal step can be excluded from a workflow run depending on the interest in studying repetitive elements. In case of paired end reads, the pipeline then ensures that all mate-pair information is in sync between each read and its mate pair, fixing any incoherent information. The BAM file undergoes MD tagging that adds string, labeling the mismatching positions. The BAM is finally indexed using the index of the reference genome.

After the alignment the reads undergo first basic formatting and quality controls steps. We have developed a “Basic QC” workflow in line with the consolidated and updated procedures for DNA-Seq analyses (Figure 5).

The output of this module is a clean, sorted, indexed BAM file that can undergo advanced QC procedures, or used as it is for downstream analyses.

#### 2.3.2. Advanced QC

**Figure 6:**
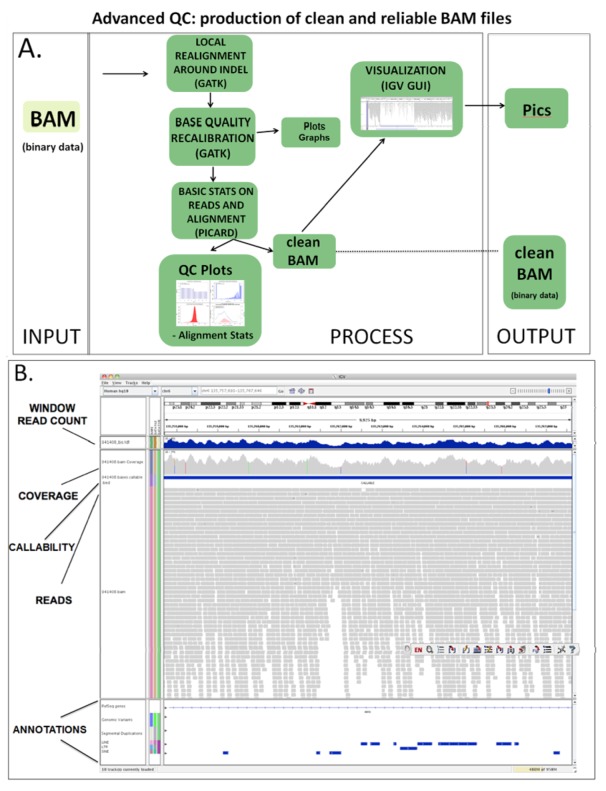
A snapshot of the general organization of the Advanced QC workflow (2.1.4). (**A**) After the basic QC, the reads that map within Indels in the individual’s genome compared to the reference genome are locally realigned, as they may lead to alignment artifacts that can easily be misinterpreted as SNPs. The next step is the base quality score recalibration to recalibrate base quality scores of reads, by the analysis of the covariation among several features of a base (e.g., reported quality scores, the position within the read). The workflow produces plots and tables with the most important metrics for a DNA-Seq experiment (*i.e.*, mean quality by cycle, insert size metrics, quality score distribution, GC-bias metrics, main alignment metrics) with the PICARD software; (**B**) The users can then produce useful tracks for the visualization of the data in Integrative Genome Viewer (IGV). Examples are the (a) callability track (*i.e*., evaluates how much a region can be trusted in term of coverage, accuracy and quality by GATK and can be visualized as a bar chart in IGV); (b) the sliding window coverage (*i.e.*, a computation of average alignment over a specified window size across the genome with igvtools). The main outputs of this step are: (1) a cleaned BAM file ready to be used for variant calling, (2) a set of plots and text files that can help the user to have a general picture about the general quality of the experiment and (3) a set of track files to visualize the dataset and its features. The user can upload the indexed BAM files and these tracks in IGV to visualize and annotate the reads across the whole genome with user-produced or online tracks (RefSeq, RepeatMasker, Database of Genomic Variants).

Additional advanced QC issues can be addressed and fixed using the GATK toolkit (http://www.broadinstitute.org/gsa/wiki) [27,80]. The GATK developers suggested a core analytical framework that includes local realignment around Indels and base quality score recalibration [27,80]. We have embedded these processes in the “Advanced QC” workflows, adding other modules to produce quality control plots, statistics, and tracks useful for the visualization of the data (Figure 6).

### 2.4. Variant Calling and Annotation

#### 2.4.1. SNPs and Indels Calling and Annotation

**Figure 7:**
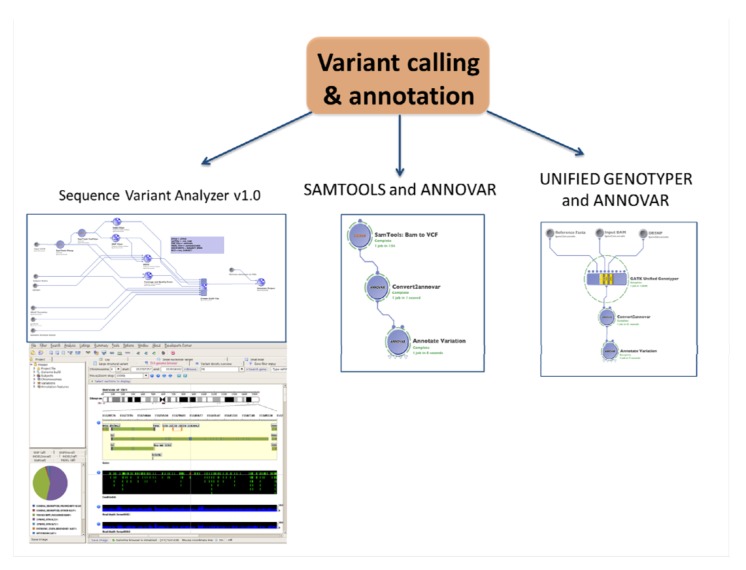
A snapshot of the three independent workflow for variant calling, and annotation workflows available. Sequence Variant Analyzer (SVA) displays a graphical user interface (GUI) to visualize, annotate, filter and analyze the called variants.

We explored and embedded in workflow at least three different frameworks to call SNPs and Indels from whole genome alignment data and produce a comprehensive mutation/functional analysis report (Figure 7).

For the Sequence Variant Analyzer (SVA, http://www.svaproject.org [51]) v1.0 workflow, after SNPs and Indels have been called with SAMTOOLS [13] (http://samtools.sourceforge.net/) and CNVs have been called with “Estimation by Read Depth with Single Nucleotide Variants” (ERDS) software v1.02 (http://web.duke.edu/~mz34/erds.htm), they undergo annotation and visualization through SVA (Figure 7). SVA is a visualization platform for performing statistical analysis and filtering procedures as well. The workflow we have developed allows users to produce a .gsap file, which can be loaded into SVA to create a project with single or multiple annotated genomes. This version of SVA is linked to ENSEMBL hg18 annotations.

Variants detected with SAMTOOLS [13] can be also exported as a VCF file (Table S1) and comprehensively annotated through ANNOVAR [50] (Figure 7). This software allows functional annotation of genetic variants detected from diverse genomes (human genome hg18, hg19, as well as mouse, worm, fly, yeast and many others). In particular the last release of ANNOVAR retrieves variant calls and frequency information from the 1000 Genomes Project [81] or from the sixty genomes released by Complete Genomics [30]. ANNOVAR offers three different annotation options: *gene-based*, *region-based* or *filter-based annotation*, and all the three options are implemented.

In the GPCG we also embedded the UnifiedGenotyperV2 from the GATK suite, which is a popular software to simultaneously call SNPs and Indels and produce a VCF output file [13] (Figure 7). We connected this module with ANNOVAR to perform the complete annotation of variants, as previously described.

#### 2.4.2. CNVs Calling

**Figure 8:**
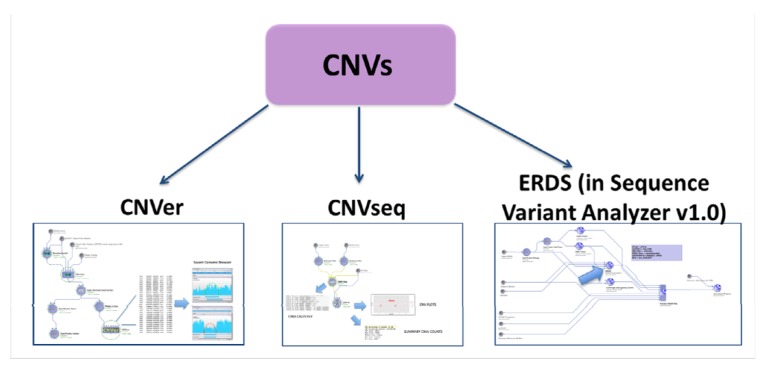
A snapshot of the general organization of the CNVs modules. ERDS, CNVer and CNVseq have been implemented as a first wave of tools to call CNVs in DNA-Seq data.

While general agreement on the best analytical strategy is still lacking [2], we have implemented some workflows, as a first wave of a more comprehensive set of tools. Among the many different approaches to call CNVs, we have chosen the three approaches described in Figure 8.

CNVer relies both on read depth and read pair information [48] in a computational framework called the donor graph, that reduces the sequencing biases causing uneven local coverage (Figure 8). The most interesting feature of CNVer is the ability to compute the absolute copy counts of segments of the donor genome, and work with low coverage datasets. Moreover CNVer allows detecting CNVs without the need of a reference genome. The CNVs called by CNVer may be imported and visualized in the SAVANT genome browser [82] (Supplementary Materials S1, Figure A18).

CNVseq is a read depth method that detects CNVs with a robust statistical model conceptually derived from the aCGH (array Comparative genomic hybridization) analytical framework. [47]. CNVseq uses a sequence as a template and two sets of reads, one set from a reference individual (e.g., the individual expected to show a normal ploidy) and one from the test individual we want to screen for CNVs*.* The two sets of reads are aligned to a template genome, and then with a sliding window approach, CNVs are detected by computing the number of reads for each individual in each sliding window, yielding ratios and copy number estimations. Additional steps with this approach are performed in R (http://www.r-project.org/), with the possibility to get a full visualization of the log_2_ allelic ratio profiles and statistics on the detected CNVs (Supplementary Materials S1, Figure A20).

The final CNV workflow method is ERDS, which is a Hidden Markov Model (HMM) based approach that relies on read depth to infer the copy number state. It represents an extension of the method described in Bentley *et al*. (2008) [31], and described more in detail by Pelak *et al*. (2010) [45]. Expected read depth is calculated using the expectation maximization (EM) approach and corrected by GC bias. The ERDS functionality can be found embedded into the already described SVA 1.0 workflow for SNPs and Indels calling (Section 2.4.1) can then be used to visually inspect and annotate the CNVs (Supplementary Materials S1, Figure A21).

### 2.5. Evaluation with Simulated Data

We evaluated all the previously described GPCG workflows (Table 2 and Supplementary Materials S2) with 30 million simulated reads (both SE and PE) generated with dwgsim simulation tool, a publically available utility for simulating whole-genome Illumina reads [83]. Runtimes and information about the performances of the workflow with simulated data are summarized in Supplementary Table S2. We ran all the workflows with default parameters. 

We reported for all the modules a better time performance of the GPCG compared to Galaxy, together with the absence for GPCG of the time required for the data upload.

Using the same simulated data files, we tested the common modules embedded both in our GPCG and on the Galaxy webserver interface. Due to the structure of Galaxy and the reduced number of processes available, we couldn’t compare its performances on a workflow scale. The module shared by GPCG and Galaxy were: conversion of solexa into sanger format, BWA and Bowtie paired end alignment, the PICARD utilities to fix the mate information, mark the duplicates, collect the alignment, GC bias, and insert size metrics. We reported the GPCG and Galaxy time performances in Table 4. 

**Table 4:** Runtimes and performances on simulated data for modules in common across Graphical Pipeline for Computational Genomics (GPCG) and Galaxy. The performances of GPCG in terms of run time were better than Galaxy for all the tested modules.

Analytical category	Input file(file size)	Job description	GPCG workflow name	Time	Galaxy module name	Time
**Data upload**	2.4 Gb × 2 (PE)	Upload of the data into the webserver	(N/A)	(N/A)	Upload of the data	180 min
**Preprocessing**	2.4 Gb fastq file	Conversion of solexa into sanger format	Preprocessing pipeline: sol2sanger	6 min	FASTQ Groomer	45 min
**Alignment**	2.4 Gb × 2 fastq files (PE)	BWA paired end alignment with default parameters	BWA PE (1.1)	132 min	Map with BWA for Illumina	240 min
	2.4 G × 2 fastq files (PE)	Bowtie paired end alignment with default parameters	BOWTIE PE (1.1)	205 min	Map with Bowtie for Illumina	270 min
**Quality control (metrics and cleaning)**	1.6 Gb SAM file	Synchronization of mate-pair information	Fix Mate Information (Basic QC, 1.3)	6 min	Paired Read Mate Fixer for paired data	30 min
	1.6 Gb SAM file	Marks duplicate reads	Mark Duplicates (Basic QC, 1.3)	2 min	Marks duplicate reads	20 min
	1.6 Gb SAM file	Reports the alignment metric of a SAM/BAM file	Collect Alignment Summary Metrics (Advanced QC, 1.4)	2 min	SAM/BAM Alignment Summary Metrics	6 min
	1.6 Gb SAM file	Reports the SAM/BAM GCbias metrics	Collect GC Bias Metrics (Advanced QC, 1.4)	3 min	SAM/BAM GC Bias Metrics	7 min
	1.6 Gb SAM file	Reports the insert size metrics	Collect Insert Size Metrics (Advanced QC, 1.4)	2 min	Insertion size metrics for PAIRED data	6 min

All of the performances of the GPCG platform were better compared to the run time of Galaxy. Furthermore, a bottleneck in the timing of analysis is the upload of to the Galaxy webserver, together with the not predictable waiting in queue time for the processes (Table 4). As the status of the process cannot be checked during the execution, the user must wait the end of the run of a process to input the results in to the next step without any hint about the elapsed time, and without the possibility to automatically keep track of the run times.

### 2.6. Evaluation with Real Data

**Figure 9:**
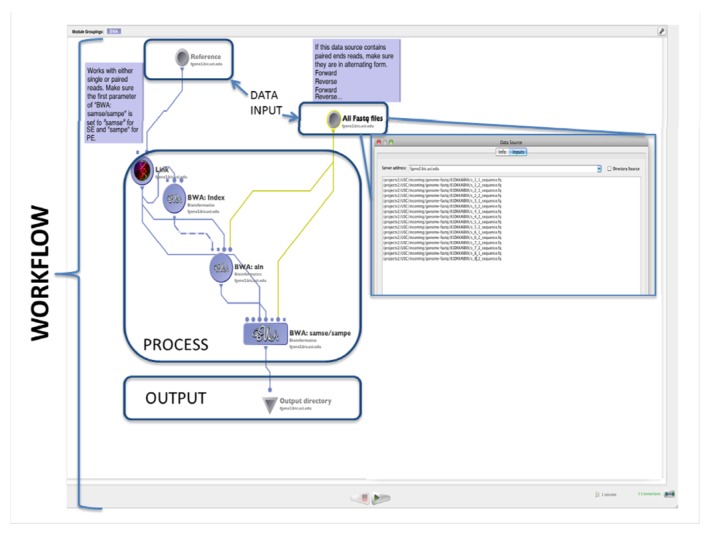
Snapshot from the module we used to run the alignment of an entire flowcell with BWA-PE. This workflow includes the indexing of the reference genome (BWA: Index), the alignment of the two reads separately (BWA-aln) and their final combination (BWA: samse/sampe). The sixteen input files (*i.e.*, one forward and one reverse read for each one of the eight lanes of the flowcell) are shown in the data source panel magnified on the right. The pipeline allows managing all the options of the BWA alignment software through the module’s GUI without worrying about complex command lines.

We also evaluated the performance of our GPCG workflows in a “real life scenario”. Supplementary Table S2 reports the performances of the whole-genome alignment with BWA of an entire Illumina flowcell with an average of 130 million Illumina PE reads per lane. The average time required for the alignment of one lane was 6 hours, and for the whole flowcell was roughly 48 hours using GPCG. The output of the alignment was one SAM alignment file for each lane. In all large scale NGS data analysis it is important to manage the flow of both data and input/output files as they traverse complex workflows. The GPCG provides a simple way to set up the input file section (see magnified data source panel on the right, Figure 9), manage the naming of output files from individual modules using the transformation tool within a module definition (Figure 9). 

## 3. Discussion

The GPCG was developed with a flexible graphical interface for efficient biomedical computing and distributed informatics research [65], and is intended to satisfy the needs of geneticists and computational scientists who are interested in whole genome, exome and targeted sequencing. The graphical workflows we have developed save researchers the time needed to implement and test the command lines of individual analytical steps, logically group complex operations into re-usable units, and allow these units to be aggregated into larger analytical workflows (e.g., QC following alignment).

The GPCG offers several advantages if compared to the available workflow environments for DNA-Seq data analysis. The GPCG includes a set of pipelines ready-to-go with modules logically interconnected between each other on the basis of the current analytical trends, while others offer a set of single modular routines that the user cannot connect in workflows. Moreover the LONI environment doesn’t display any restrictive upper space limits on storage and on the available per-process RAM, and even more importantly doesn’t require any data upload, thus eliminating bottlenecks with data staging to/from the servers. The limit of 100 GB of storage and 64 GB of RAM in Galaxy, together with the unpredictably long data upload time, don’t fit well with the needs of users analyzing whole genome sequencing data, as the overall size of the original forward and reverse fastq files for a single whole 30× genome sequenced in paired end is already ~100 GB. We tested the single modules embedded both in Galaxy and GPCG with the same input files and analytical parameters (Table 4). The performance of the GPCG in term of run time was better compared to Galaxy for all the modules, with the additional time saved in GPCG (~3 hours) as no data upload on the system is required. The user of the GPCG has also the ability to disconnect and reconnect to running workflows, and to monitor at any time the progress and to check the status of a previously initiated process, with a detailed and interactive report of time performances and job execution info (e.g., output and error streams).

Since GPCG is an open source, the user can access the current version of the GPCG pipeline online at http://pipeline.loni.ucla.edu. The entire newly developed computational-genomics infrastructure LONI pipeline includes the pipeline server (http://pipeline.loni.ucla.edu/DPS), the pipeline web-start server (http://pipeline.loni.ucla.edu/PWS), the genomics workflows (http://pipeline.loni.ucla.edu/services/library-navigator/), collaborative wiki documentation for these protocols (http://ucla.in/pbMgUm), and community support (http://informatics.googlecode.com/). All these resources have been developed and are currently supported via an open and collaborative infrastructure. Constructive utilization of diverse tools and computational expertise may be shared as pipeline workflows between professionals, novice users and trainees [65,70].

In this first release of the GPCG we embedded only some popular tools for managing and analyzing DNA-Seq data from the initial raw reads to variant calling and annotation. However, to overcome this limitation, we are regularly testing and adding tools to be shared in the future releases of the genomics pipeline. Also the users can integrate new processes and implement new workflows promoting a community-based protocol validation and openly share and disseminate knowledge, tools and resources.

## 4. Methods

### 4.1. The LONI Environment and Workflow Creation

To translate DNA-Seq data analysis into a graphical pipeline solution within the LONI environment [65] we initially described all global processes in the *protocol design* or* skeletonization* step, using a top-down approach. We outlined the general classes of sequence data analysis, then the appropriate sub-classes of analyses, specific tools, test-data, invocation of concrete tools, and a detailed example of executable syntax for each step (http://pipeline.loni.ucla.edu/support/user-guide/building-a-workflow, [65]). The previously mentioned analytical steps are constructed from a series of command line executable processes, referred to as *modules* or *nodes*, which are connected to each other to form a visual workflow analysis protocol. All the logically concatenated *modules/nodes* involved in the same analytical step (e.g., alignment) comprise a workflow. 

After all necessary modules that make up a workflow are independently defined and validated through the LONI pipeline GUI interface (http://pipeline.loni.ucla.edu/support/user-guide/creating-modules/), they are integrated into a coherent workflow.

### 4.2. Accessibility of the GPCG

**Figure 10:**
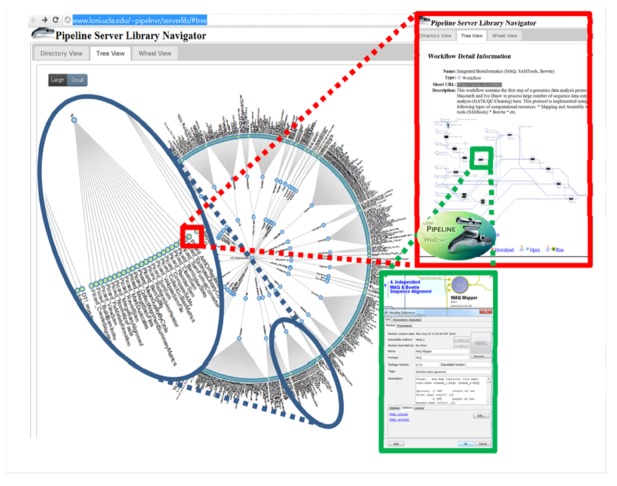
The LONI pipeline computational library Navigator allows the interactive traversal, inspection, downloading and utilization of specific NGS analyses. Nested insert images illustrate the most common steps of search, selection, comparison, modification and execution of available end-to-end computational genomics workflows.

The user can access the current version of the GPCG online at http://pipeline.loni.ucla.edu. The local Client can be set up by the users following the instructions reported at http://pipeline.loni.ucla.edu/support/user-guide/installation/. The GPCG can also be deployed on a server base downloading the distributed pipeline server installer (DPS) (http://pipeline.loni.ucla.edu/support/server-guide/installation/), and following the instructions available at http://pipeline.loni.ucla.edu/DPS). The GPCG workflows can also be directly launched via the pipeline web-start server (PWS) (http://pipeline.loni.ucla.edu/PWS). To search across the entire set of workflows the user can rely on an interactive graphical navigator (http://pipeline.loni.ucla.edu/services/library-navigator/), which enables not only the discovery, but also web-based utilization of this new computational-genomics infrastructure through the web, Figure 10.

### 4.3. Evaluation of the GPCG Workflows with Simulated and Real Data

Table 1 presents the software we have embedded in the workflows released with GPCG. We evaluated the workflows with both real and simulated data (Supplementary Table S2).

*Evaluation with simulated data* We used dwgsim [84], a utility for whole-genome Illumina reads simulation, contained in DNAA v0.1.2 (http://sourceforge.net/projects/dnaa/), to generate Illumina-like short sequences, using the default empirical error model illustrated on DNAA’s Whole-Genome Simulation web-site (http://dnaa.sf.net). In total we generated 30 million reads with 100 bp length, using the complete human genome (hg18) as a reference and with default parameters. We developed also a module that allows the users to generate simulation datasets according to their needs (see Table 2 and Supplementary Materials S2). 

*Evaluation with real data* To further compare the behavior of our workflows on real applications, we used an entire flow cell (8 lanes) with an average of 130 million Illumina PE reads per lane with length of 100 bp (fastq produced with the Illumina Pipeline v1.8) to be aligned by BWA-PE against the whole human genome sequences (assembly: NCBI36.1/hg18). 

These experiments were produced on an Illumina HiSeq200 DNA sequencer using v5 kits.

All the testing runs were performed on a Linux server Dual Intel Xeon X5650 2.67 Ghz 6 core processors 96 GB’s of RAM 2 × 10 k RPM 150 GB WD VelociRaptor RAID 0 RHEL 5 64-bit. 

## 5. Conclusions

The availability of workflows to manage and analyze NGS data in a straightforward way may play a role in triggering genomic advances in human health related translational research in psychiatry genetics. Currently, NGS technology is emerging as a fundamental basis on which to understand disease complexity and heterogeneity both for common and rare diseases, with benefits to clinical diagnostics and care once research findings are translated into clinical tests. Sequencing clinical subjects is becoming a method of choice in translational studies of diseases, and genetic defects underlying several genetic disorders have been identified through whole exome or whole genome sequencing [85,86]. Nevertheless, an understanding of how genome variability leads to disease pathogenesis is still far from complete for the vast majority of genetic diseases [56], at least as to the meaning of many variants present in the genome of healthy individuals. The computational challenge for DNA-Seq data analysis is often a bottleneck, as many different tools are constantly emerging, and often requiring bioinformatics skills. We are proposing the GPCG as a handy and helpful graphical analysis platform to improve the efficiency of high-throughput data analysis in diverse applications of DNA-Seq analysis projects. There are two tiers of validations for the proposed pipeline GPCG pipeline infrastructure. The first tier is validation of the technical protocols (as we compared the results of the GPCG pipeline protocol against Galaxy). This evaluation confirms the programmatic reliability and reproducibility of the results using identical computational libraries. However, the second tier of validation is more important as it provides scientific evidence of the value added by the new GPCG pipeline infrastructure. This scientific validation includes for example the comparison of results obtained with different algorithms in term of read mapping, variant calling and quality control procedures to find the process that best fits the data, together with improvements in speed and high-throughput volume processing. For example, the GPCG framework may be used to construct a new genomics computing protocol that explicitly utilizes specific sequence analysis tools. The entire pipeline protocol may be shared with other users who can easily plug-in new data and/or swap alternative modules for analogous processing steps (employing different software tools). Such multi-investigator experimental studies would provide cues about how to select appropriate software tools and how different libraries compare in processing different type data sets (e.g., varying read length, fragments or paired ends, highly repetitive genome, *etc*.)

## Supplementary Files

Supplementary File 1 ZIP-Document (ZIP, 7447 KB)
