# Supplementary material for: Next Generation Sequence Analysis and Computational Genomics Using Graphical Pipeline Workflows
Source: Genes (Basel). 2012 Aug 30;3(3):545–75. doi: 10.3390/genes3030545 (PMC3490498; doi:10.3390/genes3030545)
Supplement: Supplementary File 1 — ZIP-Document (ZIP, 7447 KB) [file genes-03-00545-s001.zip › Supplementary/SuppMat_S1.docx]

**Supplementary Materials S1 (for the complete documentation with the detailed description of all the modules see Supplementary Materials S2)**

#### Preprocessing modules

**-Extraction of a subset of reads**

This module can extract a subset of reads to perform validation and initial testing of modules before running analysis on an entire dataset. This module also can convert the reads in Solexa FASTQ format to Sanger FASTQ format, and to binary Sanger FASTQ as required by some aligners like MAQ. The user can specify the number of lines to include in the subset through the GUI.


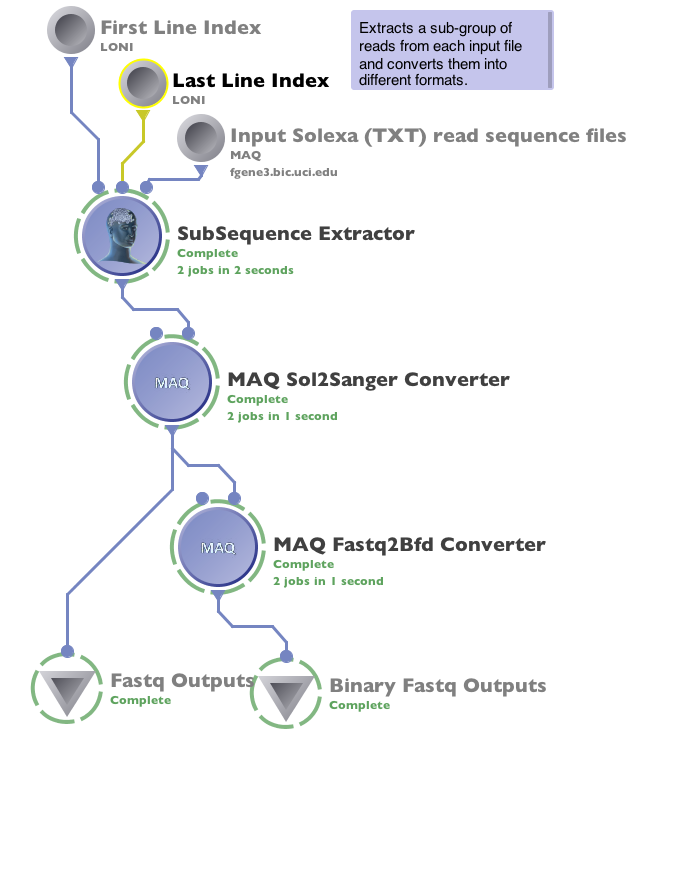


**Figure A1a**: A snapshot of the preprocessing pipeline that extract a subset of reads to be tested.

Note that this ‘whole’ module is directly compatible with MAQ. Some of the embedded routines are useful ‘per se’, and may be helpful to the user to perform format conversion operations. To encounter these needs, we then made available also the single independent modules for SOLEXA2FASTQ, FASTQ2BFQ, FASTA2BFA conversion (see more in Supplementary Materials).

**-Generation of simulated reads**

This module is helpful for generating simulated read datasets (both SE and PE) in terms of number of reads, read length and base quality scores (specified as parameters of the module through the GUI). The output is a FASTQ file that can be used for subsequent alignment/de novo assembly steps.


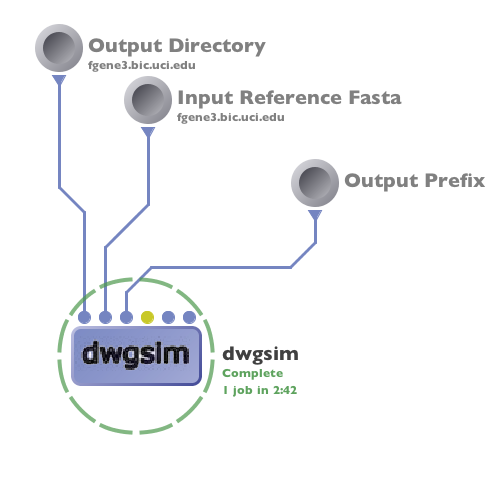


**Figure A1b**: A snapshot of the pipeline to generate simulated datasets.

#### Alignment and de novo assembly (SE and PE) modules

#### (1.1) Alignment (SE and PE) modules

In this section 1(a) we describe in detail all the modules we have developed for the alignment of the reads, with a more detailed description of the BWA pipeline as an example of the general structure (see Figure A2).


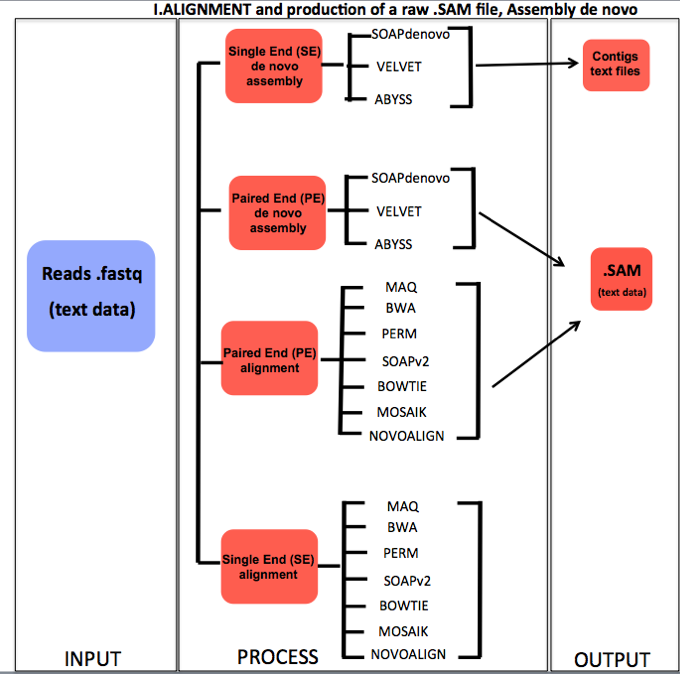


**Figure A2**: A snapshot of the general organization of the aligners and the novo assembly modules.

- **BWA**
  - **URL**: http://bio-bwa.sourceforge.net
  - **Description**: Burrows-Wheeler Alignment tool (BWA) is based on backward search with Burrows-Wheeler Transform (BWT) and allows aligning short sequencing reads against a reference sequence, allowing mismatches and gaps. Is designed for short queries up to ~200bp with low error rate (<3%). Indexing Genome with Suffix Array/BWT
  - **Pipeline Workflow**

*Name*:

*URL*:

*Screenshots*:

- - - Input: **Figure A3** shows a snapshot of the input parameters (data-sources) for the corresponding Pipeline workflow.


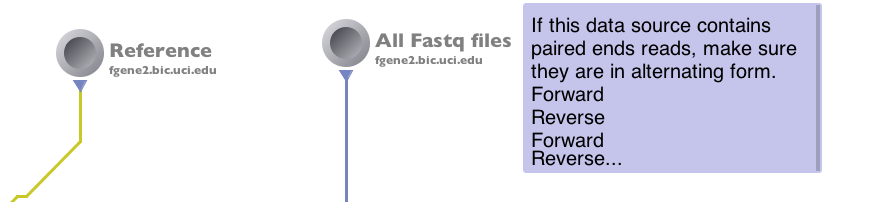


**Figure A3**: A snapshot of the input parameters (data-sources) for the BWA Pipeline workflow.

Pipeline Execution: **Figure A4** shows the completed BWA pipeline workflow.

- -
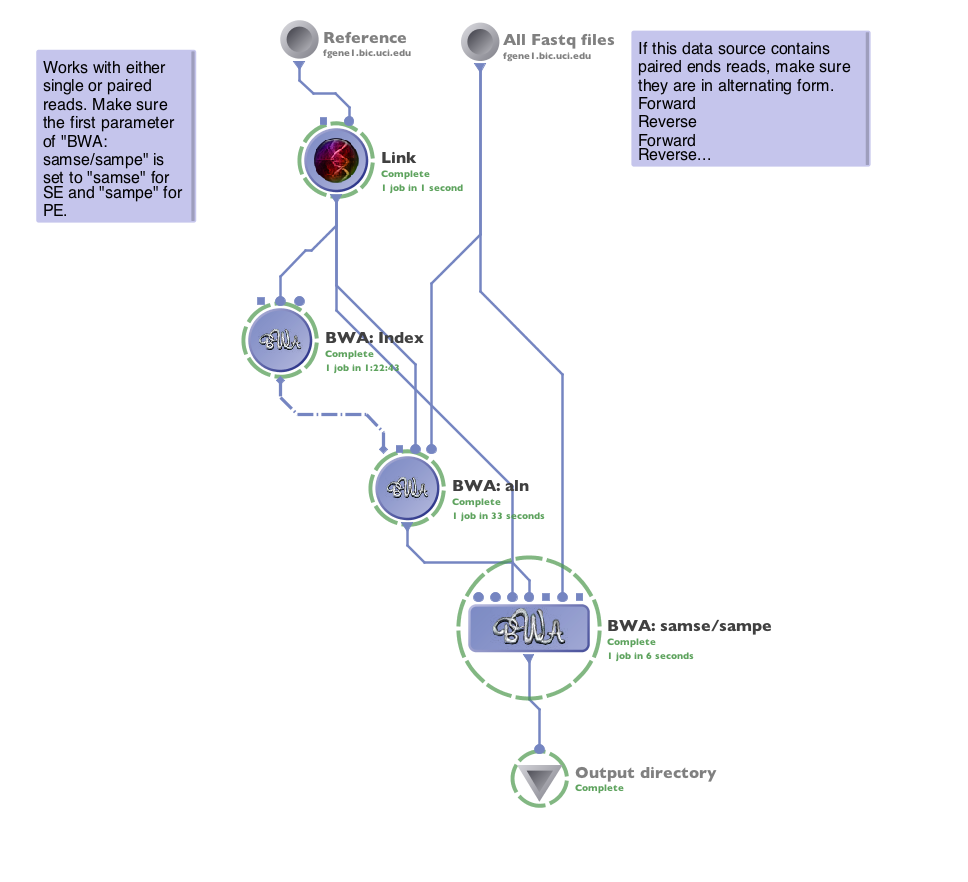


**Figure A4**: A snapshot of the completed BWA (SE and PE have the same appearance) Pipeline workflow.

- - - Output: Sequence Alignment Map (SAM) files (see **Table S1**)
- **MAQ**
  - **URL**: <http://maq.sourceforge.net/>
  - **Description**: Mapping and Assembly with Qualities (MAQ) builds assemblies by mapping shotgun short read sequences to a reference genome, using quality scores to derive genotype calls of the consensus sequence. Reads are indexed in a hash table
  - **Pipeline Workflow**

*XML Metadata description*:

*Name*:

*URL*:

- - - Input: Binary FASTQ files (produced starting from Solexa FASTQ files with the sol2bfq pipeline using FASTQ sol2sanger and fastq2bfq routines).
    - Output: Sequence Alignment Map (SAM) files (see **Table S1**)
- **BWA-SW**
  - **URL**: http://sourceforge.net/projects/bio-bwa/files/
  - **Description**: based on the Burrows-Wheeler Transform (BWT), BWA-SW, is designed for long reads with more errors. It performs heuristic Smith-Waterman-like alignments to find high-scoring local hits. On low-error short queries, BWA-SW is slower and less accurate than the first algorithm, but on long queries, it shows better performances. Genome Indexed with Suffix Array/BWT
  - **Installation instructions**: http://bio-bwa.sourceforge.net
  - **Pipeline Workflow**

*XML Metadata description*:

*Name*:

*URL*:

- - - Input: Solexa FASTQ files
    - Output: Sequence Alignment Map (SAM) files (see **Table S1**)
- **PERM**
  - **URL**: http://code.google.com/p/perm/
  - **Description**: PerM uses a special periodic seed-matching method that has speed advantages for longer reads (limited to 64bp currently), non-mappable reads (for fixed number of shift and checking) and genome-wide mapping due to the high seed weight. Genome indexed with hash tables.
  - **Pipeline Workflow**

*XML Metadata description*:

*Name*:

*URL*:

- - - Input: FASTQ files (produced from Solexa FASTQ files and the sol2sanger MAQ routine)
    - Output: Sequence Alignment Map (SAM) files (see **Table S1**)
- **BOWTIE**
  - **URL**: http://bowtie-bio.sourceforge.net/
  - **Description**: BOWTIE is a fast, memory-efficient short read aligner that allows quick alignments of large sets of short DNA sequences to large genomes. Genome indexed with Suffix Array/BWT.
  - **Pipeline Workflow**

*XML Metadata description*:

*Name*:

*URL*:

- - - Input: Solexa FASTQ files
    - Output: Sequence Alignment Map (SAM) files (see **Table S1**)
- **SOAPv2**
  - **URL**: http://soap.genomics.org.cn/soapaligner.html
  - **Description**: SOAPaligner/soap2 is a member of the SOAP (Short Oligonucleotide Analysis Package). It is an updated version of SOAP software for short oligonucleotide alignment. The new program provides fast and accurate alignments for large datasets of short reads generated by Illumina/Solexa genome sequencers. It supports a wide range of read lengths. Genome indexed with Suffix Array/BWT.
  - **Pipeline Workflow**

*XML Metadata description*:

*Name*:

*URL*:

- - - Input: FASTQ files (produced from Solexa FASTQ files and the sol2sanger MAQ routine)
    - Output: Sequence Alignment Map (SAM) files (see **Table S1**)
- **MOSAIK**
  - **URL**: http://bioinformatics.bc.edu/marthlab/Mosaik
  - **Description**: MOSAIK produces gapped alignments using the Smith-Waterman algorithm. Genome indexed with hash tables.
  - **Pipeline Workflow**

*XML Metadata description*:

*Name*:

*URL*:

- - - Input: Solexa FASTQ files
    - Output: Sequence Alignment Map (SAM) files (see **Table S1**)
- **NOVOALIGN**
  - **URL**: http://www.novocraft.com/main/index.php
  - **Description**: aligner for single-ended and paired-end reads from the Illumina Genome Analyzer. Novoalign finds global optimum alignments using full Needleman-Wunsch algorithm with affine gap penalties. Indexing Genome with Hash Tables.
  - **Pipeline Workflow**

*XML Metadata description*:

*Name*:

*URL*:

*Screenshots*:

- - - Input: Solexa/Sanger FASTQ files
    - Output: Sequence Alignment Map (SAM) files (see **Table S1**).

#### (1.2) Assembly de novo modules

In the following section we describe the de novo assembly workflows we have developed, with a detailed description of VELVET as an example of the general structure of the other workflows.

- **VELVET**
  - **URL**: http://www.ebi.ac.uk/~zerbino/velvet/
  - **Description**: Velvet [[95](#_ENREF_95)] is a de novo assembler constructing de Bruijn graphs [[96](#_ENREF_96)] for genomic sequence assembly.
  - **Pipeline Workflow**

*XML Metadata description*:

*Name*:

*URL*:

*Screenshots*:

- - - Input: **Figure A5** shows a snapshot of the input parameters (data-sources) for the corresponding Pipeline workflow ((A) SE, (B) PE).


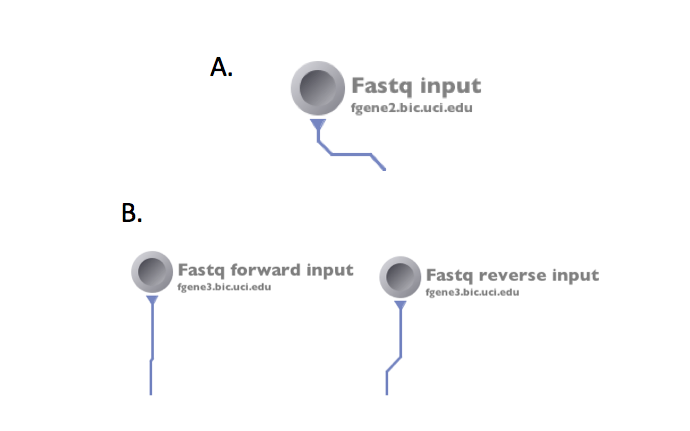


**Figure A5.** A snapshot of the input parameters (data-sources) for the VELVET Pipeline workflow (a) SE and (b) PE.

- - - Pipeline Execution: **Figure A6** shows the completed VELVET pipeline workflow.


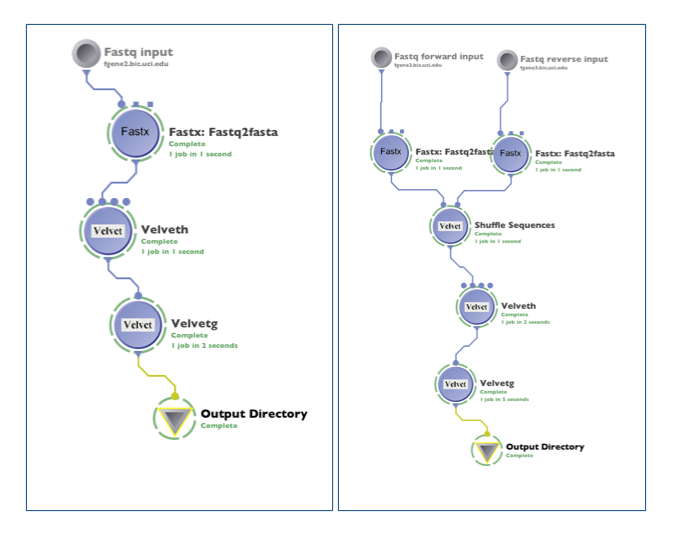


**Figure A6**: A snapshot of the completed VELVET (A) SE and (B) PE Pipeline workflow.

- - - Output: a .FASTA file containing the assembled contigs (see **Table S1**)
- **SOAPdeNOVO**
  - **URL**: <http://soap.genomics.org.cn/soapdenovo.html>
  - **Description**: SOAPdenovo [[97](#_ENREF_97)] is a novel short-read assembly method that can build a de novo draft assembly for the human-sized genomes.
  - **Pipeline Workflow**

*XML Metadata description*:

*Name*:

*URL*:

- - - Input: FASTQ file (produced from Solexa reads as a FASTQ and converted with the sol2sanger MAQ routine)
    - Output: a FASTA file containing the assembled contigs (see **Table S1**)

**ABYSS (Assembly By Short Sequences)**

- - **URL**: <http://www.bcgsc.ca/platform/bioinfo/software/abyss>
  - **Description**: ABySS [[98](#_ENREF_98)] is a de novo, parallel, paired-end sequence assembler that is designed for short reads.
  - **Pipeline Workflow**

*XML Metadata description*:

*Name*:

*URL*:

- - - Input: FASTQ file (produced starting from Solexa reads as a FASTQ file and converted with the sol2sanger MAQ routine)
    - Output: a FASTA file containing the assembled contigs (see **Table S1**)

#### (1.3) Basic QC modules

- - **URL**: SAMTOOLS (<http://samtools.sourceforge.net/>); PICARD (http://picard.sourceforge.net/)
  - **Pipeline Workflow**

*XML Metadata description*:

*Name*:

*URL*:

*Screenshots*:

- - - Input: **Figure A7** shows a snapshot of the input parameters (data-sources) for the corresponding Pipeline workflow.


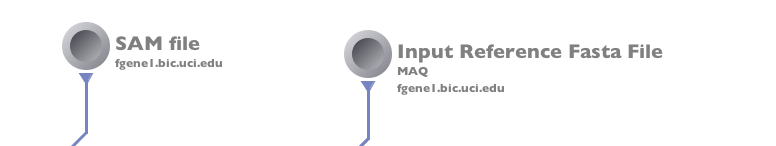


**Figure A7**: A snapshot of the input parameters (data-sources) for the BasicQC1 Pipeline workflows.

- - - Pipeline Execution: **Figure A8** shows the completed Basic QC pipeline workflow and a fragment of the output alignment result.


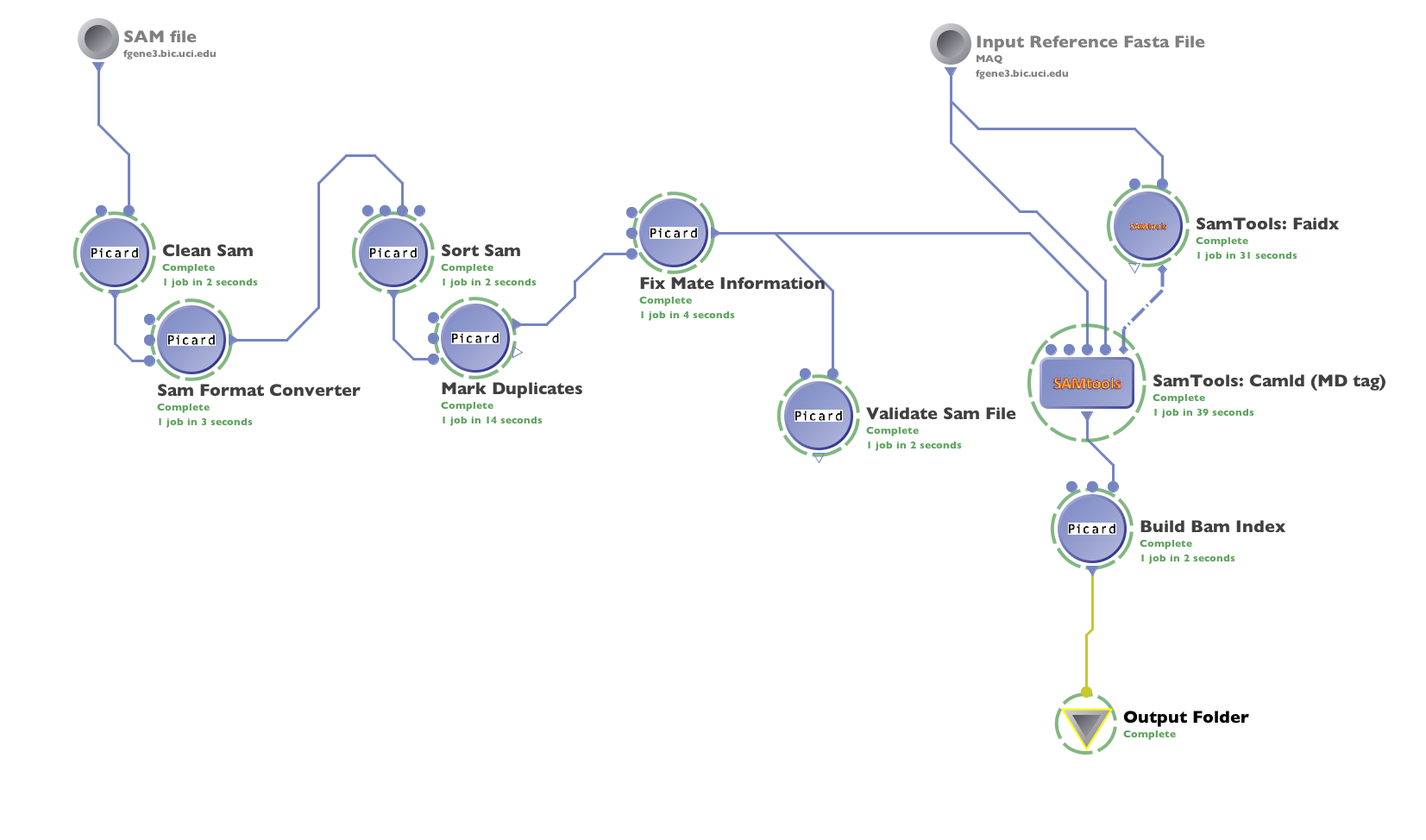


**Figure A8**: A snapshot of the completed Basic QC Pipeline workflow.

- - - Output: a properly formatted and indexed .BAM file (i.e. the compressed binary version of the Sequence Alignment/Map (SAM) format, a compact and structured representation of nucleotide sequence alignments (see **Table S1**).

#### (1.4) Advanced QC modules & Visualization

- - **URL:** GATK (http://www.broadinstitute.org/gsa/wiki/index.php/The_Genome_Analysis_Toolkit; http://www.broadinstitute.org/gsa/wiki/index.php/Downloading_the_GATK); PICARD (<http://picard.sourceforge.net/>) IGV and igvtools (<http://www.broadinstitute.org/software/igv/download>);
  - **Pipeline Workflow**

*XML Metadata description*:

*Name*:

*URL*:

*Screenshots*:

- - - Input: **Figure A9** shows a snapshot of the input parameters (data-sources) for the corresponding Pipeline workflow.


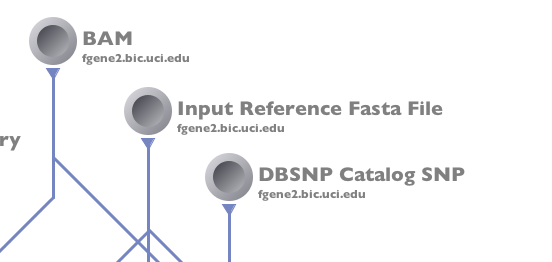


**Figure A9**: A snapshot of the input parameters (data-sources) for the Basic QC1 and QC2 Pipeline workflow.

- - - Pipeline Execution: **Figure A10** shows the completed AdvancedQC pipeline workflow.


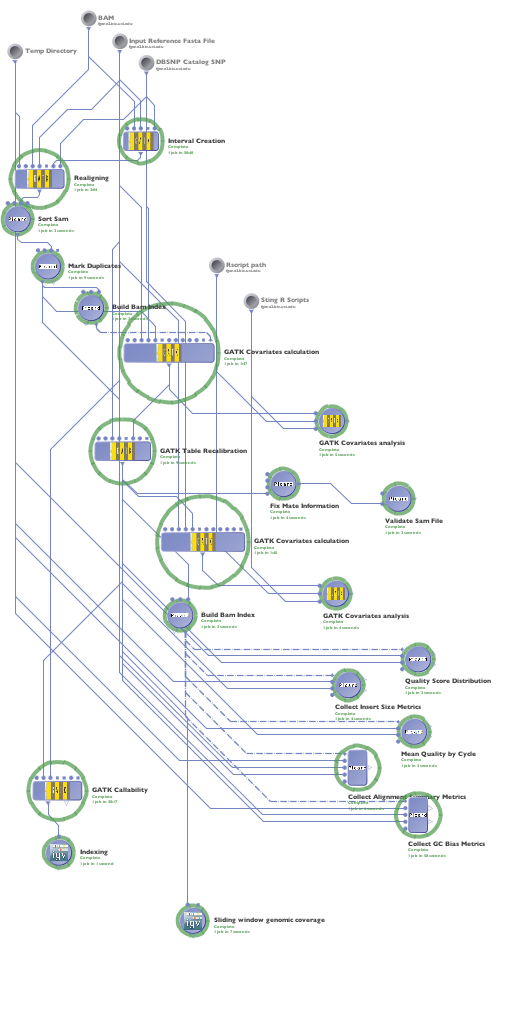


**Figure A10**: A snapshot of the completed Advanced QC Pipeline workflow.

- - - Output: a BAM file (the compressed binary version of the Sequence Alignment/Map (SAM) format, a structured representation of nucleotide sequence alignments, Table S1).

#### (2.1a) Variant Calling, Annotation and Visualization

## SAMTOOLS and ANNOVAR for comprehensive annotation: after the variants have been called with SAMTOOLS (<http://samtools.sourceforge.net/>), ANNOVAR [[70](#_ENREF_70)] allows to perform one of the most comprehensive annotation available to date.

- - **URL:** SAMTOOLS (<http://samtools.sourceforge.net/>); ANNOVAR (http://www.openbioinformatics.org/annovar/annovar_download.html).
  - **Pipeline Workflow**

*XML Metadata description*:

*Name*:

*URL*:

*Screenshots*:

- - - Input: **Figure A11** shows a snapshot of the input parameters (data-sources) for the corresponding Pipeline workflow.


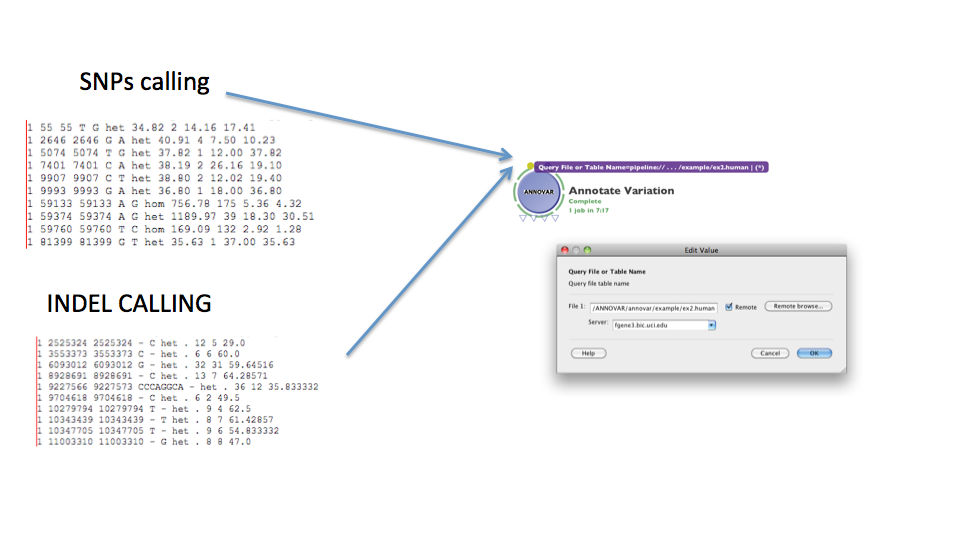


**Figure A11**: A snapshot of the input parameters for the samtools-annovar pipeline

- - - Pipeline Execution: **Figure A12** shows the completed SAMTOOLS-ANNOVAR pipeline workflow.


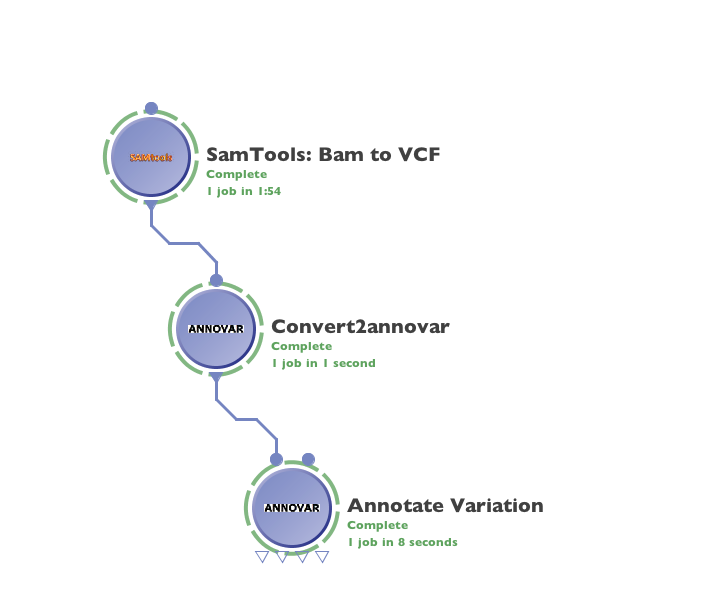


**Figure A12** A snapshot of the completed ANNOVAR Pipeline workflow (this pic might be changed with an example).

- - - Output: VCF file (see **Table S1**)

## Unified Genotyper and ANNOVAR for comprehensive annotation: after the variants have been called with the UnifiedGenotyperV2-GATK[[27](#_ENREF_27)], ANNOVAR [[70](#_ENREF_70)] performs one of the most comprehensive annotations available to date.

- **URL:** GATK (http://www.broadinstitute.org/gsa/wiki/index.php/The_Genome_Analysis_Toolkit; http://www.broadinstitute.org/gsa/wiki/index.php/Downloading_the_GATK); ANNOVAR takes only 8 minutes with these instructions: http://www.openbioinformatics.org/annovar/annovar_download.html.
  - **Pipeline Workflow**

*XML Metadata description*:

*Name*:

*URL*:

*Screenshots*:

- - - Input: **Figure A13** shows a snapshot of the input parameters (data-sources) for the corresponding Pipeline workflow.


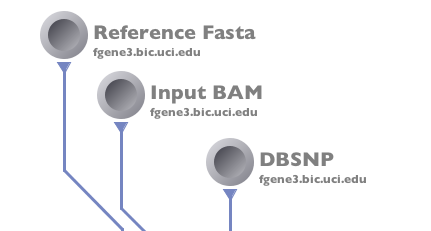


**Figure A13**: A snapshot of the input parameters for the UnifiedGenotyperV2-ANNOVAR pipeline

- - - Pipeline Execution: **Figure A14** shows the completed UnifiedGenotyper-ANNOVAR workflow.


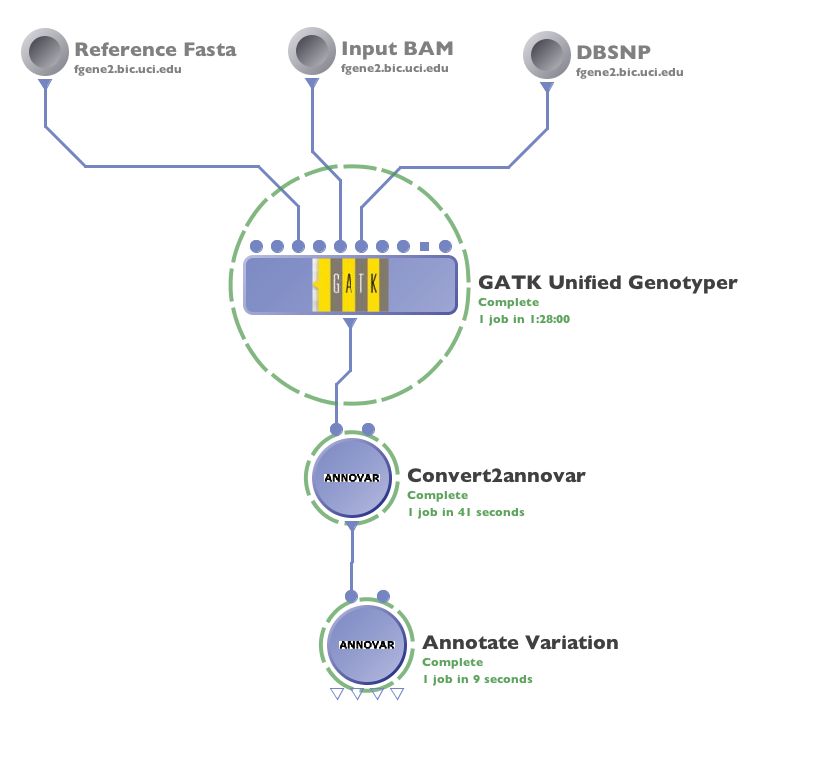


**Figure A14** A snapshot of the completed UnifiedGenotyperV2-ANNOVAR Pipeline.

- - - Output: VCF file (see **Table S1**)

## Sequence Variant Analyzer v1.0, for hg18 annotations: after the variants have been called with SAMTOOLS (<http://samtools.sourceforge.net/>) and ‘‘Estimation by Read Depth with Single Nucleotide Variants’’ (ERDS) software v1.02 (<http://web.duke.edu/~mz34/erds.htm>), they undergo annotation and visualization through Sequence Variant Analyzer (SVA, <http://www.svaproject.org>)[[71](#_ENREF_71)].

- **URL:** http://www.svaproject.org
  - **Pipeline Workflow**

*XML Metadata description*:

*Name*:

*URL*:

*Screenshots*:

- - - Input: **Figure A15** shows a snapshot of the input parameters (data-sources) for the corresponding Pipeline workflow.


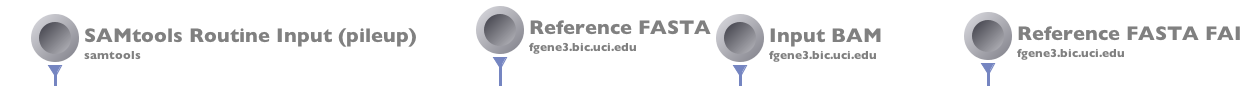


**Figure A15**: A snapshot of the input parameters for the SVA pipeline

- - - Pipeline Execution: **Figure A16** shows the completed SVA pipeline workflow.


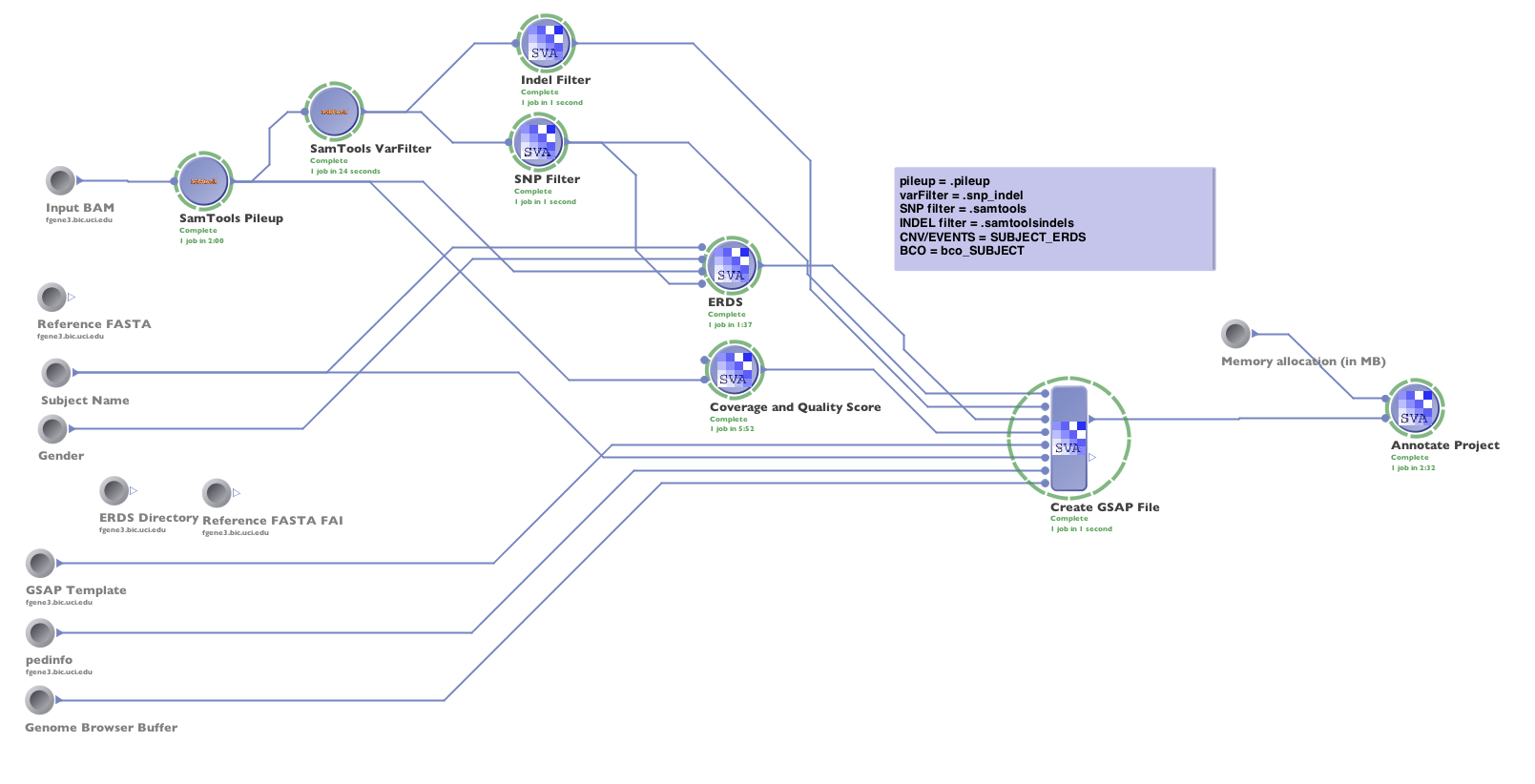


**Figure A16** A snapshot of the completed SVA Pipeline workflow (this pic might be changed with an example).

- - Output: GSAP file and project data that can be visualized in SVA

## Sequence Variant Analyzer v1.1, for hg19 annotations: This pipeline is currently under development,, pending the complete release of Sequence Variant Analyzer v1.1 (SVA, <http://www.svaproject.org>[[71](#_ENREF_71)]. Similarly to the v1.0 pipeline, the new release will allow more flexible sequence annotation, in which the user may provide custom annotation tracks in GFF3 or BED format for region-based annotation data.

#### (2.1b) CNV analysis modules

- **CNVer**
  - **URL**: <http://compbio.cs.toronto.edu/CNVer/>
  - **Description**: CNVer [[48](#_ENREF_48)] is a method for CNV detection that supplements the depth-of-coverage with paired-end mapping information, where matepairs mapping discordantly to the reference serve to indicate the presence of variation. CNVer combines this information within a unified computational framework called the donor graph, allowing it to better mitigate the sequencing biases that cause uneven local coverage. CNVer can also reconstruct the absolute copy counts of segments of the donor genome, and work with low coverage datasets. Note that we embedded the alignment step requiring BOWTIE within this module, as this process requires specific BOWTIE parameters be set for the output to be compatible with CNVer (see the CNVer README file).
  - **Pipeline Workflow**

*XML Metadata description*:

*Name*:

*URL*:

*Screenshots*:

- - - Input: **Figure A17** shows a snapshot of the input parameters (data-sources) for the corresponding Pipeline workflow.


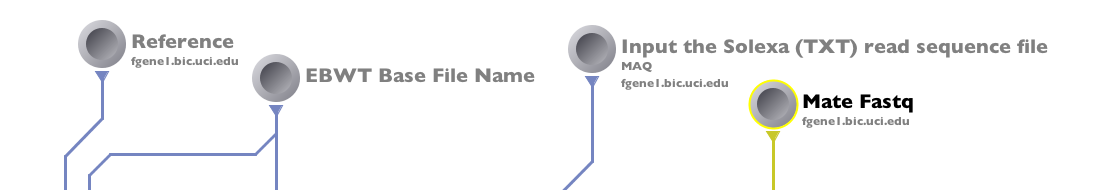


**Figure A17**: A snapshot of the input parameters (data-sources) for the CNVer Pipeline workflow.

- - - Pipeline Execution: **Figure A18** shows the completed CNVer pipeline workflow and a fragment of the output result.

**
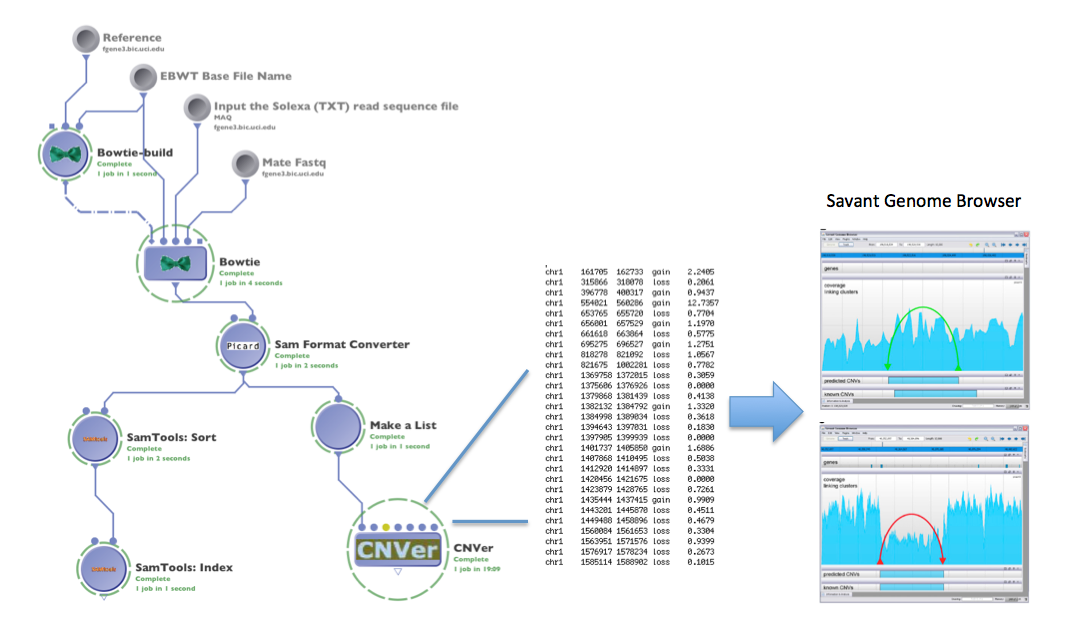
**

**Figure A18**: A snapshot of the completed CNVer Pipeline workflow. The inset image illustrates the final output result with visualization in the SAVANT genome browser.

- **CNVseq**
  - **URL**: <http://tiger.dbs.nus.edu.sg/CNV-seq/>
  - **Description**: CNVseq [[99](#_ENREF_99)] is a method for detecting DNA copy number variation (CNV) using high-throughput sequencing.  As an input program requires an output of reads aligner (e.g BOWTIE). As an output user gets CNV predictions.
  - **Installation instructions**: http://tiger.dbs.nus.edu.sg/CNV-seq/
  - **Pipeline Workflow**

*XML Metadata description*:

*Name*:

*URL*:

*Screenshots*:

- - - Input: **Figure A19** shows a snapshot of the input parameters (data-sources) for the corresponding Pipeline workflow.


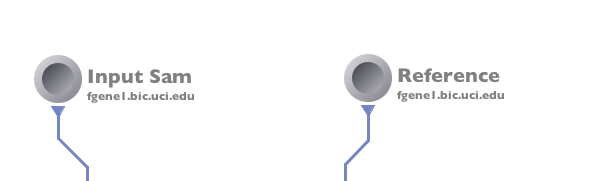


**Figure A19**: A snapshot of the input parameters (data-sources) for the CNVseq Pipeline workflow.

- - - Pipeline Execution: **Figure A20** shows the completed CNVseq pipeline workflow and a fragment of the output result.


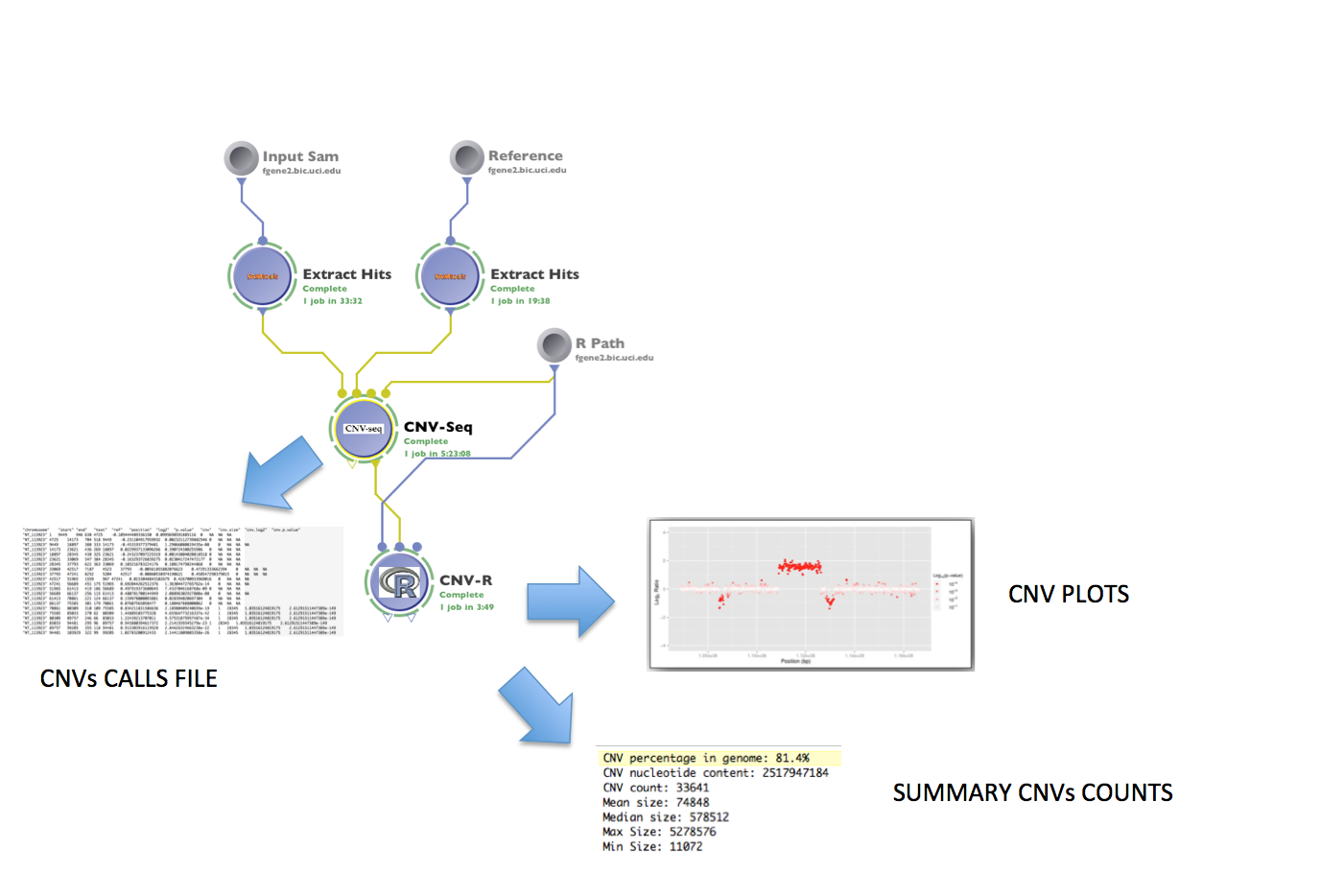


**Figure A20**: A snapshot of the completed CNVseq Pipeline workflow. The inset image illustrates the final output result: the CNV calls file, the plots (whole genome, by chromosome or by region) and the general CNV counts.

- - - Output: tabular text files with CNV counts, summary counts and PDF plots.
- **ERDS1.02/SVA1.0**
  - **URL**: <http://www.duke.edu/~mz34/erds.htm> [**http://www.svaproject.org**](http://www.svaproject.org)
  - **Description**: ERDS [[100](#_ENREF_100)] is a free, open-source software, designed for detection of copy number variants (CNVs) on human genomes from next generation sequence data. It uses paired Hidden Markov Models (PHMM) based on the expected read depth distribution of short reads and the presence of heterozygous sites. Note that this version of ERDS is compatible with SVA v1.0, but not v1.1.
  - **Pipeline Workflow**
    - Input: SAMTOOLS variant call (pileup) file, hg18 reference genome FASTA file and FASTA index, genome subject gender and pedigree information (if available) (this is a subset of the Sequence Variant Analyzer v1.0 pipeline)
    - Output: .events file, also needed as input for SVA. The annotated variants may be extracted as CSV files through the SVA GUI.
