# Supplementary material for: Next Generation Sequence Analysis and Computational Genomics Using Graphical Pipeline Workflows
Source: Genes (Basel). 2012 Aug 30;3(3):545–75. doi: 10.3390/genes3030545 (PMC3490498; doi:10.3390/genes3030545)
Supplement: Supplementary File 1 — ZIP-Document (ZIP, 7447 KB) [file genes-03-00545-s001.zip › Supplementary/SuppMat_S2.docx]

Graphical Pipeline for Computational Genomics (GPCG): User Guide 6

DATA PREPROCESSING 6

EXTRACTION OF SUBPOOL OF READS FOR TESTING 6

MODULE DESCRIPTION 6

preprocessing step (= SubSequence Extractor) 6

FORMATTING MODULES 7

MODULE DESCRIPTION 7

Conversion of solexa fastq in sanger fastq format 7

Conversion of fastq in a binary fastq file (bfq) to be used in MAQ 7

Conversion of the reference genome (fasta format) in binary fasta (bfa) to be used in MAQ 8

SOLEXA2FASTQ conversion 9

FQ2BFQ conversion 9

FASTA2BFA conversion 10

SIMULATED DATA GENERATION 11

MODULE DESCRIPTION 11

1.Production of simulated reads 11

(1) Alignment (production of a raw .SAM file) and *de novo* assembly 13

GENERAL GLOSSARY and MODULE OVERVIEW 13

(1A) Alignment and production of a raw .SAM file 16

SINGLE END ALIGNMENT 16

MAQ 16

MODULE DESCRIPTION 16

ALIGNMENT 16

1.Conversion of the reference genome (fasta format) in binary fasta 16

2.Alignment to a reference genome 16

3.MAP2SAM conversion 17

BWA_SE 18

MODULE DESCRIPTION 18

ALIGNMENT 18

1.Indexing the reference file 18

2.Read file alignment 19

3.sam file production 19

BWA_SW 21

MODULE DESCRIPTION 21

ALIGNMENT 21

1.Indexing the reference file 21

2.Solexa2fastq conversion: conversion of solexa fastq in sanger fastq format. 21

3.Read file alignment 22

PERM 23

MODULE DESCRIPTION 23

ALIGNMENT 23

1.Alignment 23

BOWTIE 25

MODULE DESCRIPTION 25

ALIGNMENT 25

1. Build bowtie index for the reference genome 25

2.Solexa2fastq conversion: conversion of solexa fastq in sanger fastq format*. 25

3.Bowtie alignment with SAM production 26

SOAP2 28

MODULE DESCRIPTION 28

ALIGNMENT 28

1.Format reference sequence: 28

2.Alignment: 28

3.SOAP to SAM conversion 29

MOSAIK 30

MODULE DESCRIPTION 30

ALIGNMENT 31

1.Build reference sequence: 31

2.Create a jumping library of the reference genome 31

3.Build reads 31

4.Alignment 32

5.SAM conversion 32

NOVOALIGN 34

MODULE DESCRIPTION 34

ALIGNMENT 34

1.Build the indexed reference sequence: 34

2.Run Novoalign for Single reads 34

PAIRED END ALIGNMENT 36

MAQ 36

MODULE DESCRIPTION 36

ALIGNMENT 36

1.Conversion of the reference genome (fasta format) in binary fasta 36

2.Alignment to a reference genome 36

3.MAP2SAM conversion 37

BWA_PE 38

MODULE DESCRIPTION 38

ALIGNMENT 38

1.Indexing the reference file 38

2.Forward read file alignment 39

3.Reverse read file alignment 39

4.Combined .sam file production 40

PERM 42

MODULE DESCRIPTION 42

ALIGNMENT 42

1.Alignment 42

BOWTIE 44

MODULE DESCRIPTION 44

ALIGNMENT 44

1.Build bowtie index for the reference genome 44

2.Solexa2fastq conversion: conversion of solexa fastq in sanger fastq format 44

3.Bowtie alignment with SAM production 45

SOAP2 47

MODULE DESCRIPTION 47

ALIGNMENT 47

1.Format reference sequence: 47

2.Alignment: 47

3.SOAP to SAM conversion 48

MOSAIK 49

MODULE DESCRIPTION 49

ALIGNMENT 49

1.Build reference sequence: 49

2. Create a jumping library of the reference genome 50

3.Build paired end reads 50

4.Alignment 51

5.SAM conversion 51

NOVOALIGN 52

MODULE DESCRIPTION 52

ALIGNMENT 52

1.Build the indexed reference sequence: 52

2.Run Novoalign for Single reads 53

(1b) De novo assembly 55

SINGLE END 55

SOAP de novo 55

MODULE DESCRIPTION 55

1.De novo assembly process 55

2.Command usage: 55

MODULE DESCRIPTION 57

1.Input preparation: conversion fastq2fasta single end reads 57

2. VELVETH: creation of the hash 58

3. VELVETG: assembly (Bruijn graph building) 58

ABYSS 60

1.Input preparation: conversion fastq2fasta single end reads 60

2.De novo assembly 60

PAIRED END 62

SOAP de novo 62

MODULE DESCRIPTION 62

1.De novo assembly process 62

2.Command usage: 62

VELVET 64

MODULE DESCRIPTION 64

1.Input preparation: conversion fastq2fasta forward reads 64

2.Input preparation: conversion fastq2fasta reverse reads 65

3. VELVET INPUT PREPARATION 65

4. VELVETH: creation of the hash 66

5. VELVETG: assembly (Bruijn graph building) 66

ABYSS 68

MODULE DESCRIPTION 68

1.Input preparation: conversion fastq2fasta forward reads 68

2.Input preparation: conversion fastq2fasta reverse reads 68

3.De novo assembly 69

(2) BASIC QC and formatting of BAM files 71

GLOSSARY and MODULE OVERVIEW 71

MODULE DESCRIPTION 74

1. Cleaning the SAM 74

2. SAM to BAM conversion 74

3. Sort .bam 75

4. Mark duplicates OR Remove duplicates 75

5. Fix Mates 75

6. Preprocess-VALIDATION of the BAM 76

7. Index the reference genome 76

8. MD tag 77

9. Indexing the .bam file 77

(3) ADVANCED data QC and cleaning of BAM files 79

GENERAL GLOSSARY and MODULE OVERVIEW 79

MODULE DESCRIPTION 82

1 Local Realignment Around Indels 82

1.1 Interval creation 82

1.2 Realignment 83

1.3 Indexing of the BAM file 83

1.4 Mark duplicates 84

2 Base Quality recalibration 84

2.1 Covariates calculation 84

2.2 Covariates analysis: plotting score parameters before recalibration 85

2.3 Recalibration 85

2.4 Post-processing BAM validation 86

2.5 Covariates calculation after recalibration 86

2.6 Covariates analysis: plotting score parameters after recalibration 87

2.7 BAM Indexing 87

3 Basic stats on reads and alignment (PICARD) 88

4 Visualization Tracks production 89

4.1 “Callability” track production for IGV 89

4.1.1 Conversion of the reference fasta file to BED 89

4.1.2 BED indexing 90

4.2 Sliding window genomic coverage 90

4.3 Visualization IGV 91

4.3.1 Creation of an IGV project 91

(4) VARIANT CALLING AND ANNOTATION 93

Glossary and Module Overview 93

Sequence Variant Analyzer v1.0, for hg18 annotations 96

1. PILEUP 96

2. VARIANTS 96

3. SNV 97

4. INDEL 97

5. EVENTS (CNV analysis) 97

6. BCO (genome coverage calculation) 98

7. Create GSAP file 98

8. Annotate project 98

SAMTOOLS and ANNOVAR for comprehensive annotation 100

(Pre-pipeline execution) Download annotations and prepare reference databases 100

ANNOTATION 101

1. Call variants with SAMTOOLS in VCF format 101

2. Convert VCF to ANNOVAR format 101

3. ANNOTATE 102

UnifiedGenotyperV2 and ANNOVAR FOR COMPREHENSIVE ANNOTATION 104

1. Variant calling with GATK 104

(5) CNVs calling modules 108

GLOSSARY and MODULE OVERVIEW 108

MODULE DESCRIPTION 111

(1) ERDS/SVA path (DOC) 111

2. BOWTIE/CNVer/SAVANT path (DOC+PEM) 111

2.1 BOWTIE alignment 111

2.1.1 Build bowtie index for the reference genome 111

2.1.2 Bowtie alignment with SAM production 112

2.1.3 SAM2BAM conversion 112

2.2 CNVer 113

2.2.1 CNVer call 113

2.2.2 Sort .bam 113

2.2.3 Indexing the .bam file 114

2.2.4 Visualization 114

3. CNVseq path 116

3.1 Hits file production 116

3.2 CNV call with CNVseq 117

# Graphical Pipeline for Computational Genomics (GPCG): User Guide

# DATA PREPROCESSING

# EXTRACTION OF SUBPOOL OF READS FOR TESTING

## MODULE DESCRIPTION

**GOAL**: extract a subset of reads to technically test the pipelines before running real data

**FINAL OUTPUT**: subset of reads in FASTQ format

### preprocessing step (= SubSequence Extractor)

Extracting a sub-group of reads from the input file. This step is not required, but may be useful for some preliminary tests and protocol validation. It restricts the size of the sequences and expedites the computation.

Input: reads files output from Illumina sequencing pipeline in solexa/fastq format (sequence.txt files/fastq files)

Label: Illumina reads sequence.txt/fastq file

Tool: LONI Sub-Sequence extractor

Server Location: /projects1/idinov/projects/Pipeline_genomics_informatics_2011/scripts/extract_lines_from_Textfile.sh

Output: Shorter sequence.txt/fastq file

Example: /projects1/idinov/projects/Pipeline_genomics_informatics_2011/scripts/extract_lines_from_Textfile.sh 1 1000 /projects1/idinov/projects/Pipeline_genomics_informatics_2011/test_data_2011/s_1_1_sequence.txt /projects/pipelineCache/pipeline/2011January27_15h51m34s061ms/SubSequenceExtractor_1.OutputTXTfile-1.txt

Pipeline Module:


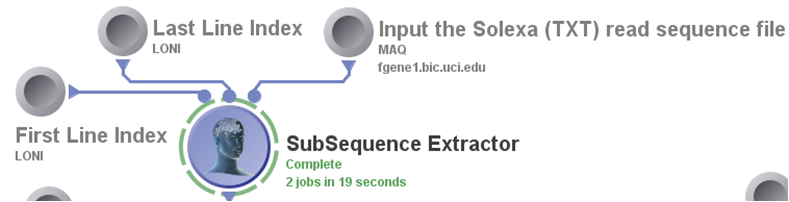


# FORMATTING MODULES

## MODULE DESCRIPTION

**GOAL**: simple format conversion to allow backward compatibility with data in solexa format.

**FINAL OUTPUT**: subset of reads in FASTQ format

### Conversion of solexa fastq in sanger fastq format

Input can be the whole sequence.txt file from Illumina Pipeline or coming from the pre-processing step [Shorter sequence.txt] file.

Input description: reads files output from Illumina sequencing pipeline in solexa format (sequence.txt files)

Label: Illumina reads sequence.txt file/ Shorter sequence.txt file

Tool: MAQ (sol2sanger option)

Server Location: /applications/maq

Output: sequence.fastq file

Example: /applications/maq/maq sol2sanger /projects/pipelineCache/pipeline/2011January27_15h51m34s061ms/SubSequenceExtractor_1.OutputTXTfile-1.txt /projects/pipelineCache/pipeline/2011January27_15h51m34s061ms/MAQSol2SangerConverter_1.Outputfastqfie-1.fastq

Pipeline Module:
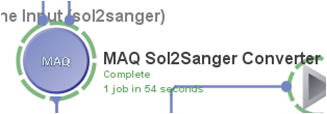


### Conversion of fastq in a binary fastq file (bfq) to be used in MAQ

Input: sequence.fastq file

Tool: MAQ (fastq2bfq option)

Server Location: /applications/maq

Output: sequence.bfq file

Example: /applications/maq/maq fastq2bfq /projects/pipelineCache/pipeline/2011January27_15h51m34s061ms/MAQSol2SangerConverter_1.Outputfastqfie-1.fastq /projects/pipelineCache/pipeline/2011January27_15h51m34s061ms/MAQFastq2BfdConverter_1.Outputbinaryfastqfilebfq-1.bfq

Pipeline Module:
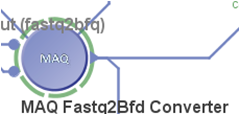


### Conversion of the reference genome (fasta format) in binary fasta (bfa) to be used in MAQ

Input: reference.fasta file (to perform the alignment)

Tool: MAQ (fasta2bfa option)

Server Location: /applications/maq

Output: reference.bfa file

Example: /applications/maq/maq fasta2bfa /projects1/idinov/projects/Pipeline_genomics_informatics_2011/test_data_2011/ref_chr2.fasta /projects/pipelineCache/pipeline/2011January27_15h51m34s061ms/MAQFasta2BfaConverter_1.Outputbinaryfastafilebfa-1.bfa

Pipeline Module:
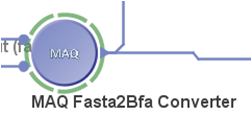


**FINAL MODULE APPEARANCE**


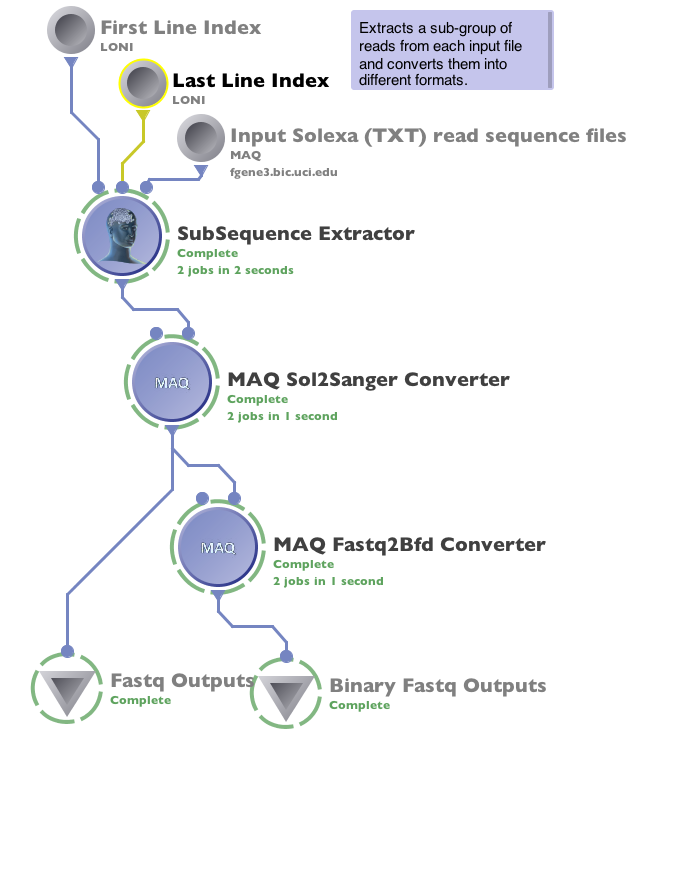


**Figure 1**: A snapshot of the completed Subsequence Extractor module.

As the users may need to use these formatting steps separately, we embedded them also in individual pipelines ready to go, like described in the following sections.

# SOLEXA2FASTQ conversion

Input description: reads files output from Illumina sequencing pipeline in solexa format (sequence.txt files)

Label: Illumina reads sequence.txt file/ Shorter sequence.txt file

Tool: MAQ (sol2sanger option)

Server Location: /applications/maq

Output: sequence.fastq file

Example: /applications/maq/maq sol2sanger /projects/pipelineCache/pipeline/2011January27_15h51m34s061ms/SubSequenceExtractor_1.OutputTXTfile-1.txt /projects/pipelineCache/pipeline/2011January27_15h51m34s061ms/MAQSol2SangerConverter_1.Outputfastqfie-1.fastq

Pipeline Module:
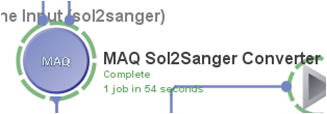


# FQ2BFQ conversion

Input: sequence.fastq file

Tool: MAQ (fastq2bfq option)

Server Location: /applications/maq

Output: sequence.bfq file

Example: /applications/maq/maq fastq2bfq /projects/pipelineCache/pipeline/2011January27_15h51m34s061ms/MAQSol2SangerConverter_1.Outputfastqfie-1.fastq /projects/pipelineCache/pipeline/2011January27_15h51m34s061ms/MAQFastq2BfdConverter_1.Outputbinaryfastqfilebfq-1.bfq

Pipeline Module:
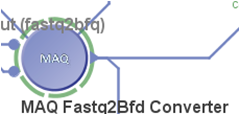


# FASTA2BFA conversion

Input: reference.fasta file (to perform the alignment)

Tool: MAQ (fasta2bfa option)

Server Location: /applications/maq

Output: reference.bfa file

Example: /applications/maq/maq fasta2bfa /projects1/idinov/projects/Pipeline_genomics_informatics_2011/test_data_2011/ref_chr2.fasta /projects/pipelineCache/pipeline/2011January27_15h51m34s061ms/MAQFasta2BfaConverter_1.Outputbinaryfastafilebfa-1.bfa

Pipeline Module:
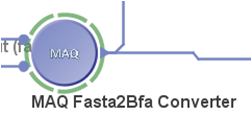


# SIMULATED DATA GENERATION

# MODULE DESCRIPTION

**GOAL**: generate simulation data

**FINAL OUTPUT**: forward and reverse reads file to be used with the aligners, a file formatted for BFAST and a mutation file.

### 1.Production of simulated reads

Input: in.ref.fa

Tool: dwgsim

Server Location: /applications/dwgsim-0.1.5

Output: test.prefix.bwa.read1.fastq, test.prefix.bwa.read2.fastq, test.prefix.bfast.fastq, test.prefix.mutations.txt

Example: dwgsim /projects1/Reference_genomes/hg18_ensembl/gatk-canonical/gatk-hg18_ensembl.fa -N 15000000 -1 100 -2 100 /projects2/USC/canonical-test-data/dwgsim_data/100bp_15millions.prefix

Pipeline Module:


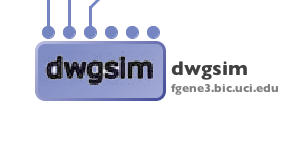


MAIN PARAMETERS:

Usage: dwgsim [options] <in.ref.fa> <out.prefix>

Options:

-e FLOAT base/color error rate of the first read [from 0.020 to 0.020 by 0.000]

-E FLOAT base/color error rate of the second read [from 0.020 to 0.020 by 0.000]

-d INT inner distance between the two ends [500]

-s INT standard deviation [0.000]

-N INT number of read pairs [1000000]

-1 INT length of the first read [70]

-2 INT length of the second read [70]

-r FLOAT rate of mutations [0.0010]

-R FLOAT fraction of mutations that are indels [0.10]

-X FLOAT probability an indel is extended [0.30]

-y FLOAT probability of a random DNA read [0.05]

-n INT maximum number of Ns allowed in a given read [0]

-c INT generate reads for [0]:

0: Illumina

1: SOLiD

2: Ion Torrent

-S INT generate reads [0]:

0: default (opposite strand for Illumina, same strand for SOLiD/Ion Torrent)

1: same strand (mate pair)

2: opposite strand (paired end)

-f STRING the flow order for Ion Torrent data [(null)]

-H haploid mode [False]

-z INT random seed (-1 uses the current time) [-1]

-m FILE the mutations txt file to re-create [not using]

-b FILE the bed-like set of candidate mutations [not using]

-h print this message

# (1) Alignment (production of a raw .SAM file) and *de novo* assembly

# GENERAL GLOSSARY and MODULE OVERVIEW

“Input”: name of the input file

“Label”: is specified when the name of the input on the pipeline canvas has to be slightly different form the one specified in the Input section to be more clear.

“Tool”: script/program in use

“Server Location”: location on the fgene server

“Output”: name of the output


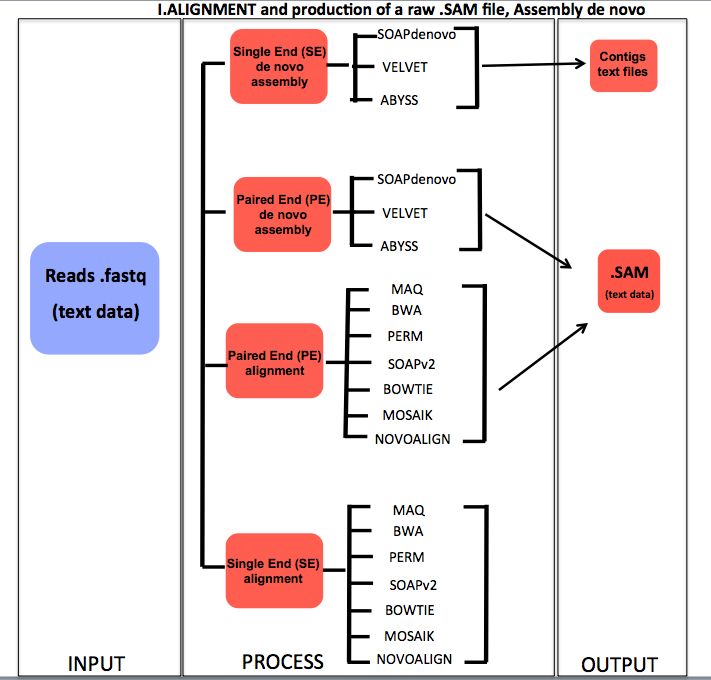


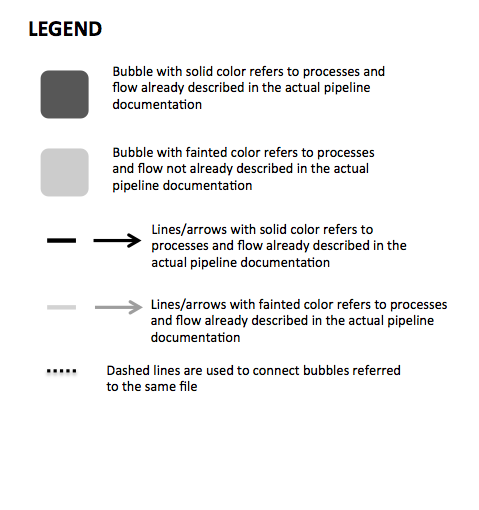


# (1.1) Alignment and production of a raw .SAM file

# SINGLE END ALIGNMENT

# MAQ

## MODULE DESCRIPTION

**GOAL**: align the reads (FASTQ format) to the reference genome with MAQ

**FINAL OUTPUT**: raw .sam file (pre-QC)

To produce the binary reads fastq files the preprocessing pipelines SOLEXA2FASTQ and FQ2BFQ can be used.

# ALIGNMENT

### 1.Conversion of the reference genome (fasta format) in binary fasta

Input: reference.fasta file (to perform the alignment)

Tool: MAQ (fasta2bfa option)

Server Location: /applications/maq

Output: reference.bfa file

Example: /applications/maq/maq fasta2bfa /projects1/idinov/projects/Pipeline_genomics_informatics_2011/test_data_2011/ref_chr2.fasta /projects/pipelineCache/pipeline/2011January27_15h51m34s061ms/MAQFasta2BfaConverter_1.Outputbinaryfastafilebfa-1.bfa

Pipeline Module:
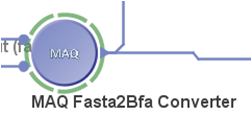


### 2.Alignment to a reference genome

Input: sequence.bfq, reference.bfa

Tool: MAQ (map option)

Server Location: /applications/maq

Output: alignment.map file

Example: /applications/maq/maq map /projects/pipelineCache/pipeline/2011January27_15h51m34s061ms/MAQMapper_1.Outputmappingfilemap-1.map /projects/pipelineCache/pipeline/2011January27_15h51m34s061ms/MAQFasta2BfaConverter_1.Outputbinaryfastafilebfa-1.bfa /projects/pipelineCache/pipeline/2011January27_15h51m34s061ms/MAQFastq2BfdConverter_1.Outputbinaryfastqfilebfq-1.bfq

Pipeline Module:
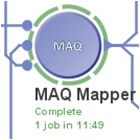


### 3.MAP2SAM conversion

Input: alignment.map file

Tool: samtools (maq2sam-long option)

Server Location: /applications/samtools-0.1.7_x86_64-linux

Output: alignment.sam file

Example:

- Script: /projects1/idinov/projects/Pipeline_genomics_informatics_2011/scripts/maq2sam-long.csh /projects/pipelineCache/pipeline/2011January27_15h51m34s061ms/MAQMapper_1.Outputmappingfilemap-1.map /projects/pipelineCache/pipeline/2011January27_15h51m34s061ms/SamToolsmaq2sam-long_1.OutputSAMfile-1.sam
- Native call: maq2sam-long /ifs/pl_cache/cranium/pipelnvr/2010December03_10h22m38s036ms/MAQMapper_1.Outputmappingfilemap-1.map > /ifs/pl_cache/cranium/pipelnvr/2010December03_10h22m38s036ms/SamToolsmaq2sam-long_2.OutputBAMfile-1.bam

Pipeline Module:
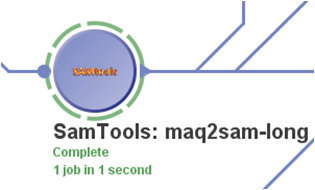


**FINAL MODULE APPEARANCE**


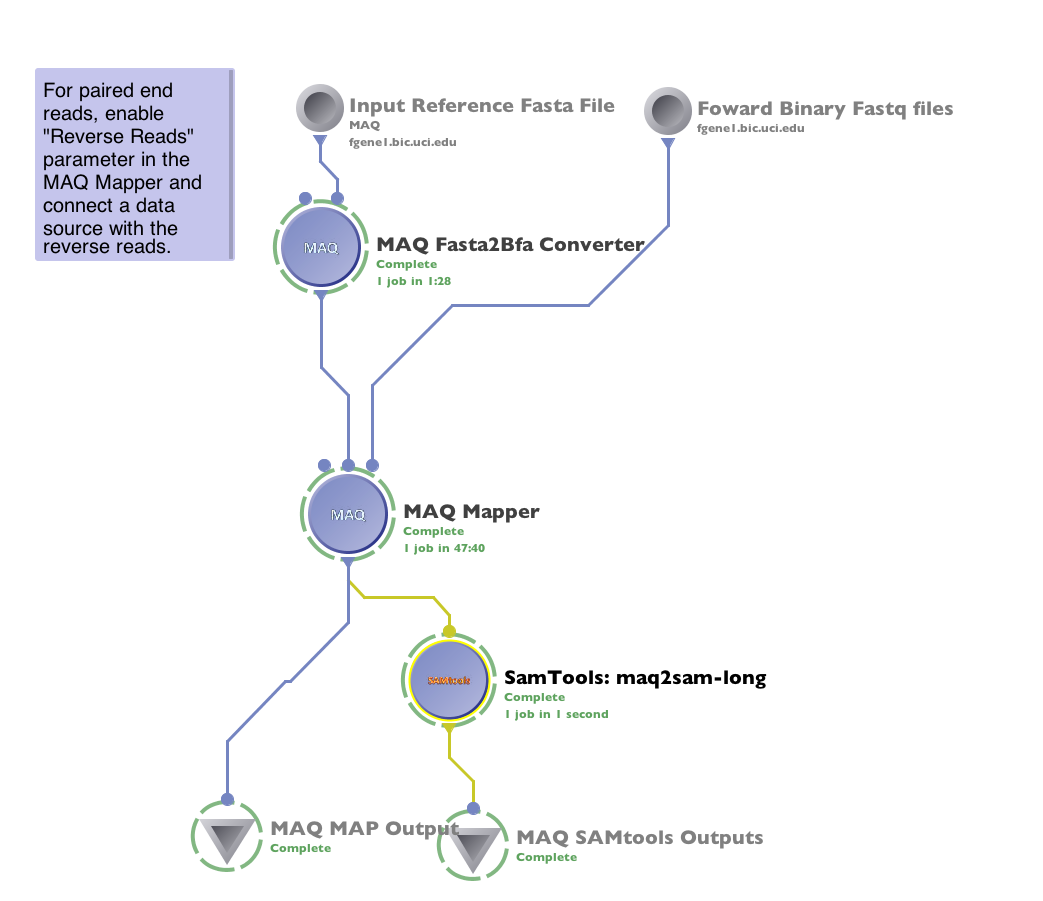


**Figure 2**: A snapshot of the completed MAQ Pipeline workflow.

# BWA_SE

## MODULE DESCRIPTION

**GOAL**: align the reads (FASTQ format) to the reference genome with BWA

**FINAL OUTPUT**: raw .sam file (pre-QC)

To produce the binary reads fastq files from reads in solexa format, the preprocessing pipeline SOLEXA2FASTQ can be used.

# ALIGNMENT

### 1.Indexing the reference file

Input: reference.fasta

Tool: bwa (index option)

Server Location: /applications/BWA/bwa-0.5.9rc1

Output: set of files that are created in the same folder of the reference.fasta file

Example: bwa index /projects1/ADNI_2/BWA/ref-hg18-ensembl/ensembl_hg18_ncbi36_r50.fa

Pipeline Module:


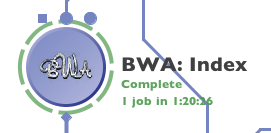


### 2.Read file alignment

Input: sequence.fastq file, reference.fasta (along with all the files created in the step I.1. The command line for the alignment points only to the reference.fasta)

Tool: bwa (aln option)

Server Location: /applications/BWA/bwa-0.5.9rc1

Output: sequence.sai

Example: bwa aln reference.fasta sequence.fastq > sequence.sai

Pipeline Module:

**
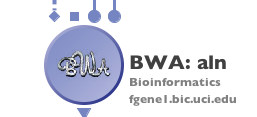
**

### 3.sam file production

Input: sequence_read1.sai, sequence_read2.sai, sequence_read1.fastq, sequence_read2.fastq

Tool: bwa (sampe option, with )

Server Location: /applications/BWA/bwa-0.5.9rc1

Output: alignment.sam

Usage: bwa samse [-n maxOcc] <in.db.fasta> <in.sai> <in.fq> > <out.sam>

MAIN OPTIONS:

-n INT Maximum number of alignments to output in the XA tag for reads paired properly. If a read has more than INT hits, the XA tag will not be written. [3]

-r STR Specify the read group in a format like ‘@RG\tID:foo\tSM:bar’. [null]

Example: bwa samse reference.fasta sequence.sai sequence.fastq -r '@RG\tID:JLK-227\tSM:JLK-227' > alignment.sam

Notes: the read group id name MUST be formatted as shown, as BWA expects it:

'@RG\tID:YOURID\tSM:YOURSM'

* The entire @RG string surrounded by single quotes (')

* YOURID and YOURSM replaced by your own IDs and SMs, no whitespaces, tabs or colons

Pipeline Module:


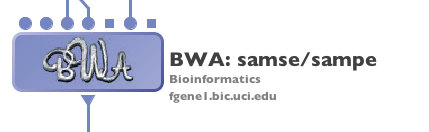


**FINAL MODULE APPEARANCE**


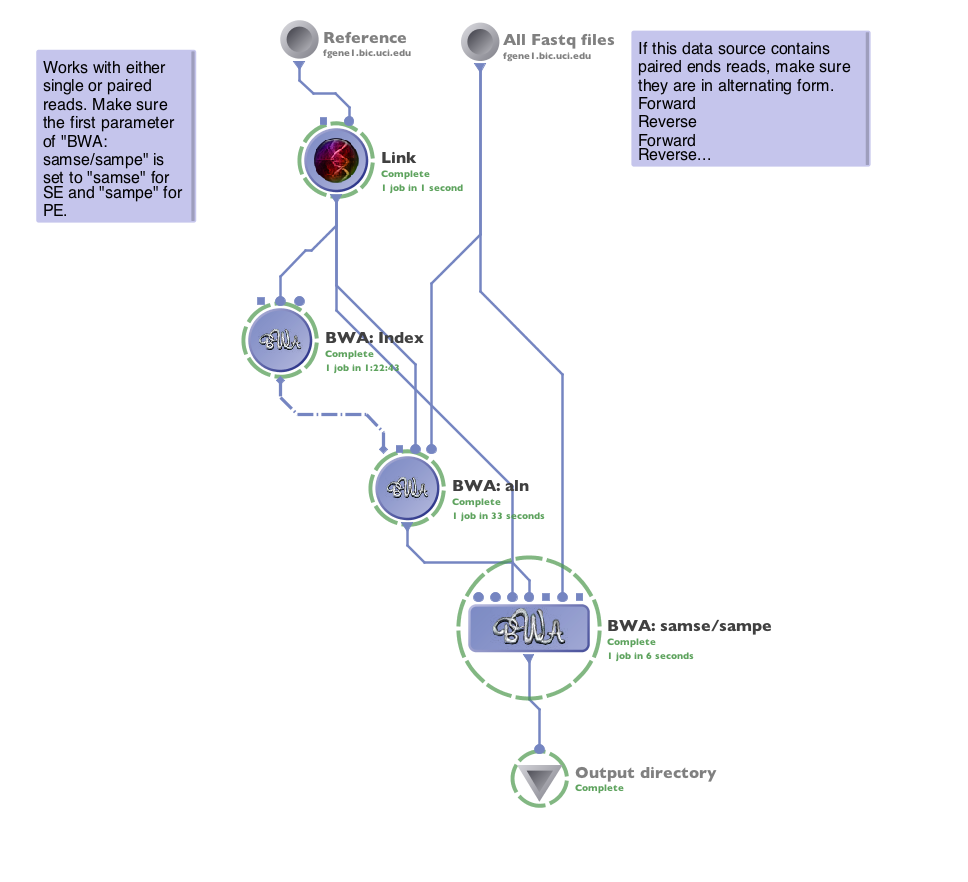


**Figure 3**: A snapshot of the completed BWA (SE and PE have the same appearance) Pipeline workflow.

# BWA_SW

## MODULE DESCRIPTION

**GOAL**: align the reads (FASTQ format) to the reference genome with BWA-SW in SE. This algorithm is designed for long reads with more errors. It performs heuristic Smith-Waterman-like alignment to find high-scoring local hits. On low-error short queries, BWA-SW is slower and less accurate than the first algorithm, but on long queries, it is better.

**FINAL OUTPUT**: raw .sam file (pre-QC)

# ALIGNMENT

### 1.Indexing the reference file

Input: reference.fasta

Tool: bwa (index option)

Server Location: /applications/BWA/bwa-0.5.9rc1

Output: set of files that are created in the same folder of the reference .fasta file

Example: bwa index /projects1/ADNI_2/BWA/ref-hg18-ensembl/ensembl_hg18_ncbi36_r50.fa

Pipeline Module:


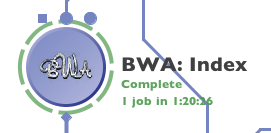


### 2.Solexa2fastq conversion: conversion of solexa fastq in sanger fastq format.

Input can be the whole sequence.txt file from Illumina Pipeline or coming from the pre-processing step [Shorter sequence.txt] file.

Input description: reads files output from Illumina sequencing pipeline in solexa format (sequence.txt files)

Label: Illumina reads sequence.txt file/ Shorter sequence.txt file

Tool: MAQ (sol2sanger option)

Server Location: /applications/maq

Output: sequence.fastq file

Example: /applications/maq/maq sol2sanger /projects/pipelineCache/pipeline/2011January27_15h51m34s061ms/SubSequenceExtractor_1.OutputTXTfile-1.txt /projects/pipelineCache/pipeline/2011January27_15h51m34s061ms/MAQSol2SangerConverter_1.Outputfastqfie-1.fastq

Pipeline Module:
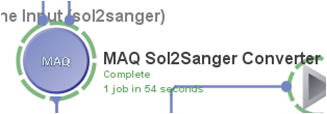


### 3.Read file alignment

Input: sequence.fastq file, reference.fasta (along with all the files created in the step I.1. The command line for the alignment points only to the reference.fasta)

Tool: bwa (bwa-sw option)

Server Location: /applications/BWA/bwa-0.5.9rc1

Output: sequence.sai

Example: bwa bwa-sw reference.fasta sequence.fastq > sequence.sai

Pipeline Module:


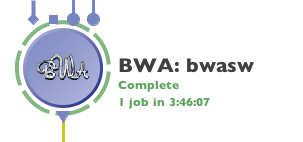


**FINAL MODULE APPEARANCE**


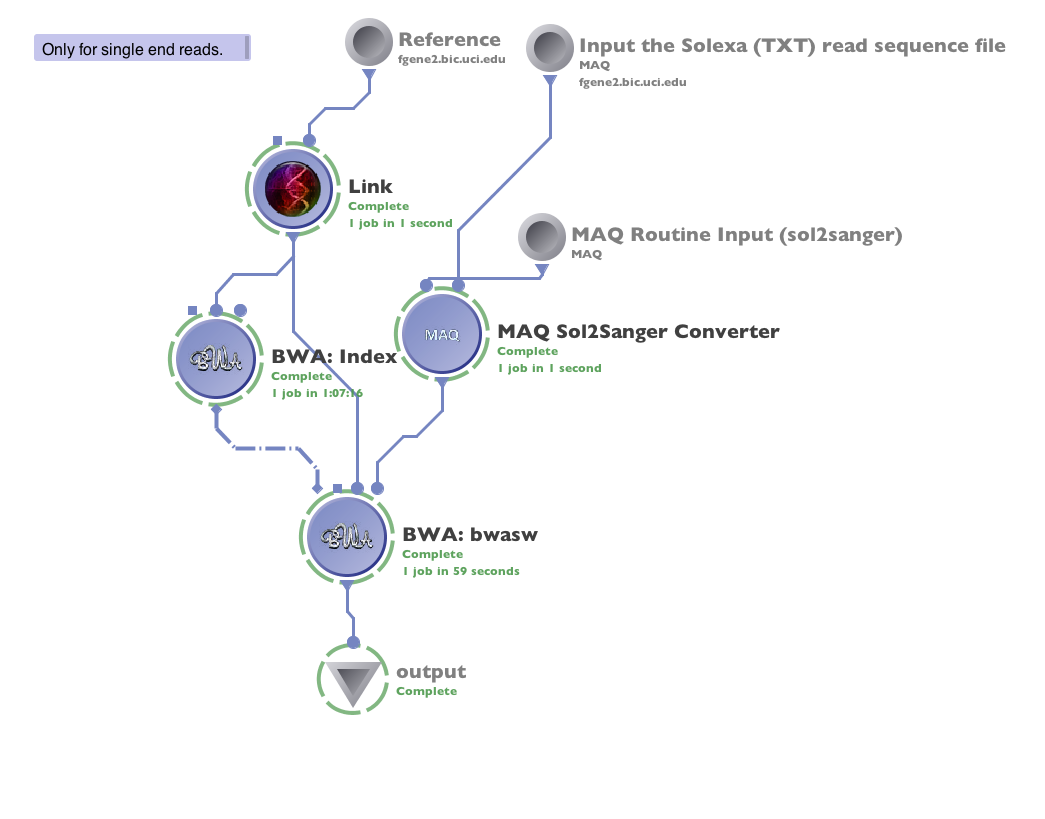


**Figure 4**: A snapshot of the completed BWA-SW Pipeline workflow.

# PERM

## MODULE DESCRIPTION

**GOAL**: align the reads (FASTQ format) to the reference genome with PERM

**FINAL OUTPUT**: raw .sam file (pre-QC)

# ALIGNMENT

### 1.Alignment

Input: sequence.fastq file, file reference.fasta

Tool: PERM (perm)

Server Location: /applications/PERM/PerM0.3.3Source

Output: alignment.sam, unmapped_reads.fastq, ambiguous_reads.fastq

Example:

nohup perm /projects/Miserv/Sequencing/Ref_genomes/human_seguence/chr6.fa -A -a /projects1/TKT_SEQ_2011/UID309_H_reads_morethan200_align.fastq -u UID309_H_unmapped_reads.fastq --log /projects1/TKT_SEQ_2011/UID309_H_perm.log /projects/Miserv/Sequencing/MAQ/Input/FEBIT_tkt/20090828.06s2.1_sequence.fastq -o /projects1/TKT_SEQ_2011/perm_UID309_H_chr6.sam > /projects1/TKT_SEQ_2011/perm_UID309_H_chr6.log &

Pipeline Module:

**
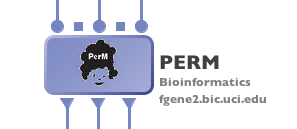
**

**FINAL MODULE APPEARANCE**

**
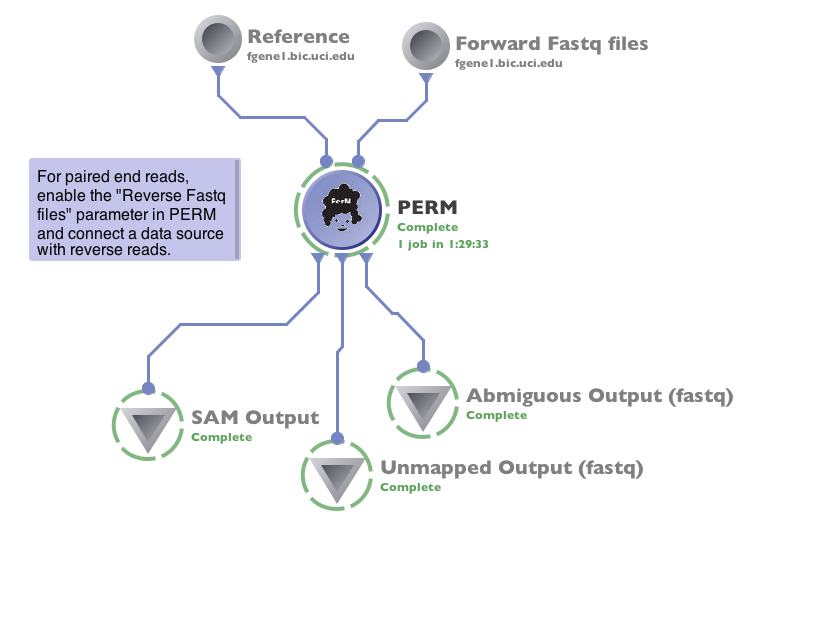
**

**Figure 5**: A snapshot of the completed PERM SE Pipeline workflow.

# BOWTIE

## MODULE DESCRIPTION

**GOAL**: align the reads (FASTQ format) to the reference genome with BOWTIE

**FINAL OUTPUT**: raw .sam file (pre-QC)

# ALIGNMENT

### 1. Build bowtie index for the reference genome

Input: ref.fa reference files (preferentially the UCSC fasta reference genome, chr1-22,X,Y. See notes if using ensemble genome)

Tool: bowtie (bowtie-build command)

Server Location: /applications/rseqtools/example_dataset/bowtie-0.12.7

Output: series of .ebwt files

Example: bowtie-build ${CD}/human_genome2.fa ucsc_hg18_new_bowtie > \

ucsc_hg18_new_bowtie.log

Pipeline Module:


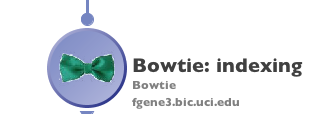


### 2.Solexa2fastq conversion: conversion of solexa fastq in sanger fastq format*.

(**note: now this step can be avoided as the recent versions of Bowtie allow using directly the solexa sequence.txt files.See the option –solexa_quals)

Input can be the whole sequence.txt file from Illumina Pipeline or coming from the pre-processing step [Shorter sequence.txt] file.

Input description: reads files output from Illumina sequencing pipeline in solexa format (sequence.txt files)

Label: Illumina reads sequence.txt file/ Shorter sequence.txt file

Tool: MAQ (sol2sanger option)

Server Location: /applications/maq

Output: sequence.fastq file

Example: /applications/maq/maq sol2sanger /projects/pipelineCache/pipeline/2011January27_15h51m34s061ms/SubSequenceExtractor_1.OutputTXTfile-1.txt /projects/pipelineCache/pipeline/2011January27_15h51m34s061ms/MAQSol2SangerConverter_1.Outputfastqfie-1.fastq

Pipeline Module:
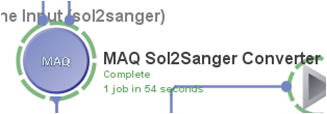


### 3.Bowtie alignment with SAM production

Input: sequence.fastq file (or sequence.txt files, see * note)

Label: Illumina reads sequence.fastq files

Tool: bowtie

Server Location: /applications/BOWTIE/bowtie-0.12.7

Output: alignment.sam

Example: bowtie ${CD}/ucsc_hg18_new_bowtie –a /projects2/USC/rnaseq/rnaseq_reads/RSEQTOOLS/RSEQTOOLS_input_files/hg18 -v 2 -1 s_1_1_sequence.150k.fastq --sam ${CD}/941408_bduc_ucsc_hg18_new.sam >

${CD}/941408_bduc_ucsc_hg18_new.log

Pipeline Module:


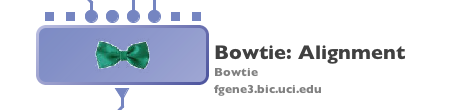


****IMPORTANT**: the flag –solexa-quals has been recently introduced. Converts input qualities from [Solexa](http://en.wikipedia.org/wiki/FASTQ_format#Variations) (which can be negative) to [Phred](http://en.wikipedia.org/wiki/FASTQ_format#Variations) (which can't). This is usually the right option for use with (unconverted) reads emitted by GA Pipeline versions prior to 1.3. Default: off.

the flag --solexa1.3-quals this is usually the right option for use with (unconverted) reads emitted by GA Pipeline version 1.3 or later. Default: off.

These options allow inputting into bowtie solexa fastq file directly (sequence.txt) without needing any conversion (avoiding the step 1.2 described in this section).

Example: /applications/rseqtools/example_dataset/bowtie-0.12.7/bowtie -a /projects2/USC/rnaseq/rnaseq_reads/RSEQTOOLS/RSEQTOOLS_input_files/hg18 -v 2 -1 s_1_1_sequence.150k.txt --solexa1.3-quals --sam test_bowtie.sam> log.log &

**FINAL MODULE APPEARANCE**


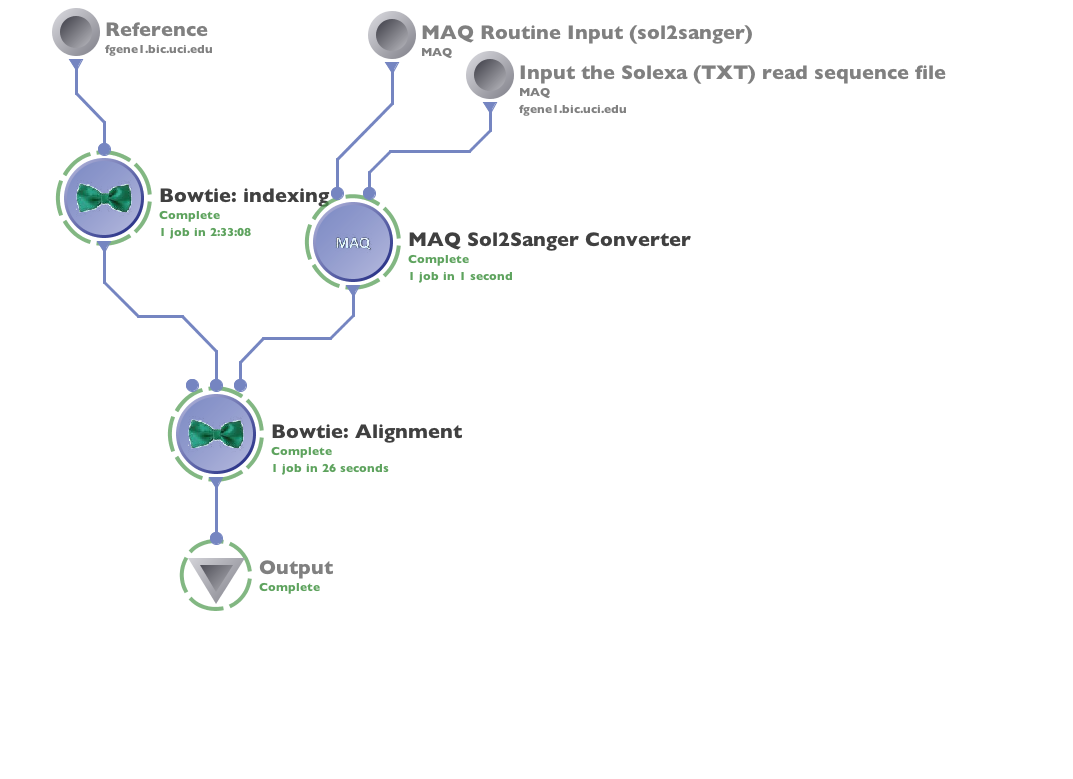


**Figure 6**: A snapshot of the completed Bowtie SE Pipeline workflow.

# SOAP2

## MODULE DESCRIPTION

**GOAL**: align the reads (FASTQ format) to the reference genome with SOAP

**FINAL OUTPUT**: raw .sam file (pre-QC)

# ALIGNMENT

### 1.Format reference sequence:

Input: reference.fasta file

Tool:SOAP2

Server Location <ExecutablePath>/2bwt-builder

Output: 13 index files, all their prefixes are your_fasta file name with “.index” added, e.g. human_genome.fa.index. The suffixes include *.amb, *.ann, *.bwt, *.fmv, *.hot, *.lkt, *.pac, *.rev.bwt, *.rev.fmv, *.rev.lkt, *.rev.pac, *.sa, and *.sai.

Example: ./2bwt-builder ~/human_genome.fa

Pipeline Module:


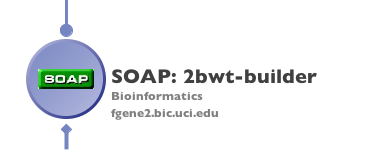


### 2.Alignment:

Input: reads.fastq

Tool: SOAP2

Server Location: ./soap

Output: alignment.soap

Example: ./soap –a <reads_a> -D <index.files> -o <output></output>

Pipeline Module:


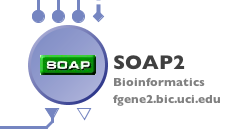


NOTE: For the –D option, the program can only accept the prefix of your index files, such as “~/human_genome.fa.index”.

MAIN OPTIONS:

-D STR Prefix name for reference index [*.index].

-a STR Query file, for SE reads alignment or one end of PE reads

-b STR Query b file, one end of PE reads

-o STR Output file for alignment results

-2 STR Output file contains mapped but unpaired reads when do PE alignment

-u STR Output file for unmapped reads, [none]

-m INT Minimal insert size INT allowed for PE, [400]

-x INT Maximal insert size INT allowed for PE, [600]

-n INT Filter low quality reads contain more INT bp Ns, [5]

-t Output reads id instead reads name, [none]

-r INT How to report repeat hits, 0=none; 1=random one; 2=all, [1]

-R RF alignment for long insert size(>= 2k bps) PE data, [none] FR alignment

-l INT For long reads with high error rate at 3'-end, those

can't align whole length, then first align 5' INT bp

subsequence as a seed, [256] use whole length of the read

-v INT Totally allowed mismatches in one read, [2]

-M INT Match mode for each read or the seed part of read, which

shouldn't contain more than 2 mismaches, [4]

0: exact match only

1: 1 mismatch match only

2: 2 mismatch match only

3: [gap] (coming soon)

4: find the best hits

-p INT Multithreads, n threads, [1]

### 3.SOAP to SAM conversion

Input: alignment.soap

Tool: soap2sam.pl

Server Location: ./soap2sam.pl (available at http://soap.genomics.org.cn/soapaligner.html)

Output: alignment.sam

Example: soap2sam.pl [-p] <aln.soap>

Pipeline Module:


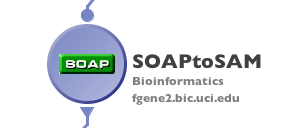


**FINAL MODULE APPEARANCE**


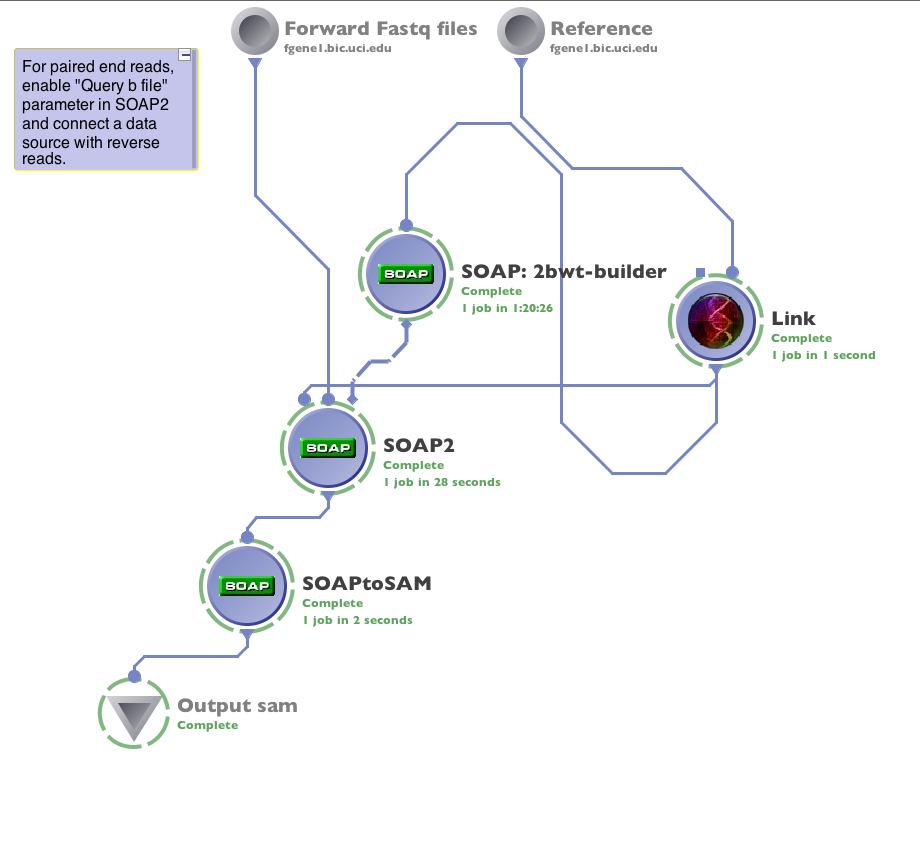


**Figure 7**: A snapshot of the completed SOAPv2 SE Pipeline workflow.

# MOSAIK

## MODULE DESCRIPTION

**GOAL**: align the reads (directly solexa FASTQ format) to the reference genome with MOSAIK

**FINAL OUTPUT**: raw .sam file (pre-QC)

# ALIGNMENT

### 1.Build reference sequence:

Input: reference.fasta file

Tool:.MosaikBuild

Server Location <ExecutablePath>/MosaikBuild

Output: reference.dat

Example: ./MosaikBuild –fr ~/human_genome.fa –oa human_genome.dat

Pipeline Module:


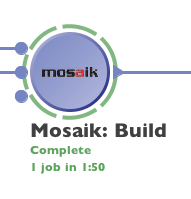


### 2.Create a jumping library of the reference genome

Input: reference.dat

Tool:.MosaikJump

Server Location <ExecutablePath>/MosaikJump

Output: reference_jumping_library

Example: ./MosaikJump –ia human_genome.dat –out human_genome_hs15 -hs 15

Pipeline Module:


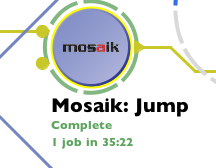


### 3.Build reads

Input: sequence.txt (reads in solexa fastq format)

Tool:.MosaikBuild

Server Location <ExecutablePath>/MosaikBuild

Output: reads.dat

Example: ./MosaikBuild –q sequence.txt –out reads.dat –st Illumina

Pipeline Module:


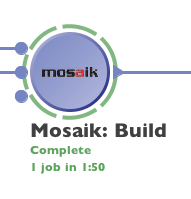


### 4.Alignment

Input: reference.dat, reads.dat, reference_jumping_library

Tool:.MosaikAligner

Server Location <ExecutablePath>/MosaikAligner

Output: reads_aligned_mosaik.dat

Example: ./MosaikAligner –in reads.dat –out reads_aligned_mosaik.dat –ia reference.dat –hs 15 –mm 2 –mhp 100 –j reference_jumping_library

Pipeline Module:


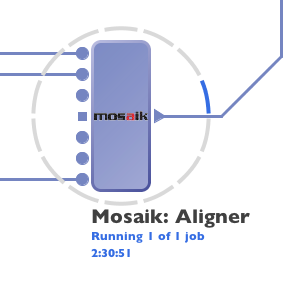


### 5.SAM conversion

Input: reads_aligned_mosaik.dat

Tool:.MosaikText

Server Location <ExecutablePath>/MosaikAligner

Output:

Example: ./MosaikText –in reads_aligned_mosaik.dat –sam reads_aligned_mosaik.sam

IMPORTANT: with the bam argument is possible to export directly in BAM

Pipeline Module:


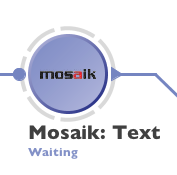


**FINAL MODULE APPEARANCE**


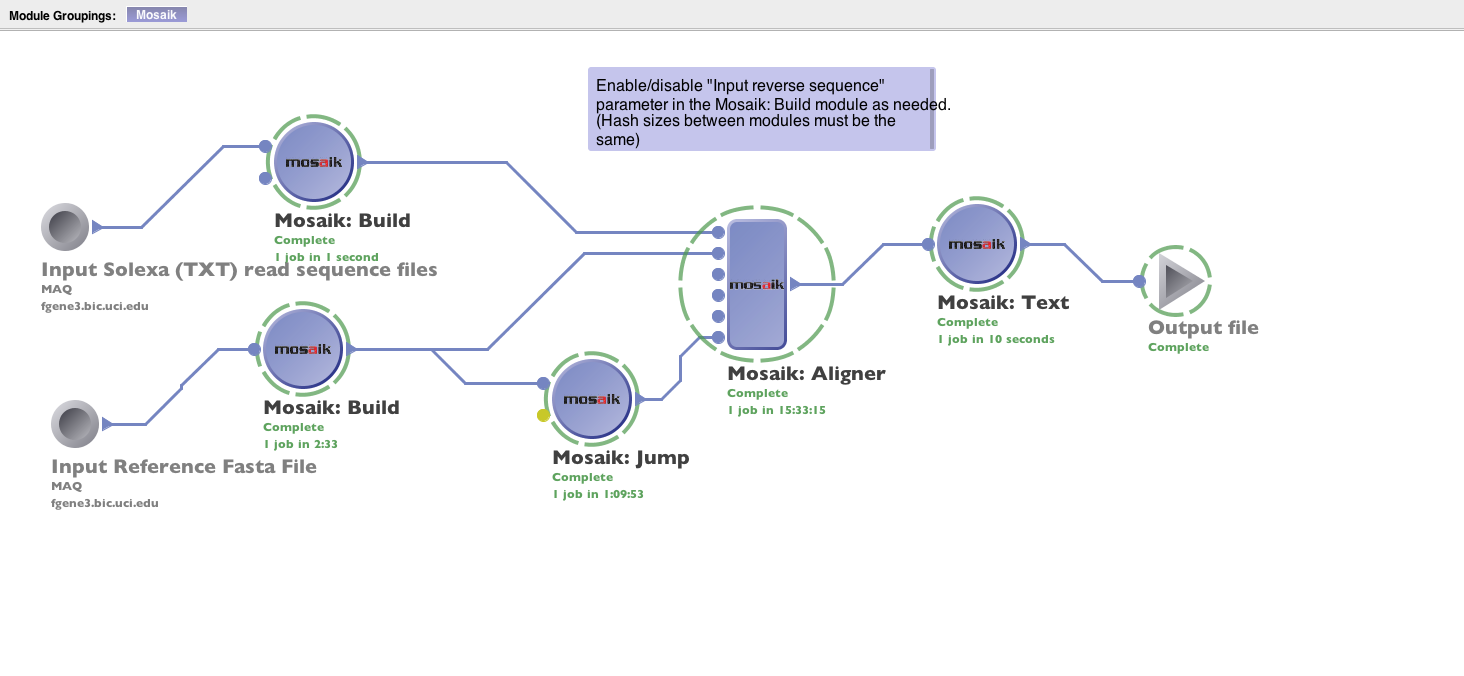


**Figure 8**: A snapshot of the completed Mosaik SE Pipeline workflow.

# NOVOALIGN

## MODULE DESCRIPTION

**GOAL**: align the reads (directly solexa or FASTQ format) to the reference genome with NOVOALIGN

**FINAL OUTPUT**: raw .sam file (pre-QC)

# ALIGNMENT

### 1.Build the indexed reference sequence:

Input: reference.fasta file

Tool:.Novoalign (novoindex.sh)

Server Location: /projects1/Alignment/Novoalign/

Output: reference.nix file

Example: ./novocraft/novoindex ssuis.nix ./sampledata/S_suis.dna

Pipeline Module:

**
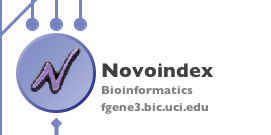
**

### 2.Run Novoalign for Single reads

Input: reference.fasta file (File formats allowed include Solexa PRB, Sanger FASTQ, FASTA, Solexa FASTQ, Illumina FASTQ, and Illumina qseq_txt.)

Tool:.Novoalign (novoindex.sh)

Server Location: /projects1/Alignment/Novoalign/

Output: reference.nix file

Example: *./novocraft/novoalign -d ssuis.nix -f ./sampledata/s_1_sequence.txt

Pipeline Module:


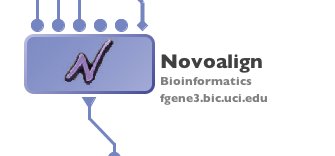


**FINAL MODULE APPEARANCE**


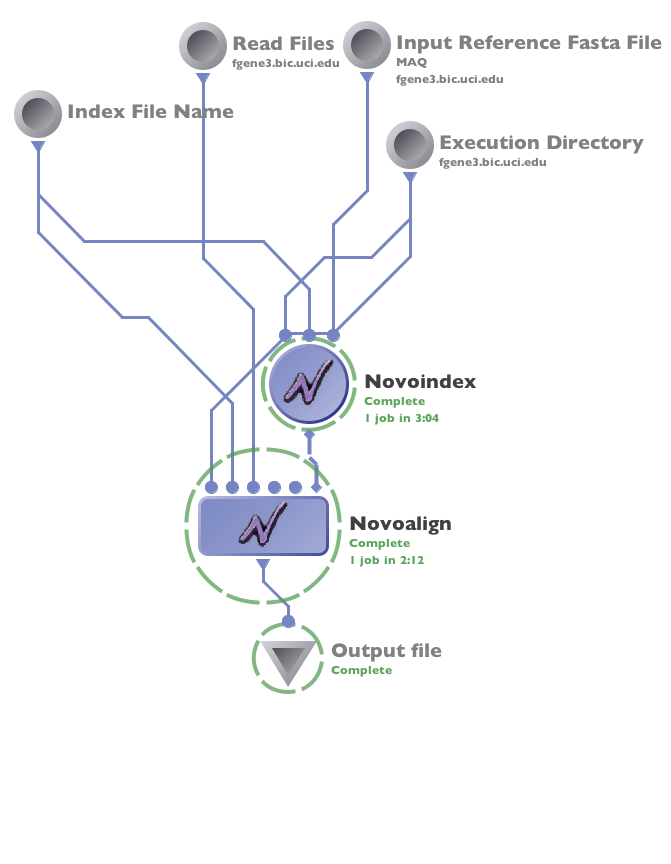


**Figure 9**: A snapshot of the completed Novoalign SE Pipeline workflow.

# PAIRED END ALIGNMENT

# MAQ

## MODULE DESCRIPTION

**GOAL**: align the reads (FASTQ format) to the reference genome with MAQ

**FINAL OUTPUT**: raw .sam file (pre-QC)

To produce the binary reads fastq files the preprocessing pipelines SOLEXA2FASTQ and FQ2BFQ can be used

# ALIGNMENT

### 1.Conversion of the reference genome (fasta format) in binary fasta

Input: reference.fasta file (to perform the alignment)

Tool: MAQ (fasta2bfa option)

Server Location: /applications/maq

Output: reference.bfa file

Example: /applications/maq/maq fasta2bfa /projects1/idinov/projects/Pipeline_genomics_informatics_2011/test_data_2011/ref_chr2.fasta /projects/pipelineCache/pipeline/2011January27_15h51m34s061ms/MAQFasta2BfaConverter_1.Outputbinaryfastafilebfa-1.bfa

Pipeline Module:
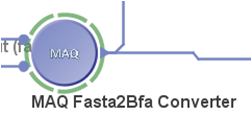


### 2.Alignment to a reference genome

Input: sequence.bfq, reference.bfa

Tool: MAQ (map option)

Server Location: /applications/maq

Output: alignment.map file

Example: /applications/maq/maq map /projects/pipelineCache/pipeline/2011January27_15h51m34s061ms/MAQMapper_1.Outputmappingfilemap-1.map /projects/pipelineCache/pipeline/2011January27_15h51m34s061ms/MAQFasta2BfaConverter_1.Outputbinaryfastafilebfa-1.bfa /projects/pipelineCache/pipeline/2011January27_15h51m34s061ms/MAQFastq2BfdConverter_1.Outputbinaryfastqfilebfq-1.bfq /projects/pipelineCache/pipeline/2011January27_15h51m34s061ms/MAQFastq2BfdConverter_1.Outputbinaryfastqfilebfq-2.bfq

Pipeline Module:
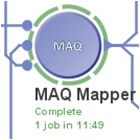


### 3.MAP2SAM conversion

Input: alignment.map file

Tool: samtools (maq2sam-long option)

Server Location: /applications/samtools-0.1.7_x86_64-linux

Output: alignment.sam file

Example:

- Script: /projects1/idinov/projects/Pipeline_genomics_informatics_2011/scripts/maq2sam-long.csh /projects/pipelineCache/pipeline/2011January27_15h51m34s061ms/MAQMapper_1.Outputmappingfilemap-1.map /projects/pipelineCache/pipeline/2011January27_15h51m34s061ms/SamToolsmaq2sam-long_1.OutputSAMfile-1.sam
- Native call: maq2sam-long /ifs/pl_cache/cranium/pipelnvr/2010December03_10h22m38s036ms/MAQMapper_1.Outputmappingfilemap-1.map > /ifs/pl_cache/cranium/pipelnvr/2010December03_10h22m38s036ms/SamToolsmaq2sam-long_2.OutputBAMfile-1.bam

Pipeline Module:
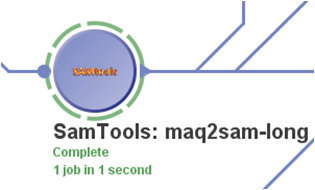


**FINAL MODULE APPEARANCE**


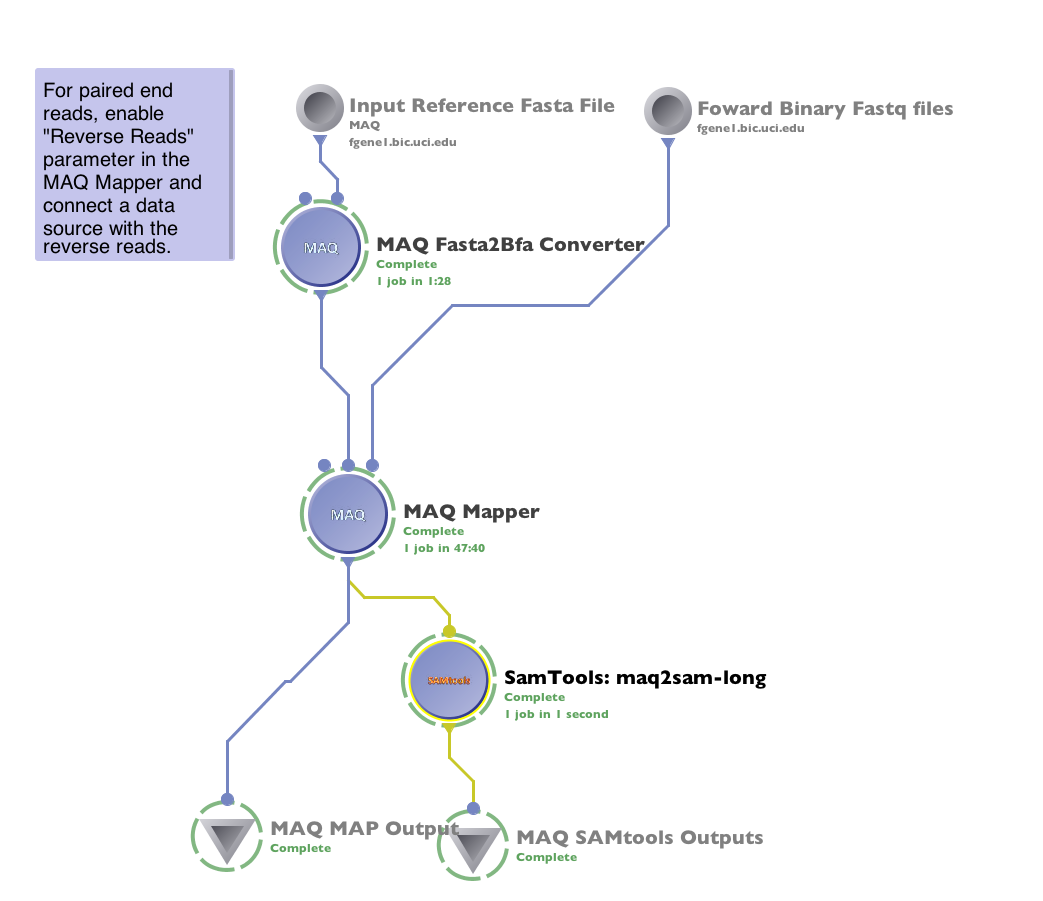


**Figure 10**: A snapshot of the completed MAQ Pipeline workflow.

# BWA_PE

## MODULE DESCRIPTION

**GOAL**: align the reads (FASTQ format) to the reference genome with BWA

**FINAL OUTPUT**: raw .sam file (pre-QC)

# ALIGNMENT

### 1.Indexing the reference file

Input: reference.fasta

Tool: bwa (index option)

Server Location: /applications/BWA/bwa-0.5.9rc1

Output: set of files that are created in the same folder of the reference .fasta file

Example: bwa index /projects1/ADNI_2/BWA/ref-hg18-ensembl/ensembl_hg18_ncbi36_r50.fa

Pipeline Module:


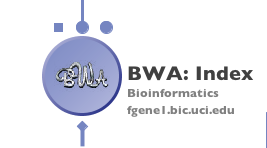


### 2.Forward read file alignment

Input: sequence_read1.fastq file, reference.fasta (along with all the files created in the step I.1. The command line for the alignment points only to the reference.fasta)

Tool: bwa (aln option)

Server Location: /applications/BWA/bwa-0.5.9rc1

Output: sequence_read1.sai

Example: bwa aln reference.fasta sequence_read1.fastq > sequence_read1.sai

Pipeline Module:

**
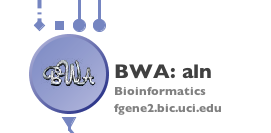
**

### 3.Reverse read file alignment

Input: sequence_read2.fastq file, reference.fasta (along with all the files created in the step I.1. The command line for the alignment points only to the .fasta)

Tool: bwa (aln option)

Server Location: /applications/BWA/bwa-0.5.9rc1

Output: sequence_read2.sai

Example: bwa aln reference.fasta sequence_read2.fastq > sequence_read2.sai

Pipeline Module:

**
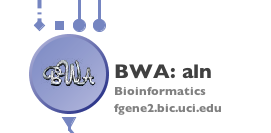
**

### 4.Combined .sam file production

Input: sequence_read1.sai, sequence_read2.sai, sequence_read1.fastq, sequence_read2.fastq

Tool: bwa (sampe option, with )

Server Location: /applications/BWA/bwa-0.5.9rc1

Output: alignment.sam

Usage: bwa sampe [options] <prefix> <in1.sai> <in2.sai> <in1.fq> <in2.fq>

Options:

-a INT maximum insert size [500]

-o INT maximum occurrences for one end [100000]

-n INT maximum hits to output for paired reads [3]

-N INT maximum hits to output for discordant pairs [10]

-c FLOAT prior of chimeric rate (lower bound) [1.0e-05]

-f FILE sam file to output results to [stdout]

-r STR read group header line such as `@RG\tID:foo\tSM:bar' [null]

-P preload index into memory (for base-space reads only)

-s disable Smith-Waterman for the unmapped mate

-A disable insert size estimate (force -s)

Example: bwa sampe reference.fasta sequence_read1.sai, sequence_read2.sai, sequence_read1.fastq, sequence_read2.fastq –r '@RG\tID:JLK-227\tSM:JLK-227' > alignment.sam

Notes:

- the read group id name MUST be formatted as shown, as BWA expects it:

'@RG\tID:YOURID\tSM:YOURSM'

* The entire @RG string surrounded by single quotes (')

* YOURID and YOURSM replaced by your own IDs and SMs, no whitespaces, tabs or colons

Pipeline Module:


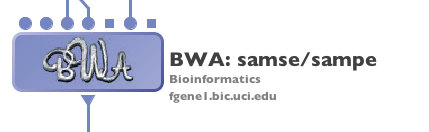


**FINAL MODULE APPEARANCE**


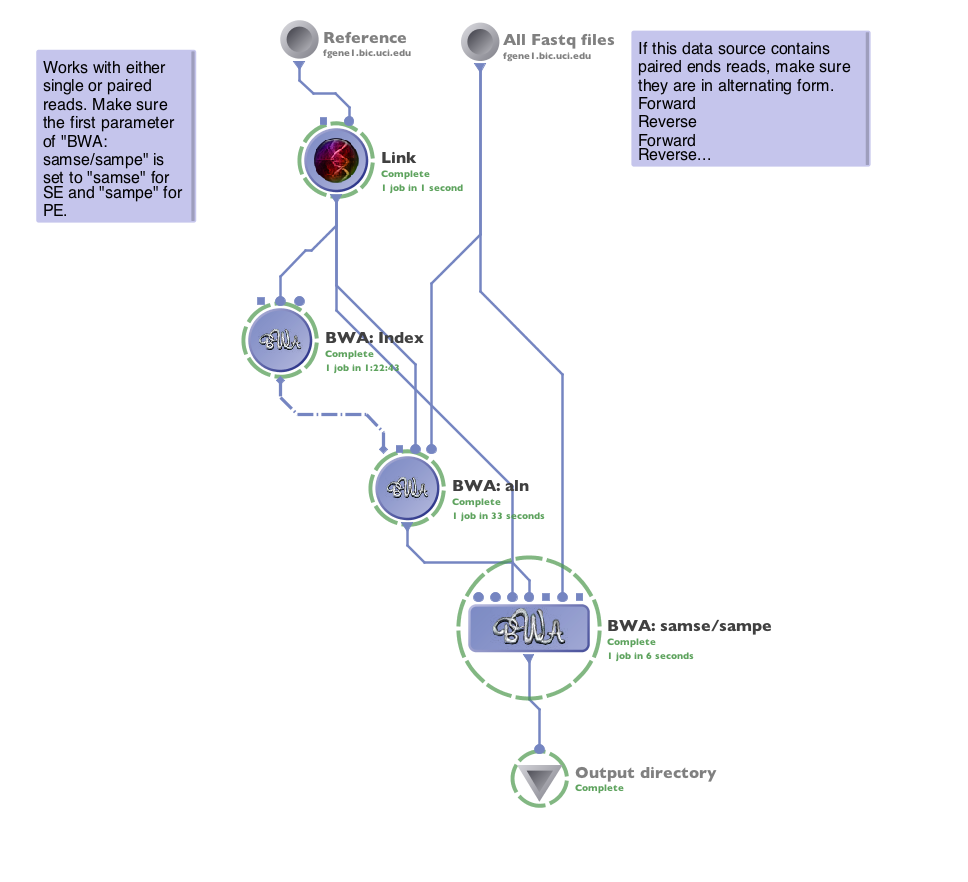


**Figure 11**: A snapshot of the completed BWA (SE and PE have the same appearance) Pipeline workflow.

# PERM

## MODULE DESCRIPTION

**GOAL**: align the reads (FASTQ format) to the reference genome with PERM

**FINAL OUTPUT**: raw .sam file (pre-QC)

# ALIGNMENT

### 1.Alignment

Input: sequence.fastq file, file reference.fasta

Tool: PERM (perm)

Server Location: /applications/PERM/PerM0.3.3Source

Output: alignment.sam, unmapped_reads.fastq, ambiguous_reads.fastq

Example: nohup perm /projects/Miserv/Sequencing/Ref_genomes/human_seguence/chr6.fa -A -a /projects1/TKT_SEQ_2011/UID309_H_reads_morethan200_align.fastq -u UID309_H_unmapped_reads.fastq --log /projects1/TKT_SEQ_2011/UID309_H_perm.log -1 /projects/Miserv/Sequencing/MAQ/Input/FEBIT_tkt/20090828.06s2.1_sequence.fastq -2 /projects/Miserv/Sequencing/MAQ/Input/FEBIT_tkt/20090828.06s2.2_sequence.fastq -o /projects1/TKT_SEQ_2011/perm_UID309_H_chr6.sam > /projects1/TKT_SEQ_2011/perm_UID309_H_chr6.log &

Pipeline Module:

**
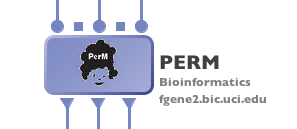
**

**FINAL MODULE APPEARANCE**


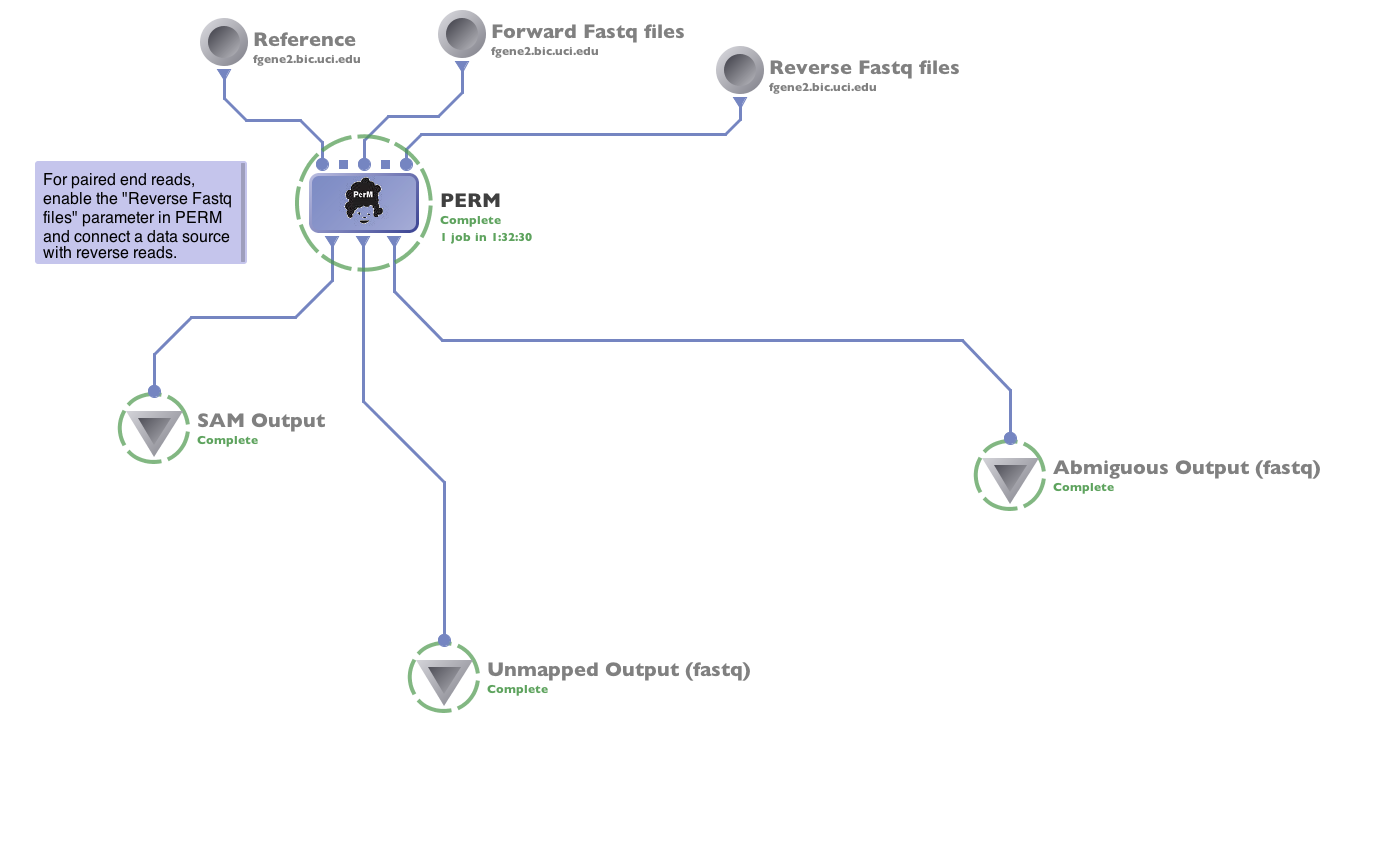


**Figure 12**: A snapshot of the completed PERM PE Pipeline workflow.

# BOWTIE

## MODULE DESCRIPTION

**GOAL**: align the reads (FASTQ format) to the reference genome with BOWTIE

**FINAL OUTPUT**: raw .sam file (pre-QC)

# ALIGNMENT

### 1.Build bowtie index for the reference genome

Input: ref.fa reference files (preferentially the UCSC fasta reference genome, chr1-22,X,Y. See notes if using ensemble genome)

Tool: bowtie (bowtie-build command)

Server Location: /applications/rseqtools/example_dataset/bowtie-0.12.7

Output: series of .ebwt files

Example: bowtie-build ${CD}/human_genome2.fa ucsc_hg18_new_bowtie > \

ucsc_hg18_new_bowtie.log

Pipeline Module:

### 2.Solexa2fastq conversion: conversion of solexa fastq in sanger fastq format

(*note: now this step can be avoided as the recent versions of Bowtie allow using directly the solexa sequence.txt files.See the option –solexa_quals)

Input can be the whole sequence.txt file from Illumina Pipeline or coming from the pre-processing step [Shorter sequence.txt] file.

Input description: reads files output from Illumina sequencing pipeline in solexa format (sequence.txt files)

Label: Illumina reads sequence.txt file/ Shorter sequence.txt file

Tool: MAQ (sol2sanger option)

Server Location: /applications/maq

Output: sequence.fastq file

Example: /applications/maq/maq sol2sanger /projects/pipelineCache/pipeline/2011January27_15h51m34s061ms/SubSequenceExtractor_1.OutputTXTfile-1.txt /projects/pipelineCache/pipeline/2011January27_15h51m34s061ms/MAQSol2SangerConverter_1.Outputfastqfie-1.fastq

Pipeline Module:
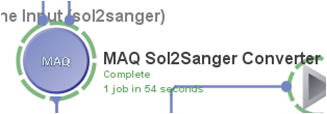


### 3.Bowtie alignment with SAM production

Input: sequence.fastq file (or sequence.txt files, see * note)

Label: Illumina reads sequence.fastq files

Tool: bowtie

Server Location: /applications/BOWTIE/bowtie-0.12.7

Output: alignment.sam

Example: bowtie ${CD}/ucsc_hg18_new_bowtie -1 ${CD}/941408_fwd.fastq -2

${CD}/941408_rev.fastq -v 3 -a -m 600 --best --strata --sam

${CD}/941408_bduc_ucsc_hg18_new.sam >

${CD}/941408_bduc_ucsc_hg18_new.log

Pipeline Module:
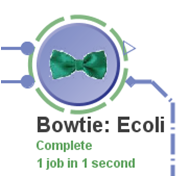


****IMPORTANT**: the flag –solexa-quals has been recently introduced. Convert input qualities from [Solexa](http://en.wikipedia.org/wiki/FASTQ_format#Variations) (which can be negative) to [Phred](http://en.wikipedia.org/wiki/FASTQ_format#Variations) (which can't). This is usually the right option for use with (unconverted) reads emitted by GA Pipeline versions prior to 1.3. Default: off.

the flag --solexa1.3-quals this is usually the right option for use with (unconverted) reads emitted by GA Pipeline version 1.3 or later. Default: off.

These options allow to input into bowtie solexa fastq file directly (sequence.txt) without needing any conversion!!!! (that means avoiding the step 2)

Example: /applications/rseqtools/example_dataset/bowtie-0.12.7/bowtie -a /projects2/USC/rnaseq/rnaseq_reads/RSEQTOOLS/RSEQTOOLS_input_files/hg18 -v 2 -1 s_1_1_sequence.150k.txt -2 s_1_2_sequence.150k.txt --solexa1.3-quals –sam test_bowtie.sam > log.log &

**FINAL MODULE APPEARANCE**


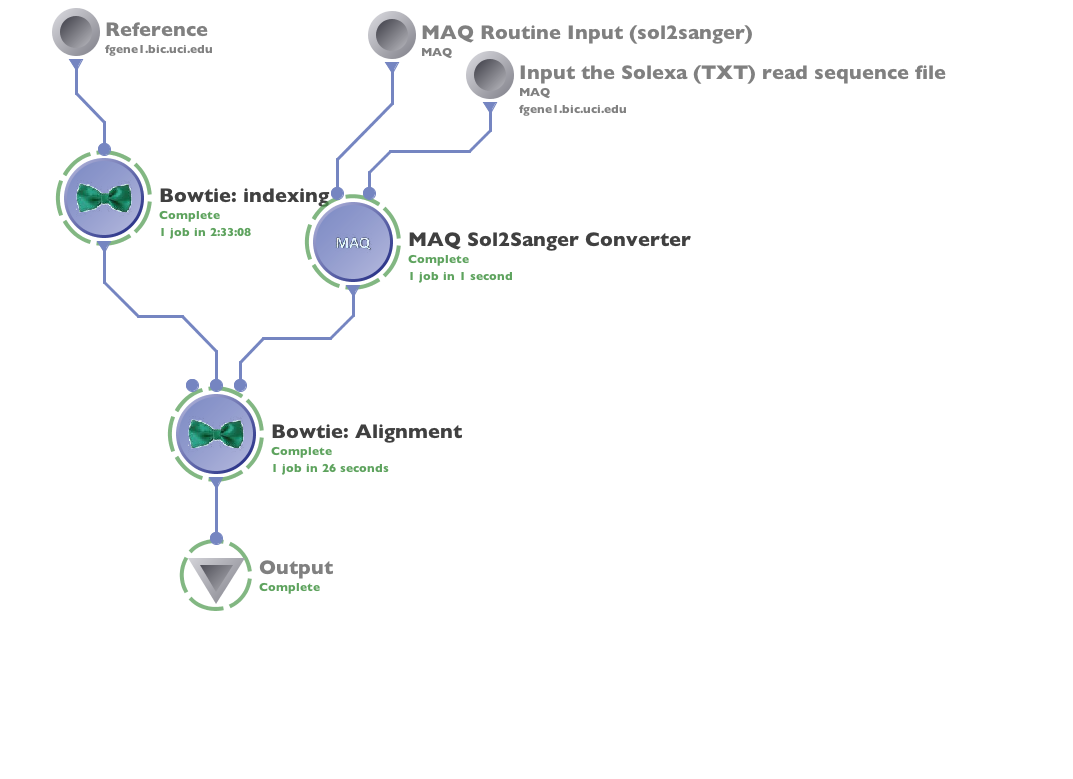


**Figure 13**: A snapshot of the completed Bowtie PE Pipeline workflow.

# SOAP2

## MODULE DESCRIPTION

**GOAL**: align the reads (FASTQ format) to the reference genome with SOAP

**FINAL OUTPUT**: raw .sam file (pre-QC)

# ALIGNMENT

### 1.Format reference sequence:

Input: reference.fasta file

Tool:SOAP2

Server Location <ExecutablePath>/2bwt-builder

Output: 13 index files, all their prefixes are your_fasta file name with “.index” added, e.g. human_genome.fa.index. The suffixes include *.amb, *.ann, *.bwt, *.fmv, *.hot, *.lkt, *.pac, *.rev.bwt, *.rev.fmv, *.rev.lkt, *.rev.pac, *.sa, and *.sai.

Example: ./2bwt-builder ~/human_genome.fa

### 2.Alignment:

Input: reads.fastq

Tool: SOAP2

Server Location: ./soap

Output: alignment.soap

Example: /soap –a <reads_a> -b <reads_b> -D <index.files> -o <PE_output> -2 <SE_output> -m <min_insert_size> -x <max_insert_size>

Pipeline Module:


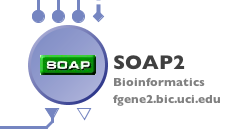


NOTE: For the –D option, the program can only accept the prefix of your index files, such as “~/human_genome.fa.index”.

Options:

-D STR Prefix name for reference index [*.index].

-a STR Query file, for SE reads alignment or one end of PE reads

-b STR Query b file, one end of PE reads

-o STR Output file for alignment results

-2 STR Output file contains mapped but unpaired reads when do PE alignment

-u STR Output file for unmapped reads, [none]

-m INT Minimal insert size INT allowed for PE, [400]

-x INT Maximal insert size INT allowed for PE, [600]

-n INT Filter low quality reads contain more INT bp Ns, [5]

-t Output reads id instead reads name, [none]

-r INT How to report repeat hits, 0=none; 1=random one; 2=all, [1]

-R RF alignment for long insert size(>= 2k bps) PE data, [none] FR alignment

-l INT For long reads with high error rate at 3'-end, those

can't align whole length, then first align 5' INT bp

subsequence as a seed, [256] use whole length of the read

-v INT Totally allowed mismatches in one read, [2]

-M INT Match mode for each read or the seed part of read, which

shouldn't contain more than 2 mismaches, [4]

0: exact match only

1: 1 mismatch match only

2: 2 mismatch match only

3: [gap] (coming soon)

4: find the best hits

-p INT Multithreads, n threads, [1]

### 3.SOAP to SAM conversion

Input: alignment.soap

Tool: soap2sam.pl

Server Location: ./soap2sam.pl (available at http://soap.genomics.org.cn/soapaligner.html)

Output: alignment.sam

Example: soap2sam.pl [-p] <aln.soap>

Pipeline Module:


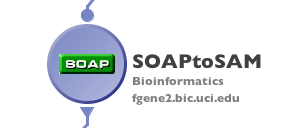


**FINAL MODULE APPEARANCE**


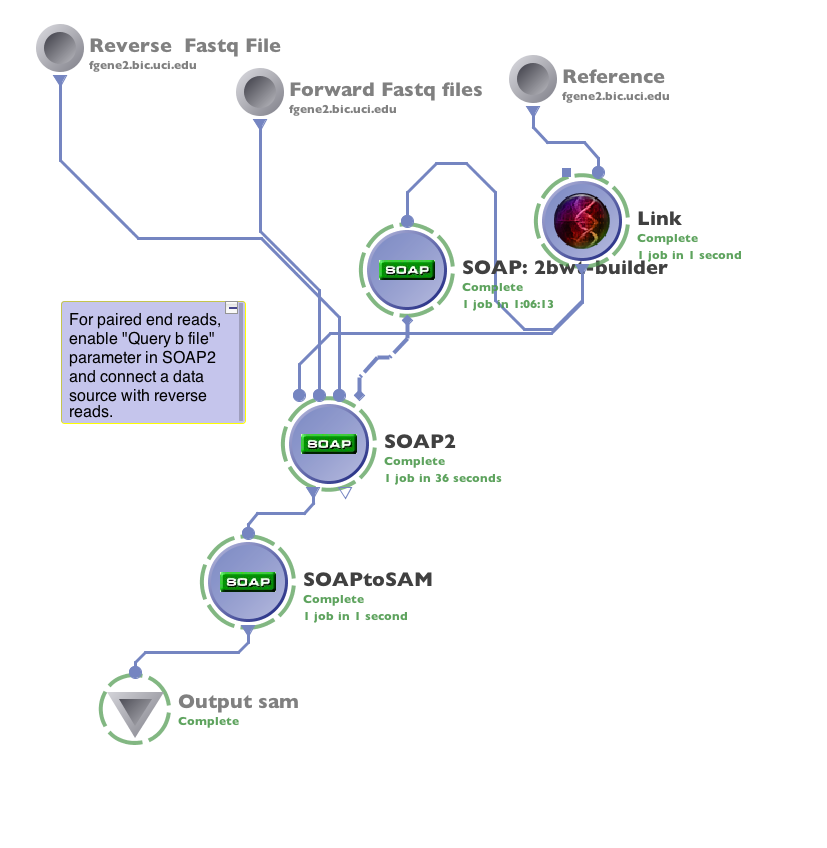


**Figure 14**: A snapshot of the completed SOAPv2 PE Pipeline workflow.

# MOSAIK

## MODULE DESCRIPTION

**GOAL**: align the reads (directly solexa FASTQ format) to the reference genome with MOSAIK

**FINAL OUTPUT**: raw .sam file (pre-QC)

# ALIGNMENT

### 1.Build reference sequence:

Input: reference.fasta file

Tool:.MosaikBuild

Server Location <ExecutablePath>/MosaikBuild

Output: reference.dat

Example: ./MosaikBuild –fr ~/human_genome.fa –oa human_genome.dat

Not: this is the reference in base mode, if needed in color mode add –cs to the command line

Pipeline Module:


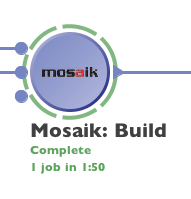


### 2. Create a jumping library of the reference genome

Input: reference.dat

Tool:.MosaikJump

Server Location <ExecutablePath>/MosaikJump

Output: reference_jumping_library

Pipeline Module:


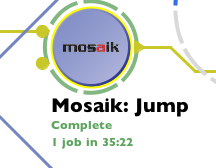


Example: ./MosaikJump –ia human_genome.dat –out human_genome_hs15 -hs 15

### 3.Build paired end reads

Input: sequence_fwd.txt, sequence_rev.txt (reads in solexa fastq format)

Tool:.MosaikBuild

Server Location <ExecutablePath>/MosaikBuild

Output: reads.dat

Example: ./MosaikBuild –q sequence_fwd.txt –q2 sequence_rev.txt –out reads.dat –st Illumina

Pipeline Module:


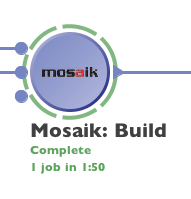


### 4.Alignment

Input: reference.dat, reads.dat, reference_jumping_library

Tool:.MosaikAligner

Server Location <ExecutablePath>/MosaikAligner

Output: reads_aligned_mosaik.dat

Example: ./MosaikAligner –in reads.dat –out reads_aligned_mosaik.dat –ia reference.dat –hs 15 –mm 2 –mhp 100 –j reference_jumping_library

Pipeline Module:


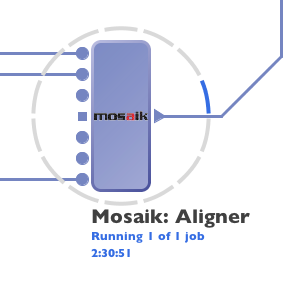


### 5.SAM conversion

Input: reads_aligned_mosaik.dat

Tool:.MosaikText

Server Location <ExecutablePath>/MosaikAligner

Output:

Example: ./MosaikText –in reads_aligned_mosaik.dat –sam reads_aligned_mosaik.sam

IMPORTANT: with the bam argument is possible to export directly in BAM

Pipeline Module:


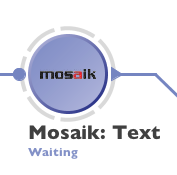


**FINAL MODULE APPEARANCE**


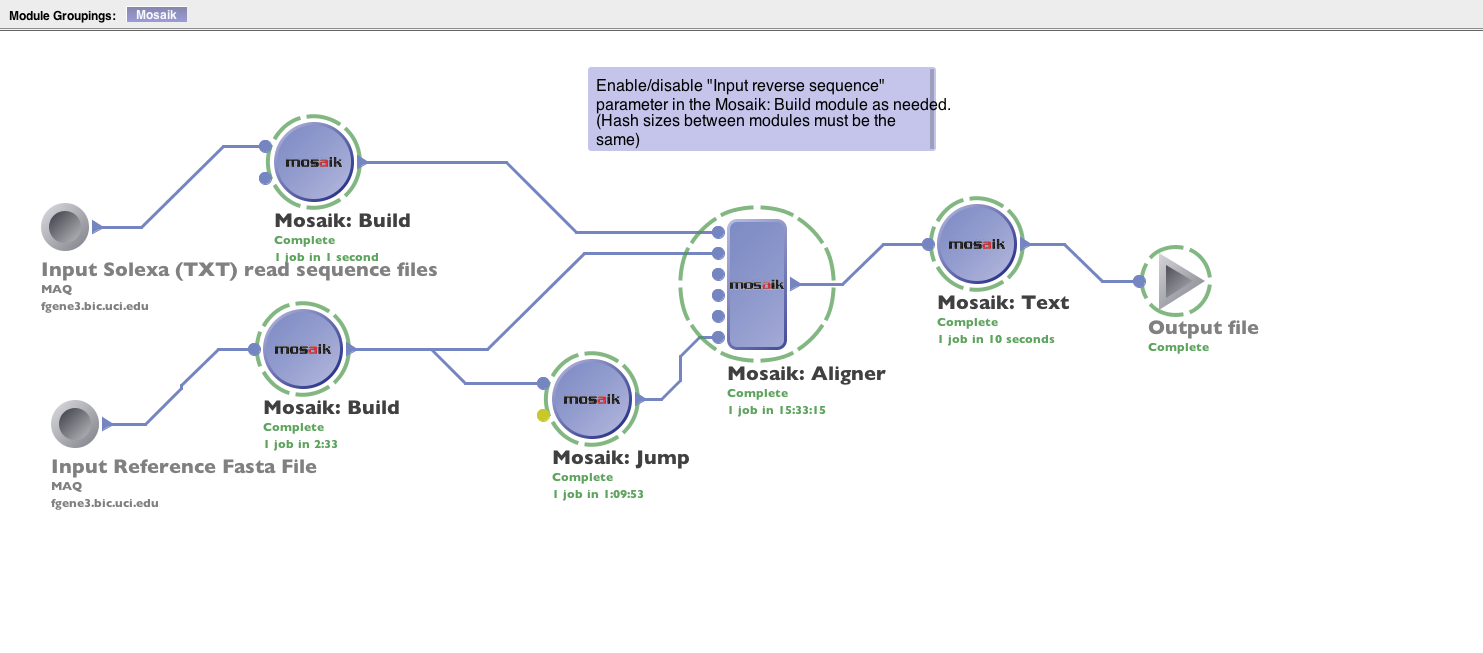


**Figure 15**: A snapshot of the completed Mosaik PE Pipeline workflow.

# NOVOALIGN

## MODULE DESCRIPTION

**GOAL**: align the reads (directly solexa or FASTQ format) to the reference genome with NOVOALIGN

**FINAL OUTPUT**: raw .sam file (pre-QC)

# ALIGNMENT

### 1.Build the indexed reference sequence:

Input: reference.fasta file

Tool:.Novoalign (novoindex.sh)

Server Location: /projects1/Alignment/Novoalign/

Output: reference.nix file

Example: ./novocraft/novoindex ssuis.nix ./sampledata/S_suis.dna

Pipeline Module:

**
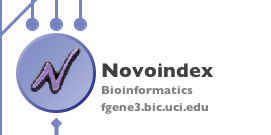
**

### 2.Run Novoalign for Single reads

Input: reference.fasta file (File formats allowed include Solexa PRB, Sanger FASTQ, FASTA, Solexa FASTQ, Illumina FASTQ, and Illumina qseq_txt.)

Tool:Novoalign (novoindex.sh)

Server Location: /projects1/Alignment/Novoalign/

Output: reference.nix file

Example: ./novocraft/novoalign -d ssuis.nix -f ./sampledata/s_1_1_sequence.txt ./sampledata/s_1_2-sequence.txt

Pipeline Module:


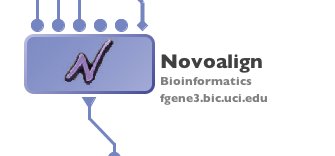


**FINAL MODULE APPEARANCE**


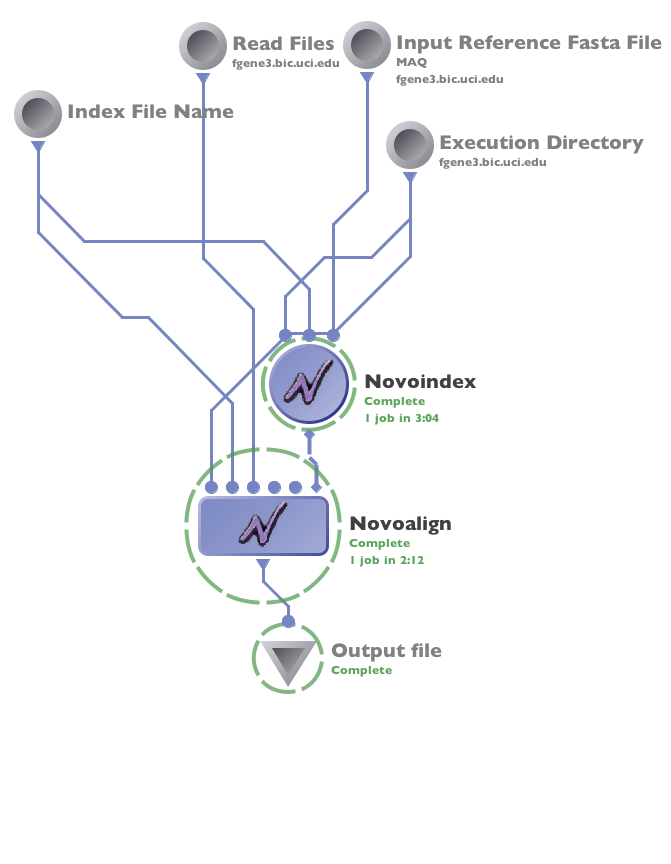


**Figure 16**: A snapshot of the completed Novoalign PE Pipeline workflow.

# (1.2) De novo assembly

# SINGLE END

# SOAP de novo

## MODULE DESCRIPTION

**GOAL**: assembly of the reads with SOAPdenovo

**FINAL OUTPUT**: contigs/scaffolds files

### 1.De novo assembly process

Input: SOAP config file, single-end FASTQ sequence data

Tool: SOAPdenovo release 1.04 (see http://soap.genomics.org.cn/soapdenovo.html)

Server Location: /applications/SOAPdenovo

Output:

*.contig contig sequence file

*.scafSeq scaffold sequence file

Additional files are generated for advanced users (and are not documented very well).

### 2.Command usage:

Version 1.3: released on Nov 23th, 2009

Usage: SOAPdenovo <command> [option]

pregraph construction kmer-graph

contig eliminate errors and output contigs

map map reads to contigs

scaff scaffolding

all doing all the above in turn

[clarkap]$ /SOAPdenovo_Release1.04/SOAPdenovo all

Version 1.3: released on Nov 23th, 2009

SOAPdenovo all -s configFile [-K kmer -d KmerFreqCutOff -D EdgeCovCutoff -M mergeLevel -R -u -G gapLenDiff -L minContigLen -p n_cpu] -o Output

-s ShortSeqFile: The input file name of solexa reads

-K kmer(default 23): k value in kmer

-p n_cpu(default 8): number of cpu for use

-M mergeLevel(default 1,min 0, max 3): the strength of merging similar sequences during contiging

-d KmerFreqCutoff(optional): delete kmers with frequency no larger than (default 0)

-D EdgeCovCutoff(optional): delete edges with coverage no largert than (default 1)

-R (optional): unsolve repeats by reads (default no)

-G gapLenDiff(default 50): allowed length difference between estimated and filled gap

-L minLen(default K+2): shortest contig for scaffolding

-u (optional): un-mask contigs with high coverage before scaffolding (default mask)

-o Output: prefix of output file name

Pipeline Module:


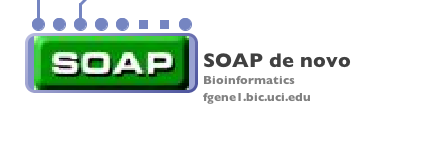


**3.Example invocation:**

Contents of soap_config file:

max_rd_len=100

[LIB]

reverse_seq=0

asm_flags=3

q=/path/to/read1.fastq

Set K-mer size to 31, use 16 threads, set output filename prefix to “rockfish-70m-31k” and try to resolve small repeats (-R option):

$SOAPDENOVO all -s soap_config -K 31 -R -p 16 -o rockfish-70m-31k

Pipeline Module:


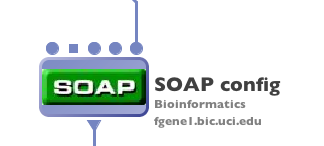


**FINAL MODULE APPEARANCE**


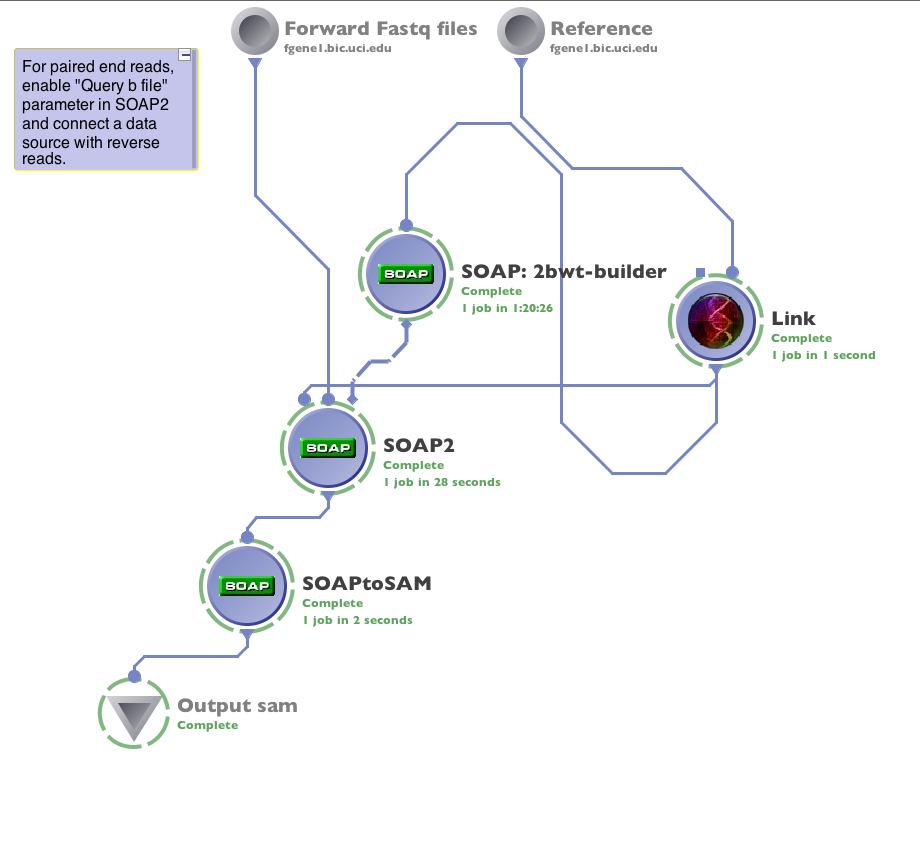


**Figure 17**: A snapshot of the completed SOAPdeNOVO SE Pipeline workflow.

## MODULE DESCRIPTION

**GOAL**: assembly of single end reads with VELVET

**FINAL OUTPUT**: contig files

### 1.Input preparation: conversion fastq2fasta single end reads

Input: sequence_fwd.txt (reads on solexa fastq format)

Tool: Fastx_toolkit (fasta_to_fasta)

Server Location: <ExecutablePath>/Fastx_toolkit/ bin/fastq_to_fasta

Output: sequence_fwd.fa

Example: /opt/fastx_toolkit-0.0.10/bin/fastq_to_fasta -n -i sequence.txt -o sequence.fa

Pipeline Module:

*
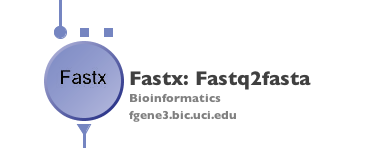
*

### 2. VELVETH: creation of the hash

Input: sequence_fwd.fa

Tool: Velvet (velveth)

Server Location: <ExecutablePath>/velvet/velveth

Output: VelvetResults_directory

Example: /opt/velvet/velveth 21 path/VelvetResults_directory –short –fasta _velvet_input.fa

Pipeline Module:

*
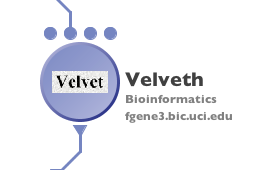
*

### 3. VELVETG: assembly (Bruijn graph building)

Input: VelvetResults_directory

Tool: Velvet (velvetg)

Server Location: <ExecutablePath>/velvet/velvetg

Output: contigs.fa, stats.txt

Example: /opt/velvet/velvetg path/VelvetResults_directory > velvetSE.log

Pipeline Module:


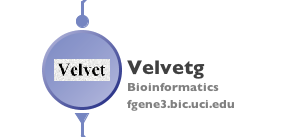


**FINAL MODULE APPEARANCE**


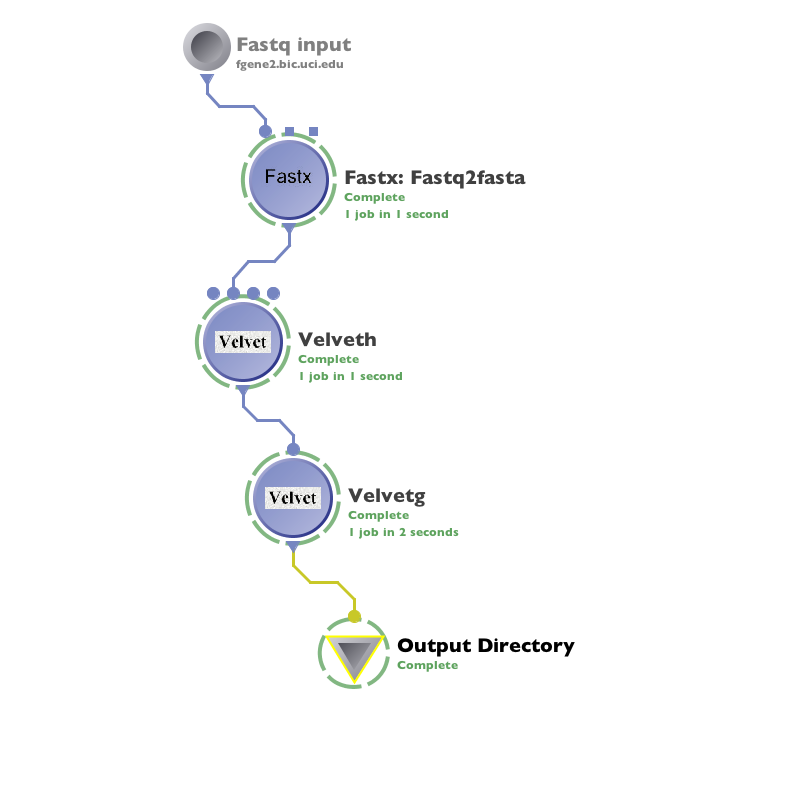


**Figure 18**: A snapshot of the completed Velvet SE Pipeline workflow.

## ABYSS

**GOAL**: assembly of paired end reads with ABYSS

**FINAL OUTPUT**: contigs file

### 1.Input preparation: conversion fastq2fasta single end reads

Input: sequence_fwd.txt (reads on solexa fastq format)

Tool: Fastx_toolkit (fasta_to_fasta)

Server Location: <ExecutablePath>/Fastx_toolkit/ bin/fastq_to_fasta

Output: sequence_fwd.fa

Example: /opt/fastx_toolkit-0.0.10/bin/fastq_to_fasta -n -i sequence.txt -o sequence.fa

Pipeline Module:


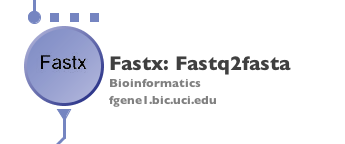


### 2.De novo assembly

Input: sequence_fwd.fa

Tool: ABYSS

Server Location: <ExecutablePath>/Abyss/ABYSS

Output: abyss.contigs.fa

Example: ABYSS –k25 sequence_fwd.fa –o abyss.contigs.fa

Pipeline Module:

*
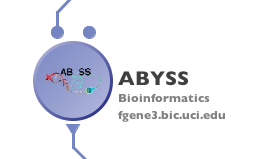
*

Note from the README:

-k is an appropriate k-mer length. The only method to find the optimal value of k is to run multiple trials and inspect the results. The following shell snippet will assemble for every value of k from 20

to 40.

for k in {20..40}; do

ABYSS -k$k reads.fa -o contigs-k$k.fa

done

The maximum value for k is 64. This limit may be changed at compile time using the --enable-maxk option of configure. It may be decreased to 32 to decrease memory usage, which is particularly useful for large parallel jobs, or increased to 96.

**FINAL MODULE APPEARANCE**


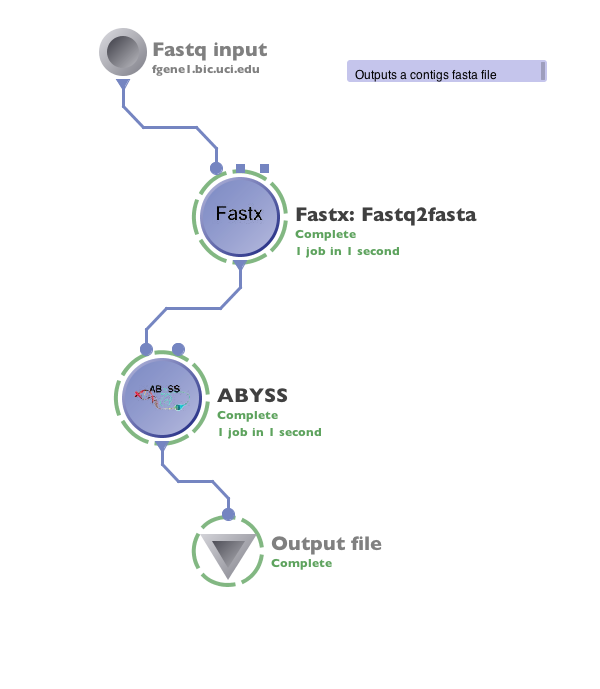


**Figure 19**: A snapshot of the completed Abyss SE Pipeline workflow.

# PAIRED END

## SOAP de novo

## MODULE DESCRIPTION

**GOAL**: assembly of the paired end reads with SOAPdenovo

**FINAL OUTPUT**: contigs/scaffolds files

### 1.De novo assembly process

Input: SOAP config file, paired-end FASTQ sequence data (separate files for forward and reverse reads)

Tool: SOAPdenovo release 1.04 (see http://soap.genomics.org.cn/soapdenovo.html)

Server Location: To be installed locally…

Output:

*.contig contig sequence file

*.scafSeq scaffold sequence file

Additional files are generated for advanced users (and are not documented very well).

### 2.Command usage:

Version 1.3: released on Nov 23th, 2009

Usage: SOAPdenovo <command> [option]

pregraph construction kmer-graph

contig eliminate errors and output contigs

map map reads to contigs

scaff scaffolding

all doing all the above in turn

[clarkap]$ /SOAPdenovo_Release1.04/SOAPdenovo all

Version 1.3: released on Nov 23th, 2009

SOAPdenovo all -s configFile [-K kmer -d KmerFreqCutOff -D EdgeCovCutoff -M mergeLevel -R -u -G gapLenDiff -L minContigLen -p n_cpu] -o Output

-s ShortSeqFile: The input file name of solexa reads

-K kmer(default 23): k value in kmer

-p n_cpu(default 8): number of cpu for use

-M mergeLevel(default 1,min 0, max 3): the strength of merging similar sequences during contiging

-d KmerFreqCutoff(optional): delete kmers with frequency no larger than (default 0)

-D EdgeCovCutoff(optional): delete edges with coverage no largert than (default 1)

-R (optional): unsolve repeats by reads (default no)

-G gapLenDiff(default 50): allowed length difference between estimated and filled gap

-L minLen(default K+2): shortest contig for scaffolding

-u (optional): un-mask contigs with high coverage before scaffolding (default mask)

-o Output: prefix of output file name

**3.Example invocation:**

Contents of soap_config file:

max_rd_len=100

[LIB]

avg_ins=361

reverse_seq=0

asm_flags=3

q1=/path/to/read1.fastq

q2=/path/to/read2.fastq

Set K-mer size to 31, use 16 threads, set output filename prefix to “rockfish-70m-31k” and try to resolve small repeats (-R option):

$SOAPDENOVO all -s soap_config -K 31 -R -p 16 -o rockfish-70m-31k

Pipeline Module:


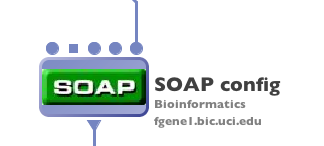


**FINAL MODULE APPEARANCE**


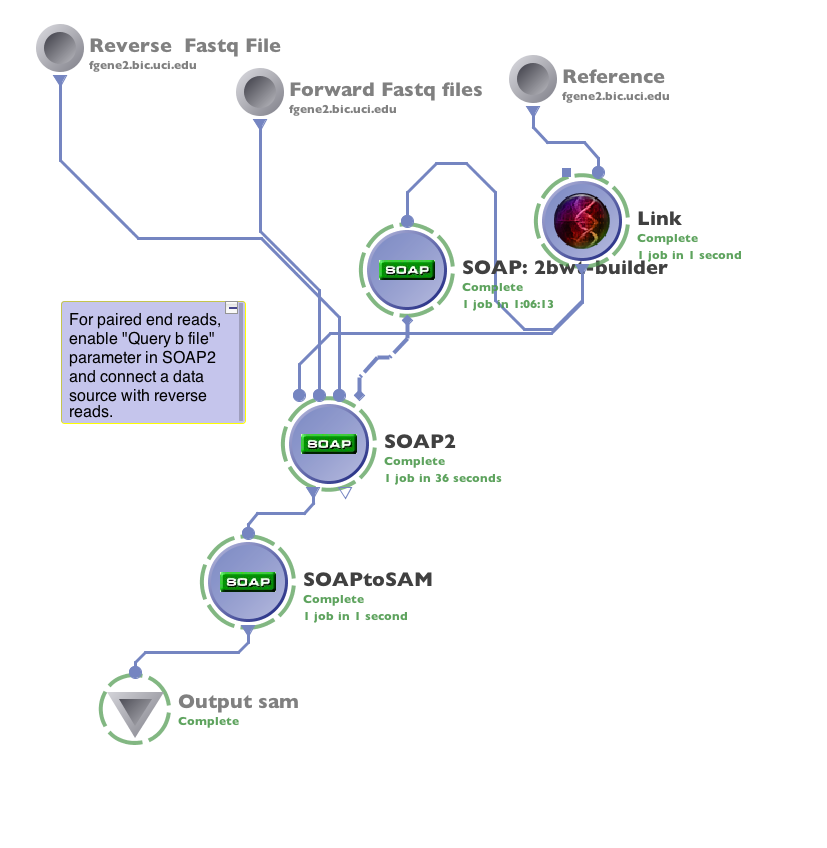


**Figure 20**: A snapshot of the completed SOAPdeNOVO PE Pipeline workflow.

## VELVET

## MODULE DESCRIPTION

**GOAL**: assembly of paired end reads with VELVET

**FINAL OUTPUT**: contigs file

### 1.Input preparation: conversion fastq2fasta forward reads

Input: sequence_fwd.txt (reads on solexa fastq format)

Tool: Fastx_toolkit (fasta_to_fasta)

Server Location: <ExecutablePath>/Fastx_toolkit/ bin/fastq_to_fasta

Output: sequence_fwd.fa

Example: /opt/fastx_toolkit-0.0.10/bin/fastq_to_fasta -n -i sequence_fwd.txt -o sequence_fwd.fa

Pipeline Module:

*
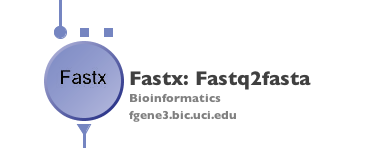
*

### 2.Input preparation: conversion fastq2fasta reverse reads

Input: sequence_rev.txt (reads on solexa fastq format)

Tool: Fastx_toolkit (fasta_to_fasta)

Server Location: <ExecutablePath>/Fastx_toolkit/ bin/fastq_to_fasta

Output: sequence_rev.fa

Example: /opt/fastx_toolkit-0.0.10/bin/fastq_to_fasta -n -i sequence_rev.txt -o sequence_rev.fa

Pipeline Module:

*
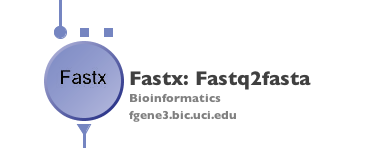
*

### 3. VELVET INPUT PREPARATION

Input: sequence_fwd.fa ,sequence_rev.fa

Tool: Velvet (shuffleSequences_fasta.pl)

Server Location: <ExecutablePath>/Fastx_toolkit/ bin/fastq_to_fasta

Output: velvet_input.fa

Example: /opt/velvet/shuffleSequences_fasta.pl sequence_fwd.fa sequence_rev.fa velvet_input.fa

Pipeline Module:

*
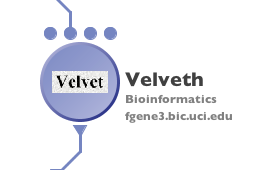
*

### 4. VELVETH: creation of the hash

Input: velvet_input.fa

Tool: Velvet (velveth)

Server Location: <ExecutablePath>/velvet/velveth

Output: VelvetResults_directory

Example: /opt/velvet/velveth 21 path/VelvetResults_directory –shortPaired –fasta _velvet_input.fa

Pipeline Module:

*
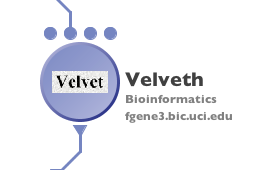
*

### 5. VELVETG: assembly (Bruijn graph building)

Input: VelvetResults_directory

Tool: Velvet (velvetg)

Server Location: <ExecutablePath>/velvet/velvetg

Output: contigs.fa, stats.txt

Example: /opt/velvet/velvetg path/VelvetResults_directory ins_length 250 –exp_cov auto > velvetPE.log

Pipeline Module:


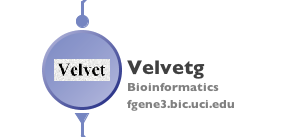


**FINAL MODULE APPEARANCE**


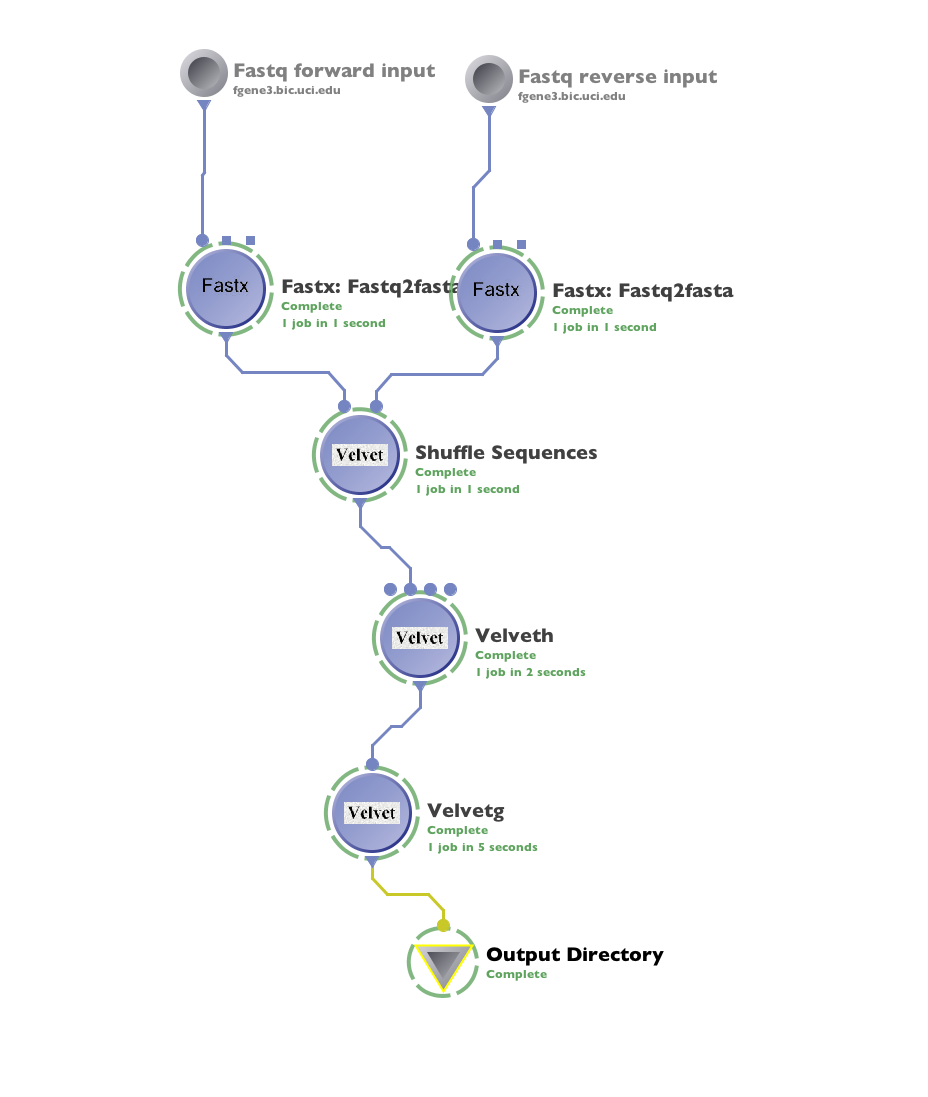


**Figure 21**: A snapshot of the completed Velvet PE Pipeline workflow.

## ABYSS

## MODULE DESCRIPTION

**GOAL**: assembly of paired end reads with ABYSS

**FINAL OUTPUT**: contigs file

### 1.Input preparation: conversion fastq2fasta forward reads

Input: sequence_fwd.txt (reads on solexa fastq format)

Tool: Fastx_toolkit (fasta_to_fasta)

Server Location: <ExecutablePath>/Fastx_toolkit/ bin/fastq_to_fasta

Output: sequence_fwd.fa

Example: /opt/fastx_toolkit-0.0.10/bin/fastq_to_fasta -n -i sequence_fwd.txt -o sequence_fwd.fa

Pipeline Module:


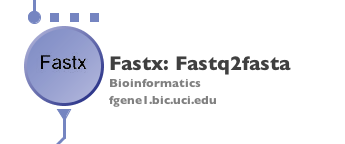


### 2.Input preparation: conversion fastq2fasta reverse reads

Input: sequence_rev.txt (reads on solexa fastq format)

Tool: Fastx_toolkit (fasta_to_fasta)

Server Location: <ExecutablePath>/Fastx_toolkit/ bin/fastq_to_fasta

Output: sequence_rev.fa

Example: /opt/fastx_toolkit-0.0.10/bin/fastq_to_fasta -n -i sequence_rev.txt -o sequence_rev.fa

Pipeline Module:


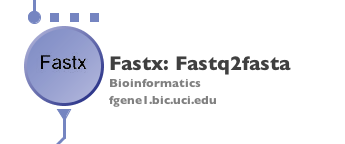


### 3.De novo assembly

Input: sequence_fwd.fa, sequence_rev.fa

Tool: abyss-pe

Server Location: <ExecutablePath>/Abyss/abyss-pe

Output: abyssPE.contigs

Example: abyss-pe k=25 n=5 in=‘sequence_fwd.fa sequence_rev.fa’ name=abyssPE

where k is the k-mer length as before.

n is the minimum number of pairs needed to consider joining two

contigs. The optimal value for n must be found by trial.

in specifies the input files to read, which may be in FASTA, FASTQ,

qseq, export, SAM or BAM format and compressed with gz, bz2 or xz and

may be tarred.

Pipeline Module:

*
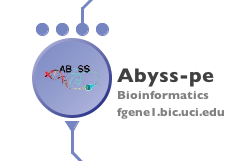
*

**FINAL MODULE APPEARANCE**


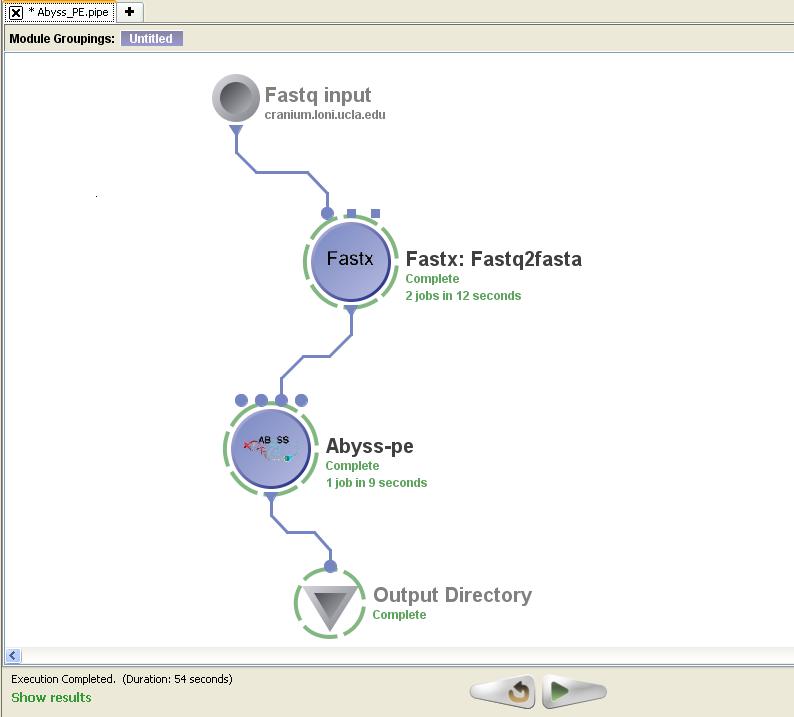


**Figure 22**: A snapshot of the completed Abyss PE Pipeline workflow.

# (1.3) BASIC QC and formatting of BAM files

# GLOSSARY and MODULE OVERVIEW

“Input”: name of the input file

“Label”: is specified when the name of the input on the pipeline canvas has to be slightly different form the one specified in the Input section to be more clear.

“Tool”: script/program in use

“Server Location”: location on the fgene server

“Output”: name of the output fil

**
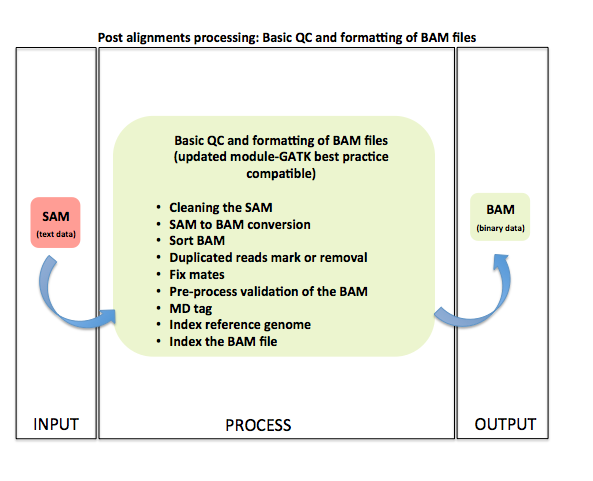
**


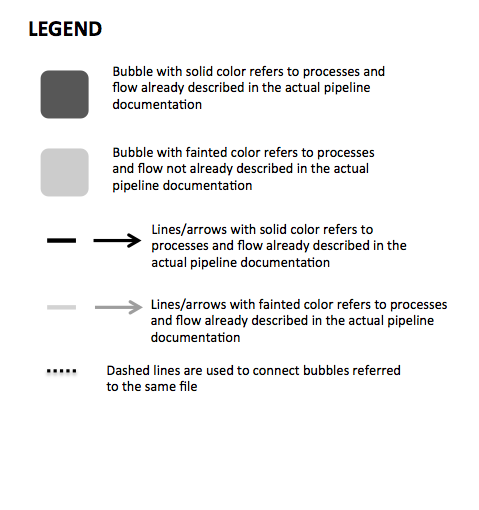


# MODULE DESCRIPTION

**GOAL**: to produce a .bam file sorted, indexed, with mate information fixed and cleaned (soft-clip an alignment that hangs off the end of its reference sequence), with duplicate reads removed OR marked, and ready for the next steps of advanced QC.

**FINAL OUTPUT**: .bam and .bai files

### Cleaning the SAM

Input: alignment.sam

Tool: PICARD (CleanSam.jar)

Server Location: /applications/picard_1.38/picard-tools-1.38

Output: alignment_clean.sam

Example: java –jar /applications/picard_1.38/picard-tools-1.38/CleanSam.jar INPUT=alignment.sam OUTPUT=alignment_clean.sam

Pipeline Module:


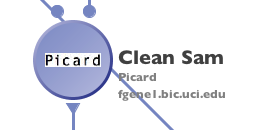


### SAM to BAM conversion

Input: alignment_clean.sam

Tool: PICARD (SamFormatCoverter.jar)

Server Location: /applications/picard_1.38/picard-tools-1.38

Output: alignment.bam file

Example: java -jar /applications/picard_1.51/picard-tools-1.51/SamFormatConverter.jar INPUT=samfile-0_picard_sorted.sam OUTPUT=samfile-0_picard_sorted.bam VALIDATION_STRINGENCY=SILENT

Pipeline Module:


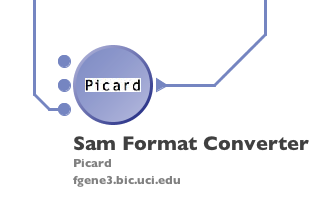


### Sort .bam

Input: alignment.bam file

Tool: PICARD (SortSam.jar)

Server Location: /applications/picard_1.38/picard-tools-1.38

Output: alignment.sorted.bam file

Example: java -jar /applications/picard_1.51/picard-tools-1.51/SortSam.jar INPUT=samfile-0.sam OUTPUT=samfile-0_picard_sorted.sam SORT_ORDER=coordinate

Pipeline Module:


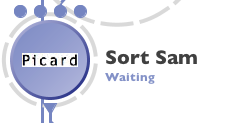


### Mark duplicates OR Remove duplicates

Input: alignment.sorted.bam

Tool:PICARD (MarkDuplicates.jar )

Server Location: /applications/picard_1.38/picard-tools-1.38

Output: alignment.sorted.dup.bam

Example: nohup /applications/picard_1.38/picard-tools-1.38/MarkDuplicates.jar INPUT=samfile-0_picard_sorted.bam OUTPUT=samfile-0_picard_sorted_marked.bam METRiCS_FILE=mark_metrics.txt REMOVE_DUPLICATES=false VALIDATION_STRINGENCY=SILENT

Pipeline Module:


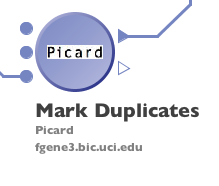


### Fix Mates

Input: alignment.sorted.dup.bam file

Tool: PICARD (FixMateInformation.jar)

Server Location: /applications/picard_1.38/picard-tools-1.38

Output: alignment.sorted.dup.fm.bam file

Example: java –jar /applications/picard_1.38/picard-tools-1.38/ FixMateInformation.jar INPUT=alignment.sorted.bam OUTPUT= alignment.sorted.fm.bam

Pipeline Module:


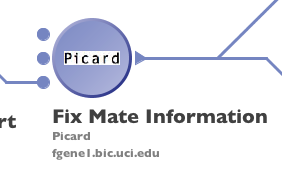


### Preprocess-VALIDATION of the BAM

Input: alignment.sorted.dup.fm.bam

Tool:PICARD(ValidateSamFile.jar )

Server Location: /applications/picard_1.38/picard-tools-1.38

Output: file with validation information

Example:/applications/picard_1.34/picard-tools-1.34/ValidateSamFile.jar INPUT=/projects1/USC/production/data/852603/852603.bam OUTPUT=852603.bam.validation

Pipeline Module:


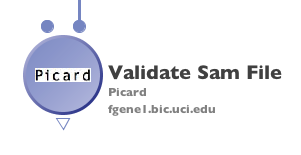


### Index the reference genome

Input: reference.fa

Tool: samtools (faidx option)

Server Location: /applications/samtools-0.1.7_x86_64-linux

Output: reference.fai

Example: /applications/samtools-0.1.7_x86_64-linux/samtools faidx /projects1/idinov/projects/Pipeline_genomics_informatics_2011/test_data_2011/ref_chr2.fasta

Pipeline Module:


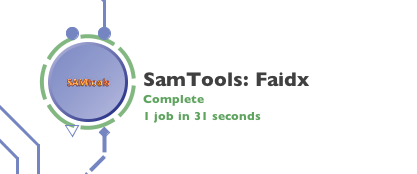


### MD tag

Input: alignment.sorted.dup.fm.bam file and reference REF.fasta file

Tool: samtools (md option)

Server Location: /applications/samtools-0.1.7_x86_64-linux

Output: alignment.basicQC.final.bam file

Example: /projects1/idinov/projects/Pipeline_genomics_informatics_2011/scripts/samtools_md_pipeline.sh md -b /projects/pipelineCache/pipeline/2011January27_15h51m34s061ms/SamToolsSort_1.OutputSortedBAMfile-1.bam /projects1/idinov/projects/Pipeline_genomics_informatics_2011/test_data_2011/ref_chr2.fasta /projects/pipelineCache/pipeline/2011January27_15h51m34s061ms/SamToolsCamldMDtag_1.OutputNo-DuplicatesBAMfile-1.bam

Pipeline Module:
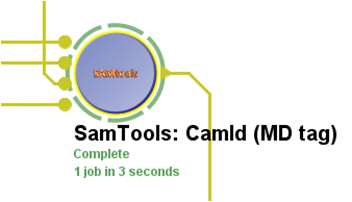


### Indexing the .bam file

Input: alignment.basicQC.final.bam file

Tool: PICARD (BuildBamIndex.jar)

Server Location: /applications/picard_1.38/picard-tools-1.38

Output: alignment.basicQC.final.bam.bai file

Example:

/applications/picard_1.34/picard-tools-1.34/BuildSamIndex.jar INPUT=/projects1/USC/production/data/852603/852603.bam OUTPUT=852603.bam.bai

Pipeline Module:

**FINAL MODULE APPEARANCE**

**Figure 23**: A snapshot of the completed Basic QC Pipeline workflow

# (1.4) ADVANCED data QC and cleaning of BAM files

# GENERAL GLOSSARY and MODULE OVERVIEW

“Input”: name of the input file

“Label”: is specified when the name of the input on the pipeline canvas has to be slightly different form the one specified in the Input section to be more clear.

“Tool”: script/program in use

“Server Location”: location on the fgene server

“Output”: name of the output file

## MODULE DESCRIPTION

**GOAL**: align the reads (FASTQ format) to the reference genome with MAQ

**FINAL OUTPUT**: raw .bam file (pre-QC)

#note: for all the GATK modules is needed that the .bam file and the .bai are in the same folder. This is why there are so many indexing steps.

## Local Realignment Around Indels

(general guide: http://www.broadinstitute.org/gsa/wiki/index.php/Local_realignment_around_indels) :

### 1.1 Interval creation

(see <http://www.broadinstitute.org/gsa/wiki/index.php/Local_realignment_around_indels#Fixing_Mate_Pairs>, section Creating Intervals)

Input: alignment.sorted.md.bam, (alignment.sorted.md.bam.bai has to be in the same folder in the same folder)

Tool: GATK (GenomeAnalysisTK.jar, -T RealignerTargetCreator)

Server Location: /applications/gatk/GATK-2010-01-14/dist

Output: forRealigner.intervals

Example: java -Xmx4g -jar GenomeAnalysisTK.jar -T RealignerTargetCreator -I /projects1/ADNI_2/BWA/Input/s_1_1_sequence.s_1_2_sequence.mapped.sorted.md.bam -R /projects/USC/production/ref/ensembl_ncbi36/ensembl_hg18_ncbi36_r50.fa -o forRealigner.intervals -D /applications/GATK/resources/dbsnp_129_b36.rod

Optional parameters:

[-L intervals]: to restrict the search to a specific region or set of regions instead of the whole genome

[-B:snps,VCF /path/to/SNP_calls.vcf]; restrict on a list on SNPs already called to find clusters of SNPs

[-B:indels,VCF /path/to/indel_calls.vcf]: known indel location

[-D /path/to/dbsnp.rod] : known snps location

Pipeline Module:

### 1.2 Realignment

(see <http://www.broadinstitute.org/gsa/wiki/index.php/Local_realignment_around_indels#Fixing_Mate_Pairs>, section REALIGNING)

Input: alignment.sorted.bam, alignment.sorted.md.bai

Tool: GATK (GenomeAnalysisTK.jar, -T IndelRealigner)

Server Location: /applications/gatk/GATK-2010-01-14/dist

Output: alignment.sorted.md.realign.bam

Example: java -Djava.io.tmpdir=/path/to/tmpdir java -Xmx4g -jar GenomeAnalysisTK.jar -I /projects1/ADNI_2/BWA/Input/s_1_1_sequence.s_1_2_sequence.mapped.sorted.md.bam -R /projects/USC/production/ref/ensembl_ncbi36/ensembl_hg18_ncbi36_r50.fa -T IndelRealigner -targetIntervals forRealigner.intervals --output /projects1/ADNI_2/BWA/Input/s_1_1_sequence.s_1_2_sequence.mapped.sorted.md.realign.bam -D /applications/GATK/resources/dbsnp_129_b36.rod

Pipeline Module:

### 1.3 Indexing of the BAM file

Input: alignment.sorted.md.realign.bam

Tool: PICARD (BuildBamIndex.jar)

Server Location: /applications/picard_1.38/picard-tools-1.38

Output: alignment.sorted.md.realign.bam.bai

Example: /applications/picard_1.34/picard-tools-1.34/BuildSamIndex.jar INPUT=/projects1/USC/production/data/852603/852603.bam OUTPUT=852603.bam.bai

Pipeline Module:

### 1.4 Mark duplicates

Input: alignment.sorted.md.realign.bam, reference.fasta file

Tool:PICARD(MarkDuplicates.jar )

Server Location: /applications/picard_1.38/picard-tools-1.38

Output: alignment.sorted.md.realign.dupm.bam

Example: java -jar MarkDUplicates.jar INPUT=/projects1/ADNI_2/BWA/Input/s_1_1_sequence.s_1_2_sequence.mapped..sorted.md.realign.bam OUTPUT=/projects1/ADNI_2/BWA/Input/s_1_1_sequence.s_1_2_sequence.mapped..sorted.md.realign.dupm.bam METRICS_FILE==/projects1/ADNI_2/BWA/Input/metrics_dupm.txt REMOVE_DUPLICATES=true (keep them) or false (remove them)

Pipeline Module:

## Base Quality recalibration

(general guide: <http://www.broadinstitute.org/gsa/wiki/index.php/Base_quality_score_recalibration>)

### 2.1 Covariates calculation

(<http://www.broadinstitute.org/gsa/wiki/index.php/Base_quality_score_recalibration>, section CountCovariates)

Input: alignment.sorted.md.realign.dupm.bam, reference.fasta file

Tool: GATK (GenomeAnalysisTK.jar, -T CountCovariates )

Server Location: /applications/gatk/GATK-2010-01-14/dist

Output: recal_1.csv file (table of the covariates counts)

Example: java -Xmx4g -jar GenomeAnalysisTK.jar -l INFO -R /projects/USC/production/ref/ensembl_ncbi36/ensembl_hg18_ncbi36_r50.fa --DBSNP /applications/GATK/resources/dbsnp_129_b36.rod -I /projects1/ADNI_2/BWA/Input/s_1_1_sequence.s_1_2_sequence.mapped.sorted.md.realign.dupm.bam -T CountCovariates -cov ReadGroupCovariate -cov QualityScoreCovariate -cov CycleCovariate -cov DinucCovariate -recalFile JLK-227.recal.csv --default_platform illumina

Pipeline Module:

### 2.2 Covariates analysis: plotting score parameters before recalibration

Input: recal_1.csv (table of the covariates counts)

Tool: GATK (AnalyzeCovariates.jar) (need to locate also Rscript)

Server Location: /applications/gatk/GATK-2010-01-14/dist

Output: plots (.pdf files) and .dat files

Example: java -jar AnalyzeCovariates.jar -recalFile /applications/GATK_source/usc-build/dist/JLK-227.recal.csv -outputDir /applications/GATK_source/usc-build/dist/test -resources /applications/GATK_source/Sting/R -Rscript /applications/R-2.11.1/bin/Rscript

Pipeline Module:

### 2.3 Recalibration

(<http://www.broadinstitute.org/gsa/wiki/index.php/Base_quality_score_recalibration>, section Table recalibration)

Input: alignment.sorted.md.realign.dupm.bam, reference.fasta file, recal_1.csv Tool: GATK (GenomeAnalysisTK.jar, -T TableRecalibration)

Server Location: /applications/gatk/GATK-2010-01-14/dist

Output:alignment.rmd.sorted.md.clean.bam

Example: nohup java -Xmx4g -jar GenomeAnalysisTK.jar -l INFO -R /projects/USC/production/ref/ensembl_ncbi36/ensembl_hg18_ncbi36_r50.fa -I /projects1/ADNI_2/BWA/Input/s_1_1_sequence.s_1_2_sequence.mapped.sorted.md.realign.dupm.bam -T TableRecalibration --out /projects1/ADNI_2/BWA/Input/s_1_1_sequence.s_1_2_sequence.mapped.rmd.sorted.md.clean.bam -recalFile JLK-227.recal.csv - -default_platform illumina

Pipeline Module:

### 2.4 Post-processing BAM validation

Input: alignment.rmd.sorted.md.clean.bam

Tool:PICARD(ValidateSamFile.jar )

Server Location: /applications/picard_1.38/picard-tools-1.38

Output: file with validation information

Example:

/applications/picard_1.34/picard-tools-1.34/ValidateSamFile.jar INPUT=/projects1/USC/production/data/852603/852603.bam OUTPUT=852603.bam.validation

Pipeline Module:

### 2.5 Covariates calculation after recalibration

Input: alignment.rmd.sorted.md.clean.bam, reference.fasta file

Tool: GATK (GenomeAnalysisTK.jar, -T CountCovariates )

Server Location: /applications/gatk/GATK-2010-01-14/dist

Output: recal_2.csv file (table of the covariates counts)

Example: java -Xmx4g -jar GenomeAnalysisTK.jar -l INFO -R /projects/USC/production/ref/ensembl_ncbi36/ensembl_hg18_ncbi36_r50.fa --DBSNP /applications/GATK/resources/dbsnp_129_b36.rod -I /projects1/ADNI_2/BWA/Input/s_1_1_sequence.s_1_2_sequence.mapped.rmd.sorted.clean.bam -T CountCovariates -cov ReadGroupCovariate -cov QualityScoreCovariate -cov CycleCovariate -cov DinucCovariate -recalFile JLK-227.recal_2.csv --default_platform illumina

Pipeline Module:

### Covariates analysis: plotting score parameters after recalibration

Input: recal_2.csv (table of the covariates counts)

Tool: GATK (AnalyzeCovariates.jar)

Server Location: /applications/gatk/GATK-2010-01-14/dist

Output: plots (.pdf files) and .dat files

Example: java -jar AnalyzeCovariates.jar -recalFile /applications/GATK_source/usc-build/dist/JLK-227.recal_2.csv -outputDir /applications/GATK_source/usc-build/dist/test -resources /applications/GATK_source/Sting/R -Rscript /applications/R-2.11.1/bin/Rscript

Pipeline Module:

### BAM Indexing

Input: alignment.basicQC.final.bam file

Tool: PICARD (BuildBamIndex.jar)

Server Location: /applications/picard_1.38/picard-tools-1.38

Output: alignment.basicQC.final.bam.bai file

Example:

/applications/picard_1.34/picard-tools-1.34/BuildSamIndex.jar INPUT=/projects1/USC/production/data/852603/852603.bam OUTPUT=852603.bam.bai

Pipeline Module:

## Basic stats on reads and alignment (PICARD)

Input: alignment.rmd.sorted.md.clean.bam, reference.fasta file

Tool:PICARD(QualityScoreDistribution.jar,CollectAlignmentSummaryMetrics.jar, CollectGcBiasMetrics.jar, CollectInsertSizeMetrics.jar )

Server Location: /applications/picard_1.38/picard-tools-1.38

Output: pdf and .csv files

Example:

java -jar QualityScoreDistribution.jar INPUT=/projects1/ADNI_2/BWA/Input/s_1_1_sequence.s_1_2_sequence.mapped.rmd.sorted.md.recal.clean.bam OUTPUT=/projects1/ADNI_2/PICARD/JLK-227.qs CHART_OUTPUT=/projects1/ADNI_2/PICARD/JLK-227.pdf VALIDATION_STRINGENCY=SILENT

java -jar CollectInsertSizeMetrics.jar INPUT=/projects1/ADNI_2/BWA/Input/s_1_1_sequence.s_1_2_sequence.mapped.rmd.sorted.md.recal.clean.bam OUTPUT=/projects1/ADNI_2/PICARD/JLK-227.insertmetrics HISTOGRAM_FILE=/projects1/ADNI_2/PICARD/JLK-227.insertsize.pdf VALIDATION_STRINGENCY=SILENT

java -jar MeanQualityByCycle.jar INPUT=/projects1/ADNI_2/BWA/Input/s_1_1_sequence.s_1_2_sequence.mapped.rmd.sorted.md.recal.clean.bam OUTPUT=/projects1/ADNI_2/PICARD/JLK-227.qualitycycle CHART_OUTPUT=/projects1/ADNI_2/PICARD/JLK-227.qualitycycle.pdf VALIDATION_STRINGENCY=SILENT

java -jar CollectAlignmentSummaryMetrics.jar REFERENCE_SEQUENCE=/projects/USC/production/ref/ensembl_ncbi36/ensembl_hg18_ncbi36_r50.fa INPUT=/projects1/ADNI_2/BWA/Input/s_1_1_sequence.s_1_2_sequence.mapped.rmd.sorted.md.recal.clean.bam OUTPUT=/projects1/ADNI_2/PICARD/JLK-227.alignmetrics VALIDATION_STRINGENCY=SILENT

java -jar CollectGcBiasMetrics.jar REFERENCE_SEQUENCE=/projects/USC/production/ref/ensembl_ncbi36/ensembl_hg18_ncbi36_r50.fa INPUT=/projects1/ADNI_2/BWA/Input/s_1_1_sequence.s_1_2_sequence.mapped.rmd.sorted.md.recal.clean.bam OUTPUT=/projects1/ADNI_2/PICARD/JLK-227.gcbiasmetrics CHART=/projects1/ADNI_2/PICARD/JLK-227.gcbias.pdf VALIDATION_STRINGENCY=SILENT

files in yellow has to be open with a pdf viewer outside from the pipeline

Pipeline Module:

## Visualization Tracks production

## 4.1 “Callability” track production for IGV

(general userguide: http://www.broadinstitute.org/gsa/wiki/index.php/Callable_Loci_Walker)

### 4.1.1 Conversion of the reference fasta file to BED

Input: alignment.rmd.sorted.md.clean.bam, reference.fasta file

Tool: GATK (GenomeAnalysisTK.jar, -T CallableLoci)

Server Location: /applications/gatk/GATK-2010-01-14/dist

Output: summary.bed file (for visualization in IGV genome browser)

Example: : nohup java -Xmx4g -jar GenomeAnalysisTK.jar -R /projects/USC/production/ref/ensembl_ncbi36/ensembl_hg18_ncbi36_r50.fa -T CallableLoci -I /projects1/ADNI_2/BWA/Input/s_1_1_sequence.s_1_2_sequence.mapped.rmd.sorted.md.recal.clean.bam -o /projects1/ADNI_2/BWA/Input/GATK/CallableLoci/JLK-227.bases.callable -l INFO -format BED -summary /projects1/ADNI_2/BWA/Input/GATK/CallableLoci/JLK-227.bases.callable.summary

Pipeline Module:

### 4.1.2 BED indexing

Input: summary.bed file

Tool: igvtools (index option)

Server Location: /applications/IGV_tools/IGVTools

Output: summary.bed.idx index file (for visualization in IGV genome browser)

Example: igvtools index /projects1/ADNI/GATK/CallableLoci/JLK-227.bases.callable.bed

Pipeline Module:

## 4.2 Sliding window genomic coverage

Input: alignment.rmd.sorted.md.clean.bam

Tool: igvtools (count)

Server Location: /applications/IGV_tools/IGVTools

Output: .tdf file (for visualization in IGV genome browser)

Example: igvtools count /projects1/ADNI_2/BWA/Input/s_1_1_sequence.s_1_2_sequence.mapped.rmd.sorted.md.recal.bam JLK-227.tdf hg18

Pipeline Module:

## 4.3 Visualization IGV

(not embedded in the pipeline, the user must have downloaded and installed IGV from http://www.broadinstitute.org/igv/)

### 4.3.1 Creation of an IGV project

Input: alignment.rmd.sorted.md.clean.bam, alignment.rmd.sorted.md.recal.clean.bam.bai, summary.bed, summary.bed.idx file, .tdf file

Tool:IGV

Server Location: /applications/IGV_UCI/IGV_1.5.32

Output: summary.bed.idx index file (for visualization in IGV genome browser)

Example: is a GUI application

**FINAL MODULE APPEARANCE**

**Figure 24**: A snapshot of the completed Advanced QC Pipeline workflow

## (2.1a) VARIANT CALLING AND ANNOTATION

## Glossary and Module Overview

“Input”: name of the input file

“Label”: is specified when the name of the input on the pipeline canvas has to be slightly different form the one specified in the Input section to be more clear.

“Tool”: script/program in use

“Server Location”: location on the fgene server

“Output”: name of the output file

## Sequence Variant Analyzer v1.0, for hg18 annotations

# This version of SVA has the CONS that is linked only to ENSEMBL and to older version of dbSNP.

**Goal:** Call variants (SNPs, INDELS) from whole genome alignment data (BAM file) and produce a comprehensive mutation/functional analysis report

**Final outputs:** variant calls in pileup format, .CSV sample variants report from SVA, collection of SVA project data files for visualization with SVA GUI

### 1. PILEUP

Input: subject BAM file, hg18 reference genome FASTA file and FASTA index

Label: SAMTOOLS PILEUP

Tool: samtools pileup

Server location:

/applications/SAMTOOLS/samtools-0.1.16/samtools

Output: subject .pileup file containing variant calls

Example:

$(SAMTOOLS) pileup -f $(REF.FA) -c $(SUBJECT).bam > $(SUBJECT).pileup

Pipeline Module:

### 2. VARIANTS

Input: subject pileup file

Label: SAMTOOLS VARFILTER

Tool: perl, samtools.pl varFilter

Server Location:

/usr/bin/perl /applications/SAMTOOLS/samtools-0.1.16/misc/samtools.pl

Output: .snp_indel file (variants file for SVA data input generation)

Example:

$(PERL) $(SAMTOOLSPL) varFilter -D 200 $(SUBJECT).pileup > $(SUBJECT).snp_indel

Pipeline Module:

### 3. SNV

Input: .snp_indel file

Label: SVA SNV

Tool: perl, snp_filter.pl

Server Location:

/usr/bin/perl /applications/scripts_sva/snp_filter.pl

Output: .samtools file (SNP file for SVA project)

Example:

$(PERL) $(SNP_FILTER) $(SUBJECT).snp_indel > $(SUBJECT).samtools

Pipeline Module:

### 4. INDEL

Input: .snp_indel file

Label: SVA INDEL

Tool: perl, indel_filter.pl

Server Location:

/usr/bin/perl /applications/scripts_sva/indel_filter.pl

Output: .samtoolsindels (INDEL file for SVA project)

Example:

$(PERL) $(INDEL_FILTER) $(SUBJECT).snp_indel > $(SUBJECT).samtoolsindels

Pipeline Module:

### 5. EVENTS (CNV analysis)

Input: pileup file, .samtools file, hg18 reference genome FASTA file, FASTA index, subject ID and gender parameters

Label: ERDS CNV ANALYSIS

Tool: ERDS

Server Location:

/applications/scripts_sva/erds1.01/erds.sh

Output: erds directory with analysis results, .events text file with CNV information (to be used as input for SVA)

Example: ${ERDS.SH} ${SUBJECT} ${GENDER} ${SUBJECT}.pileup ${SUBJECT}.samtools `pwd`/erds_${SUBJECT} ${/PATH/TO/ERDSDIR} ${REFGENOME} ${REFGENOME}.fai

Pipeline Module:

### 6. BCO (genome coverage calculation)

Input: subject .pileup file

Label: PILEUP2BCO

Tool: Java 1.6+, pileup2bco.jar

Server Location:

/applications/scripts_sva/pileup2bco.jar

Output: bco_SUBJECTID directory containing per-chromosome binary-encoded coverage file as input for SVA.

Example: ${JAVA} -jar ${PILEUP2BCO} ${SUBJECT}.pileup `pwd`/bco_${SUBJECT}/${SUBJECT}

Pipeline Module:

### 7. Create GSAP file

NOTE: this step will probably change slightly depending on how we decide to organize the shared repository of SVA-annotated projects on fgene.

Input: SVA data inputs -- .samtools, .samtoolsindels, .snp_indel, ERDS .events file, BCO files, pedinfo.txt file (optional)

Label: Create GSAP

Tool: makeGSAP.sh

Server Location:

/projects/USC/production/bin/SVAtools/bin/makePipelineGSAP.sh

Output: .GSAP file containing paths to data inputs and reference annotation tables

Example: makePipelineGSAP.sh PROJECT_NAME SUBJECT_ID DATA_DIR

Pipeline Module:

### 8. Annotate project

Input: project .GSAP file

Label: SVA HG18 ANNOTATION

Tool: Sequence Variant Analyzer v1.0

Server Location:

/projects/USC/production/bin/SequenceVariantAnalyzer/svacmd.sh

Output: SVA annotated genome -- a set of .CSV tables of variants detected in the sample, a directory of binary-encoded data files used by SVA to display genome mutation analysis results and coverage information in its genome browser.

Example: /usr/bin/nohup $(SVADIR)/svacmd.sh $(RAM_AMOUNT_IN_MB) /PATH/TO/$(SUBJECT).gsap

Pipeline Module:

**FINAL MODULE APPEARANCE**

**Figure 25:** A snapshot of the completed SVA Pipeline workflow .

## SAMTOOLS and ANNOVAR for comprehensive annotation

NOTE: we are defining the usage of command line tools for annotating samples with ANNOVAR, but we will still need to decide on a “flow” of specific annotation steps for each sample, in order to automate it as much as possible within LONI.

**Goal:** call variants (SNPs, INDELs) from whole genome samples and produce comprehensive mutation/functional analysis report using command-line driven ANNOVAR tool and annotations

**Final output:** variant calls (as VCF file), text-based tabular variant reports

### (Pre-pipeline execution) Download annotations and prepare reference databases

NOTE: see <http://www.openbioinformatics.org/annovar/annovar_db.html> for details

Pre-processed reference databases for numerous gene models and reference genome builds are available for ANNOVAR. They should be downloaded to a local directory for future ANNOVAR runs. Custom/user-provided annotations can also be prepared and converted to the ANNOVAR compatible format.

Tool: /applications/ANNOVAR/default/annotate_variation.pl --downdb

General Usage**:** annotate_variation.pl --downdb <table_name> localdb/

#### GENE-BASED ANNOTATION DOWNLOADS

**REFSEQ hg19**

/applications/ANNOVAR/annovar/annotate_variation.pl -downdb -buildver hg19 gene humandb/

**UCSC hg19**

/applications/ANNOVAR/annovar/annotate_variation.pl -downdb -buildver hg19 knownGene humandb/

**ENSEMBL hg19**

/applications/ANNOVAR/annovar/annotate_variation.pl -downdb -buildver hg19 ensGene humandb/

#### REGION-BASED ANNOTATION DOWNLOADS (with any track available on UCSC)

(section 2 <http://www.openbioinformatics.org/annovar/annovar_db.html>, --regionanno argument):

**Most conserved element annotation**

/applications/ANNOVAR/annovar/annotate_variation.pl –downdb mce17way humandb/

/applications/ANNOVAR/annovar/annotate_variation.pl –downdb mce22way humandb/

/applications/ANNOVAR/annovar/annotate_variation.pl –downdb mce44way humandb/

**Transcription factor binding site annotation**

/applications/ANNOVAR/annovar/annotate_variation.pl -downdb tfbs humandb/

**Identify cytogenetic band for genetic variants**

/applications/ANNOVAR/annovar/annotate_variation.pl -downdb band humandb/

**Identify variants located in segmental duplications**

/applications/ANNOVAR/annovar/annotate_variation.pl -downdb segdup humandb/

**Identify previously reported structural variants in DGV (Database of Genomic Variants)**

/applications/ANNOVAR/annovar/annotate_variation.pl -downdb dgv humandb/

**Identify variants reported in previously published GWAS (Genome-wide association studies)**

/applications/ANNOVAR/annovar/annotate_variation.pl -downdb gwascatalog humandb/

**Identify variants in ENCODE annotated regions**

** ENCODE now provides huge amounts of data in Genome Browser tracks that ANNOVAR can annotate against. Some specific examples are shown below, but obviously, there are hundreds of ENCODE annotation tracks that can be used in ANNOVAR (see website and UCSC website).

ENCODE Examples:

**DNASE I hypersensitivity regions**

/applications/ANNOVAR/annovar/annotate_variation.pl -downdb wgEncodeRegDnaseClustered humandb/

**Transcription factor ChIP-Seq regions:**

/applications/ANNOVAR/annovar/annotate_variation.pl -downdb wgEncodeRegTfbsClustered humandb/

#### FILTER-BASED ANNOTATION DOWNLOADS

(see: <http://www.openbioinformatics.org/annovar/annovar_filter.html>

and <http://www.openbioinformatics.org/annovar/annovar_db.html> ):

**dbSNP**

/applications/ANNOVAR/annovar/annotate_variation.pl -downdb -buildver hg18 snp130 humandb/

### ANNOTATION

### 1. Call variants with SAMTOOLS in VCF format

Input: subject .BAM file, reference FASTA file

Tool: samtools pileup

Label: SAMTOOLS PILEUP

Server Location:

/applications/SAMTOOLS/samtools-0.1.16/samtools

Output: raw VCF file (variant call file)

Example: samtools pileup –vcf ref.fa subject.bam > subject.raw.vcf

Pipeline Module:

### 2. Convert VCF to ANNOVAR format

(see: <http://www.openbioinformatics.org/annovar/annovar_input.html#pileup> for more information on quality filtering parameters)

Input: subject.raw.vcf

Tool: convert2annovar.pl

Server Location:

/applications/ANNOVAR/default/convert2annovar.pl

Output: subject.annovar

Example:

/applications/ANNOVAR/default/convert2annovar.pl subject.raw.vcf [PARAMS] > subject.annovar

Pipeline Module:

### 3. ANNOTATE

(see: <http://www.openbioinformatics.org/annovar/annovar_gene.html> )

Input: subject.annovar

Tool: annotate_variation.pl

Label: ANNOVAR_ANNOTATE

Server Location:

/applications/ANNOVAR/default/annotate_variation.pl

Output:

subject.annovar.human.variant_function -- from website: “contains annotation for all variants”

subject.annovar.human.exonic_variant_function -- from website: “contains the amino acid changes as a result of the exonic variant. The exact format of the output below may change slightly between different versions of ANNOVAR.”

Examples:/applications/ANNOVAR/default/annotate_variation.pl [PARAMS] subject.annovar localdb/

Pipeline Module:

**USAGE PARAMETERS**

**Switch from one gene model to another**

--dbtype refgene (default)

--dbtype knowngene (UCSC)

--dbtype ensgene (ensembl)

**Switch between genome builds**

--buildver hg18 (default)

--buildver hg19

#### Gene-based annotation

**REFSEQ hg18 (default):**

--geneanno --buildver hg18 -dbtype gene

**REFSEQ hg19:**

--buildver hg19

**UCSC hg18:**

--dbtype knowngene

**UCSC hg19**:

--dbtype knowngene --buildver hg19

**ENSEMBL hg18:**

--dbtype ensgene

**ENSEMBL hg19**:

--dbtype ensgene --buildver hg19

#### Region-based annotation (with any track available in UCSC)

Input: subject.annovar

Tool: annotate_variation.pl

Label: ANNOVAR_ANNOTATE_REGION

Server Location:

/applications/ANNOVAR/default/annotate_variation.pl

Output: TBD

Example: TBD

#### Filter-based annotation

Input: subject.annovar

Tool: annotate_variation.pl

Label: ANNOVAR_ANNOTATE_FILTER

Server Location:

/applications/ANNOVAR/default/annotate_variation.pl

Output:

subject.annovar.hg18_snpXYZ_filtered – SNPs not detected in dbSNP

subject.annovar.hg18_snpXYZ_dropped – SNPs contained in dbSNP, with rs numbers

Example:

**USAGE:**

/applications/ANNOVAR/default/annotate_variation.pl –filter [PARAMS] subject.annovar localdb/

dbSNP

--dbsnp VERSION

**FINAL MODULE APPEARANCE**

**Figure 26:** A snapshot of the completed SAMTOOLS-ANNOVAR Pipeline workflow.

## UnifiedGenotyperV2 and ANNOVAR FOR COMPREHENSIVE ANNOTATION

**Goal:** call variants (SNPs, INDELs) from whole genome samples and produce comprehensive mutation/functional analysis report using command-line driven ANNOVAR tool and annotations

**Final output:** variant calls (as VCF file), text-based tabular variant reports

## Variant calling with GATK

(Unified genotyper tool: http://www.broadinstitute.org/gsa/gatkdocs/release/org_broadinstitute_sting_gatk_walkers_genotyper_UnifiedGenotyper.html#--dbsnp)

Input: .bam file (better if after basic and advanced QC procedures like alignment.rmd.sorted.md.clean.bam)

Tool: GATK (UnifiedGenotyperV2)

Server Location: /applications/GATK_source/usc-build/dist

Output: .vcf file

Example (to output ONLY SNPs): nohup java -jar GenomeAnalysisTK.jar -l INFO -R /projects/USC/production/ref/ensembl_ncbi36/ensembl_hg18_ncbi36_r50.fa -T UnifiedGenotyperV2 -I /projects1/ADNI_2_USC/JLK-227/JLK-227.bam -o /projects1/ADNI_2_USC/GATK/JLK-227_dbsnp130.geno --DBSNP /applications/GATK/resources/dbsnp_129_b36.rod --assume_single_sample_reads JLK-227 --platform SOLEXA -all_bases -stand_call_conf 30.0*

nohup java -jar GenomeAnalysisTK.jar -l INFO -R /projects/USC/production/ref/ensembl_ncbi36/ensembl_hg18_ncbi36_r50.fa -T UnifiedGenotyperV2 -I /projects1/ADNI_2_USC/JLK-227/JLK-227.bam -o /projects1/test_pipeline/NEW_ORGANIZATION/GENOTYPING_MAP_PED/test.geno --DBSNP /projects1/test_pipeline/NEW_ORGANIZATION/GENOTYPING_MAP_PED/00-All.vcf --assume_single_sample_reads JLK-227 --platform SOLEXA -all_bases

Example (to output only sites confident with the reference genome): nohup java -jar GenomeAnalysisTK.jar -l INFO -R /projects/USC/production/ref/ensembl_ncbi36/ensembl_hg18_ncbi36_r50.fa -T UnifiedGenotyperV2 -I /projects1/ADNI_2_USC/JLK-227/JLK-227.bam -o /projects1/ADNI_2_USC/GATK/JLK-227_dbsnp130.geno --DBSNP /applications/GATK/resources/dbsnp_129_b36.rod --assume_single_sample_reads JLK-227 --platform SOLEXA -all_bases -stand_call_conf 30.0* --output_mode EMIT_ALL_CONFIDENT_SITES

Example (to output the calls over ALL sites..useful to create similar matrix between different subjects): nohup java -jar GenomeAnalysisTK.jar -l INFO -R /projects/USC/production/ref/ensembl_ncbi36/ensembl_hg18_ncbi36_r50.fa -T UnifiedGenotyperV2 -I /projects1/ADNI_2_USC/JLK-227/JLK-227.bam -o /projects1/ADNI_2_USC/GATK/JLK-227_dbsnp130.geno --DBSNP /applications/GATK/resources/dbsnp_129_b36.rod --assume_single_sample_reads JLK-227 --platform SOLEXA -all_bases -stand_call_conf 30.0* --output_mode EMIT_ALL_SITES

Some of the most important flags from GATK website:

**--genotyping_mode / -gt_mode (GENOTYPING_MODE with default value DISCOVERY)**

Should we output confident genotypes (i.e. including ref calls) or just the variants?.

The --genotyping_mode argument specifies how to determine the alternate allele to use for genotyping and is an enumerated type (GENOTYPING_MODE), which can have one of the following values:

DISCOVERY

the default; the Unified Genotyper will choose the most likely alternate allele

GENOTYPE_GIVEN_ALLELES

only the alleles passed in from a VCF rod bound to the -alleles argument will be used for genotyping. Note that if we combine this option with the export all SITES we can have PED identical for different patients (i.e. what we want for case-control analyses)

**--dbsnp / -D (RodBinding[VariantContext] with default value none)**

dbSNP file. rsIDs from this file are used to populate the ID column of the output. Also, the DB INFO flag will be set when appropriate. dbSNP is not used in any way for the calculations themselves. --dbsnp binds reference ordered data. This argument supports ROD files of the following types: [VCF](http://www.broadinstitute.org/gsa/gatkdocs/release/org_broadinstitute_sting_utils_codecs_vcf_VCFCodec.html), [VCF3](http://www.broadinstitute.org/gsa/gatkdocs/release/org_broadinstitute_sting_utils_codecs_vcf_VCF3Codec.html)

To find the most current ROD files: <http://www.broadinstitute.org/gsa/wiki/index.php/GSA_FTP_Server> or <ftp://ftp.ncbi.nih.gov/snp/>: ftp://ftp.ncbi.nih.gov/snp/organisms/human_9606/VCF/v4.0/

**--debug_file / -debug_file (PrintStream)**

***Example generic command for multi-sample SNP calling***: java -jar GenomeAnalysisTK.jar \ -R resources/Homo_sapiens_assembly18.fasta \ -T UnifiedGenotyper \ -I sample1.bam [-I sample2.bam ...] \ --dbsnp dbSNP.vcf \ -o snps.raw.vcf \ -stand_call_conf [50.0] \ -stand_emit_conf 10.0 \ -dcov [50] \ [-L targets.interval_list]

The above command will call all of the samples in your provided BAM files [-I arguments] together and produce a VCF file with sites and genotypes for all samples. The easiest way to get the dbSNP file is from the GATK resource bundle. Several arguments have parameters that should be chosen based on the average coverage per sample in your data.

***Example command for generating calls at all sites:*** java -jar /path/to/GenomeAnalysisTK.jar \ -l INFO \ -R resources/Homo_sapiens_assembly18.fasta \ -T UnifiedGenotyper \ -I /DCC/ftp/pilot_data/data/NA12878/alignment/NA12878.SLX.maq.SRP000031.2009_08.bam \ -o my.vcf \ --output_mode EMIT_ALL_SITES

**--alleles / -alleles (RodBinding[VariantContext] with default value none)**

The set of alleles at which to genotype when in GENOTYPE_MODE = GENOTYPE_GIVEN_ALLELES. When the UnifiedGenotyper is put into GENOTYPE_GIVEN_ALLELES mode it will genotype the samples using only the alleles provide in this rod binding --alleles binds reference ordered data. This argument supports ROD files of the following types: [VCF](http://www.broadinstitute.org/gsa/gatkdocs/release/org_broadinstitute_sting_utils_codecs_vcf_VCFCodec.html), [VCF3](http://www.broadinstitute.org/gsa/gatkdocs/release/org_broadinstitute_sting_utils_codecs_vcf_VCF3Codec.html).

**--output_mode / -out_mode (OUTPUT_MODE with default value EMIT_VARIANTS_ONLY)**

Should we output confident genotypes (i.e. including ref calls) or just the variants?.
The --output_mode argument is an enumerated type (OUTPUT_MODE), which can have one of the following values:

EMIT_VARIANTS_ONLY

the default

EMIT_ALL_CONFIDENT_SITES

include confident reference sites

EMIT_ALL_SITES

any callable site regardless of confidence

**FINAL MODULE APPEARANCE**

**Figure 27:** A snapshot of the completed UnifiedGenotyperV2-ANNOVAR Pipeline workflow

# (2.1b) CNVs calling modules

# GLOSSARY and MODULE OVERVIEW

“Input”: name of the input file

“Label”: is specified when the name of the input on the pipeline canvas has to be slightly different form the one specified in the Input section to be more clear.

“Tool”: script/program in use

“Server Location”: location on the fgene server

“Output”: name of the output file

# MODULE DESCRIPTION

**GOAL**: call CNVs starting from raw reads (FASTQ format)

**FINAL OUTPUT**: files with CNV calls

## ERDS/SVA path (DOC)

(see “Sequence Variant Analyzer v1.0, for hg18 annotations” pipeline, for “Sequence Variant Analyzer v1.1, for has not been released yet a compatible ERDS version)

Input: alignment.rmd.sorted.md.recal.clean.bam (= output STEP#)

Tool: erds

Server Location: /applications/scripts_sva/erds1.01/erds.sh

Output: .gsap file (visualization of CNVs in Sequence Variant Analyzer). From the GUI is possible to export the large variants in a .csv file.

Example:

Pipeline Module:

This module is embedded into the Sequence Variant Analyzer v1.0 pipeline.

## BOWTIE/CNVer/SAVANT path (DOC+PEM)

(PREREQUISITE: download the companion package from the CNVer website http://compbio.cs.toronto.edu/CNVer/. It allows using CNVer on alignments performed against the UCSC ref genome. See notes on ensemble alignments.)

## BOWTIE alignment

### Build bowtie index for the reference genome

Input: ref.fa reference files (preferentially the UCSC fasta reference genome, chr1-22,X,Y. See notes if using ensemble genome)

Tool: bowtie (bowtie-build command)

Server Location: /applications/rseqtools/example_dataset/bowtie-0.12.7

Output: series of .ebwt files

Example: bowtie-build ${CD}/human_genome2.fa ucsc_hg18_new_bowtie > \

ucsc_hg18_new_bowtie.log

Pipeline Module:

### Bowtie alignment with SAM production

Input: sequence.fastq file (SANGER FORMAT, output of STEP#1, I)

Label: Illumina reads sequence.fastq files

Tool: bowtie

Server Location: /applications/BOWTIE/bowtie-0.12.7

Output: alignment.sam

Example: bowtie ${CD}/ucsc_hg18_new_bowtie -1 ${CD}/941408_fwd.fastq -2

${CD}/941408_rev.fastq -v 3 -a -m 600 --best --strata --sam

${CD}/941408_bduc_ucsc_hg18_new.sam >

${CD}/941408_bduc_ucsc_hg18_new.log

Pipeline Module:

### SAM2BAM conversion

Input: alignment.sam

Tool:picard (SamFormatConverter.jar)

Server Location: /applications/picard_1.38/picard-tools-1.38

Output: alignment.bam

Example: -jar /apps/picard/1.45/bin/SamFormatConverter.jar INPUT= ${CD}/941408_bduc_ucsc_hg18_new.sam \

OUTPUT=${CD}/941408_bduc_ucsc_hg18_new.bam

Pipeline Module:

## CNVer

### CNVer call

Input: alignment.bam

Tool: CNVer (cnver.pl)

Server Location: /applications/CNVer/cnver-0.8.1/src

Output: .cnv files

Example: cnver.pl --map_list /projects2/CNVer_0.8.1_testing/map_list.txt --ref_folder /applications/CNVer/cnver-0.8.1/hg18comp --work_dir /projects2/CNVer_0.8.1_testing --read_len 101 --mean_insert 175 --stdev_insert 25 --min_mps 3

Pipeline Module:

### Sort .bam

Input: alignment.bam file

Tool: samtools (sort option)

Server Location: /applications/samtools-0.1.7_x86_64-linux

Output: alignment.sorted.bam file

Example: /applications/samtools-0.1.7_x86_64-linux/samtools sort /projects/pipelineCache/pipeline/2011January27_15h51m34s061ms/SamToolsRemoveDuplicates_1.OutputNo-DuplicatesBAMfile-1.bam /projects/pipelineCache/pipeline/2011January27_15h51m34s061ms/SamToolsSort_1.OutputSortedBAMfile-1.bam

Pipeline Module:

### Indexing the .bam file

Input: alignment.sorted.md.bam file

Tool: samtools (index option)

Server Location: /applications/samtools-0.1.7_x86_64-linux

Output: alignment.sorted.md.bam.bai file

Example: /applications/samtools-0.1.7_x86_64-linux/samtools index /projects/pipelineCache/pipeline/2011January27_15h51m34s061ms/SamToolsCamldMDtag_1.OutputNo-DuplicatesBAMfile-1.bam

Pipeline Module:

### Visualization

(not implemented in the pipeline, but must be followed to have the final visualization of the CNVs)

#### BAM sorting

Input: alignment.rmd.bam file

Tool: samtools (sort option)

Server Location: /applications/samtools-0.1.7_x86_64-linux

Output: alignment.rmd.sorted.bam file

Example: /applications/samtools-0.1.7_x86_64-linux/samtools sort /projects/pipelineCache/pipeline/2011January27_15h51m34s061ms/SamToolsRemoveDuplicates_1.OutputNo-DuplicatesBAMfile-1.bam /projects/pipelineCache/pipeline/2011January27_15h51m34s061ms/SamToolsSort_1.OutputSortedBAMfile-1.bam

Pipeline Module:

#### Coverage track production

Input: alignment.bam

Tool: SAVANT genome browser (GUI)

Server Location: /applications/SAVANT_gb_updated

Output: alignment.genome.cov.bam file

Example: through SAVANT GUI

#### Formatting the ref.fa file in a ref.fa.savant

Input: ref.fa file

Tool: SAVANT genome browser (GUI)

Server Location: /applications/SAVANT_gb_updated

Output: ref.fa.savant project

Example: through SAVANT GUI

#### Formatting the cnv file in cnv.bed

Input: .cnv file

Tool: SAVANT genome browser (GUI)

Server Location: /applications/SAVANT_gb_updated

Output: .cnv.bed

Example: through SAVANT GUI

#### Visualization

Input: alignment.sorted.bam, alignment, genome.cov.bam, .cnv.bed files

Tool: SAVANT genome browser

Server Location: /applications/SAVANT_gb_updated

Output: saved in a .savant project

Example: through SAVANT GUI

**FINAL MODULE APPEARANCE**

**Figure 28** A snapshot of the completed CNVer Pipeline workflow. The insert image illustrates the final output result that then undergoes to visualization with SAVANT genome browser.

**ENSEMBL ALIGNMENTS NOTES**

**PREREQUISITE: perform the alignments on a 1-22,X,Y ensemble reference genome. AVOID the contigs!**

1. **Conversion chr1-22 (UCSC) to 1-22 (ENSEMBL).**
   1. In the hg18comp folder remove chr from allchr.txt, autosomes.txt.
   2. In the folders inside hg18comp (contig_breaks_folder, fasta_files_folder, repeat_regions_folder, self_alignments_folder) change the name of the files and also the content of the file accordingly chr1-22 (UCSC) to 1-22 (ENSEMBL).

## CNVseq path

## Hits file production

Input: alignment.bam

Tool: samtools (view option)

Server Location: /applications/samtools-0.1.7_x86_64-linux

Output: .hits file

Example: samtools view -F 4 my.bam | perl -lane 'print "$F[2]\t$F[3]"' > my.hits

Pipeline Module:

## CNV call with CNVseq

Input: .hits file

Tool: CNVseq+R

Server Location: /applications/CNVseq/cnv-seq

Output: .hits.cnv file

Example: ./cnv-seq.pl --test SMS-135.hits --ref JAM-230.hits --genome human

In R:

> library(proto)

> library(grid)

> library(cnv)

> data <- read.delim("JLK-227.hits-vs-JAM-230.hits.log2-0.6.pvalue-0.001.minw-4.cnv") #upload the .cnv file

> cnv.summary(data) # produce a description of the dataset like that:

CNV percentage in genome: 0.8%

CNV nucleotide content: 24516307

CNV count: 403

Mean size: 60835

Median size: 135865

Max Size: 503201

Min Size: 20129

>plot.cnv(data, chromosome=2, from=140036061, to=144238634) # to plot a specific region so in this case you can insert the interval you want and see the log R ratio OR check the cnv.print file and look inside that if your region is among the significant ones.

>plot.cnv(data, CNV=4, upstream=4e+6, downstream=4e+6) #to plot a CNV (when you do cnv.print(data) look at the CNVid and them choose what CNV to be plotted)

Pipeline Module:

NOTE:

- **What a user might want to do with the plotting (see OUTPUT PLOT)?**

(1)Automatically export ALL the pdf plots of all the CNVs (so this step may be connected directly to the CNVseq. I guess would be great to put a parameter that if checked allows this automatic production:

plot.cnv.all <- function (data, chrom.gap = 2e+07,

colour = 5, title = WG plots, ylim = c(-2,2),

xlabel = "Chromosome")

(2) Automatically export ALL the pdf plots of all the CNVs in one chromosome by time (so this step may be connected directly to the CNVseq. I guess would be great to put a parameter that if checked allows this automatic production (with the possibility to choose the chromosome):

plot.cnv.chr <- function (data, chromosome = the number of the chromosome, from = NA(beginning coordinate), to = (ending coordinate), title = chromosome x,

ylim = c(-4, 4), glim = c(-2, 2),

xlabel = "Position (bp)")

(3) In the vast majority of the cases, the user will download the cnv file produced by CNVseq, will examine it and choose some events that are more interesting. In this case what I figure in my mind is the possibility to check a parameter that says “by now stop the process to CNVseq”. Once the user has downloaded and examined the .cnv files and has x regions to visualize, he can go back to the same module (without having restarted it) and export plots:

By genomic region: >plot.cnv(data, chromosome=2, from=140036061, to=144238634) # to plot a specific region so in this case you can insert the interval you want and see the log R ratio

By CNVid: >plot.cnv(data, CNV=4 (this the is found by the user in the cnv file, upstream=4e+6, downstream=4e+6)

- **What a user might want to do with the output description file (see OUTPUT DATA DESCRIPTION)?**

This step is ok.

**FINAL MODULE APPEARANCE**

**Figure 29** A snapshot of the completed CNVseq Pipeline workflow. The insert image illustrates the final output result: the CNVcalls file, the plots (whole genome, by chromosome or by region) and the general CNVs counts.
