# Supplementary material for: Next Generation Sequence Analysis and Computational Genomics Using Graphical Pipeline Workflows
Source: Genes (Basel). 2012 Aug 30;3(3):545–75. doi: 10.3390/genes3030545 (PMC3490498; doi:10.3390/genes3030545)
Supplement: Supplementary File 1 — ZIP-Document (ZIP, 7447 KB) [file genes-03-00545-s001.zip › Supplementary/TableS1.doc]

| **File format** | **File type** | **Description** | **Workflows** | **Example** |
| --- | --- | --- | --- | --- |
| **.fasta** | text | In [bioinformatics](http://en.wikipedia.org/wiki/Bioinformatics), FASTA format is a text-based [format](http://en.wikipedia.org/wiki/File_format) for representing either [nucleotide sequences](http://en.wikipedia.org/wiki/Nucleotide_sequence) or [peptide sequences](http://en.wikipedia.org/wiki/Peptide_sequence), in which [base pairs](http://en.wikipedia.org/wiki/Base_pair) or [amino acids](http://en.wikipedia.org/wiki/Amino_acid) are represented using single-letter codes. See http://en.wikipedia.org/wiki/FASTA_format | (1.1) Alignment; (1.2)  De novo assembly; (1.3) Basic QC; (1.4) Advanced QC; (2.1a) Variant calling and annotation; (2.1b) CNVs calling | >hg18_dna range=chr6:135570000-136570000 5'pad=0 3'pad=0 strand=+ repeatMasking=none  GTACTTTATATAATCTATAATTTTTGTAATTTGTACTTATAATAAATCAA  ATATTAAGTGGGATTTTAGCCAAACCATCTAGACATTTTTGTCATAATGT  TTTTATTAAAACAGCAAAATAGGAGTTTCAAAATATTTTAAAATTTGAAT  ACTTCTAAAATGTAAAATGTTTGCAACTGGTGTAATGCAGTTGATTGTCT  ATCCCTCTAAACTGTCTAAATAACTTAGGTTCAAGAACAGTTTATTGAAG  AACATAGGAACTGCGCAATTCTGTGTTTTATCATTTCAGTTTTAATCACC  CCGGCTTGATTATGGCAACAACATTTTAGACAAGCAATTATTACTATTTT  TGAGGCCCATATACTGGGCCATTATGGTTTTGCTTCTAACGTACATATAA  ATGCACATGCAATATATATAAATTATGTTTGCTGTTTTAAAATTAGAAAT |
| **.bfa** | binary | Binary version of the fasta file used by MAQ | (1.1) Alignment, (1.2) De novo assembly. |  |
| **.fasta.savant** | text | Reference .fsta file in a format compatible with SAVANT genomic visualization tool. | (2.1b) CNVs calling |  |
| **.fai** | text | Index of the .fasta file | (1.3) Basic QC | 1 247249719 37 60 61  2 242951149 251370622 60 61  3 199501827 498370994 60 61  4 191273063 701197889 60 61  5 180857866 895658874 60 61  6 170899992 1079531075 60 61  7 158821424 1253279438 60 61  8 146274826 1414747923 60 61  9 140273252 1563460700 60 61  10 135374737 1706071879 60 61  11 134452384 1843702901 60 61  12 132349534 1980396198 60 61  13 114142980 2114951597 60 61  14 106368585 2230996999 60 61  15 100338915 2339138433 60 61  16 88827254 2441149702 60 61  17 78774742 2531457449 60 61  18 76117153 2611545142 60 61  19 63811651 2688930953 60 61  20 62435964 2753806170 60 61  21 46944323 2817282772 60 61  22 49691432 2865009539 60 61  X 154913754 2915529199 60 61  Y 57772954 3073024891 60 61  MT 16571 3131760763 60 61 |
| **.fastq** | text | FASTQ format is a text-based [format](http://en.wikipedia.org/wiki/File_format) for storing both a biological sequence (usually [nucleotide sequence](http://en.wikipedia.org/wiki/Nucleotide_sequence)) and its corresponding quality scores. Both the sequence letter and quality score are encoded with a single [ASCII](http://en.wikipedia.org/wiki/ASCII) character for brevity. It was originally developed at the [Wellcome Trust Sanger Institute](http://en.wikipedia.org/wiki/Wellcome_Trust_Sanger_Institute) to bundle a [FASTA](http://en.wikipedia.org/wiki/FASTA_format) sequence and its quality data, but has recently become the de facto standard for storing the output of high throughput sequencing instruments. Sanger format can encode a [Phred quality score](http://en.wikipedia.org/wiki/Phred_quality_score) from 0 to 93 using [ASCII](http://en.wikipedia.org/wiki/ASCII) 33 to 126 (although in raw read data the Phred quality score rarely exceeds 60, higher scores are possible in assemblies or read maps (see: http://en.wikipedia.org/wiki/FASTQ_format) | (1.1) Alignment; (1.2) De novo assembly; (2.1b) CNVs calling | @SEQ_ID GATTTGGGGTTCAAAGCAGTATCGATCAAATAGTAAATCCATTTGTTCAACTCACAGTTT + !''*((((***+))%%%++)(%%%%).1***-+*''))**55CCF>>>>>>CCCCCCC65 |
| **.bfq** | binary | Binary verison of the fastq reads file used by MAQ. | (1.1) Alignment; (1.2) De novo assembly |  |
| **_sequence.txt** | text | Indistinguishable version of the FASTQ format incompatible with the softwares used for alignement/de novo assembly/CNV calling. It needs a co version to the canonical .fastq format. It differs form the fastq format for the Phred quality score ancoding (see http://en.wikipedia.org/wiki/FASTQ_format) | (1.1) Alignment; (1.2) De novo assembly; (2.1b) CNVs calling | @HWI-EAS209_0006_FC706VJ:5:58:5894:21141#ATCACG/1 TTAATTGGTAAATAAATCTCCTAATAGCTTAGATNTTACCTTNNNNNNNNNNTAGTTTCTTGAGATTTGTTGGGGGAGACATTTTTGTGATTGCCTTGAT +HWI-EAS209_0006_FC706VJ:5:58:5894:21141#ATCACG/1 efcfffffcfeefffcffffffddf`feed]`]_Ba_^__[YBBBBBBBBBBRTT\]][]dddd`ddd^dddadd^BBBBBBBBBBBBBBBBBBBBBBBB |
| **.map** | binary | Alignment result of MAQ. | (1.1) Alignment; (1.2) De novo assembly |  |
| **.sam** | text | SAM (Sequence Alignment/Map) format is a generic format for storing large nucleotide sequence alignments. It is a TAB-delimited text format consisting of a header section, which is optional, and an alignment section (see: http://samtools.sourceforge.net/SAM1.pdf) | (1.1) Alignment; (1.2)  De novo assembly; (1.3) Basic QC | @SQ SN:1 LN:247249719  @SQ SN:2 LN:242951149  @SQ SN:3 LN:199501827  @SQ SN:4 LN:191273063  @SQ SN:5 LN:180857866  @SQ SN:6 LN:170899992  @SQ SN:7 LN:158821424  @SQ SN:8 LN:146274826  @SQ SN:9 LN:140273252  @SQ SN:10 LN:135374737  @SQ SN:11 LN:134452384  @SQ SN:12 LN:132349534  @SQ SN:13 LN:114142980  @SQ SN:14 LN:106368585  @SQ SN:15 LN:100338915  @SQ SN:16 LN:88827254  @SQ SN:17 LN:78774742  @SQ SN:18 LN:76117153  @SQ SN:19 LN:63811651  @SQ SN:20 LN:62435964  @SQ SN:21 LN:46944323  @SQ SN:22 LN:49691432  @SQ SN:X LN:154913754  @SQ SN:Y LN:57772954  @SQ SN:MT LN:16571  @RG ID:6191BAAXX-1 SM:852603  @PG ID:bwa PN:bwa VN:0.5.9-r16  GA-BETA1544-1_0001:1:1:1019:8453#0 97 X 49199586 0 101M 1 156551401 0 NTTCCCACCTATGAGTGAGAATATGCGGTGTTTGGTTTTTTGTTCTTGCAATAGTTTACTGAGAATGATGATTTCCAGTTTCATCCATGTCCCTACAAAGG #''*',*++'AAAAAAAAAAAAAAA8::57:3:::AAA7A5A777A7A77::7:8AA7AAAAA-AAA8AA8665744:24334:::424-:83:8AAA### RG:Z:6191BAAXX-1 XT:A:R NM:i:1 SM:i:0 AM:i:0 X0:i:17 X1:i:201 XM:i:1 XO:i:0 XG:i:0 MD:Z:0G100 |
| **.sai** | binary | Index of the .sam file | (1.1) Alignment; (1.2)  De novo assembly; (1.3) Basic QC |  |
| **.bam** | binary | BAM is compressed in the BGZF format. BAM is the compressed binary version of the [Sequence Alignment/Map (SAM)](http://samtools.sourceforge.net/#_blank) format, a compact and index-able representation of nucleotide sequence alignments. Many [next-generation sequencing and analysis tools](http://samtools.sourceforge.net/swlist.shtml#_blank) work with BAM. | (1.3) Basic QC; (1.4) Advanced QC; (2.1a) Variant calling and annotation; (2.1b) CNVs calling |  |
| **cov.bam** | binary | .bam file in a format compatible with SAVANT genomic visualization tool. | (2.1b) CNVs calling |  |
| **.bai** | binary | Index of the .bam file | Basic QC; (1.4) Advanced QC; (2.1a) Variant calling and annotation; (2.1b) CNVs calling |  |
| **.ebwt** | binary | Bowtie index from a set of DNA sequences (usually the reference sequence). bowtie-build outputs a set of 6 files with suffixes .1.ebwt, .2.ebwt, .3.ebwt, .4.ebwt, .rev.1.ebwt, and .rev.2.ebwt. These files together constitute the index: they are all that is needed to align reads to that reference. The original sequence files are no longer used by Bowtie once the index is built. | (1.1) Alignment; (1.2) De novo assembly; (2.1b) CNVs calling |  |
| **.tdf** | binary | Sliding windows genomic coverage track. | (1.4) Advanced QC |  |
| **.pileup** | binary | It describes the base-pair information at each chromosomal position. This format facilitates SNP/indel calling and brief alignment viewing by eyes. For a complete description of the format see:http://samtools.sourceforge.net/pileup.shtml. .mpileup in now the preferred format. | (2.1a) Variant calling and annotation | seq1 272 T 24  ,.$.....,,.,.,...,,,.,..^+. <<<+;<<<<<<<<<<<=<;<;7<& seq1 273 T 23  ,.....,,.,.,...,,,.,..A <<<;<<<<<<<<<3<=<<<;<<+ seq1 274 T 23   ,.$....,,.,.,...,,,.,...    7<7;<;<<<<<<<<<=<;<;<<6 seq1 275 A 23  ,$....,,.,.,...,,,.,...^l.  <+;9*<<<<<<<<<=<<:;<<<< seq1 276 G 22  ...T,,.,.,...,,,.,....  33;+<<7=7<<7<&<<1;<<6< seq1 277 T 22  ....,,.,.,.C.,,,.,..G.  +7<;<<<<<<<&<=<<:;<<&< seq1 278 G 23  ....,,.,.,...,,,.,....^k.   %38*<<;<7<<7<=<<<;<<<<< seq1 279 C 23  A..T,,.,.,...,,,.,..... ;75&<<<<<<<<<=<<<9<<:<< |
| **.mpileup** | binary | For a complete description of the format see http://samtools.sourceforge.net/mpileup.shtml. | (2.1a) Variant calling and annotation |  |
| **.vcf** | text | The [Variant Call Format](http://vcftools.sourceforge.net/specs.html) (VCF) is the emerging standard for storing variant data. Originally designed for SNPs and short INDELs, it also works for structural variations. For a complete description of the format see http://samtools.sourceforge.net/mpileup.shtml. | (2.1a) Variant calling and annotation | ##fileformat=VCFv4.0 ##fileDate=20090805 ##source=myImputationProgramV3.1 ##reference=1000GenomesPilot-NCBI36 ##phasing=partial ##INFO=<ID=NS,Number=1,Type=Integer,Description="Number of Samples With Data"> ##INFO=<ID=DP,Number=1,Type=Integer,Description="Total Depth"> ##INFO=<ID=AF,Number=.,Type=Float,Description="Allele Frequency"> ##INFO=<ID=AA,Number=1,Type=String,Description="Ancestral Allele"> ##INFO=<ID=DB,Number=0,Type=Flag,Description="dbSNP membership, build 129"> ##INFO=<ID=H2,Number=0,Type=Flag,Description="HapMap2 membership"> ##FILTER=<ID=q10,Description="Quality below 10"> ##FILTER=<ID=s50,Description="Less than 50% of samples have data"> ##FORMAT=<ID=GT,Number=1,Type=String,Description="Genotype"> ##FORMAT=<ID=GQ,Number=1,Type=Integer,Description="Genotype Quality"> ##FORMAT=<ID=DP,Number=1,Type=Integer,Description="Read Depth"> ##FORMAT=<ID=HQ,Number=2,Type=Integer,Description="Haplotype Quality"> #CHROM POS ID REF ALT QUAL FILTER INFO FORMAT NA00001 NA00002 NA00003 20 14370 rs6054257 G A 29 PASS NS=3;DP=14;AF=0.5;DB;H2 GT:GQ:DP:HQ 0\|0:48:1:51,51 1\|0:48:8:51,51 1/1:43:5:.,. 20 17330 . T A 3 q10 NS=3;DP=11;AF=0.017 GT:GQ:DP:HQ 0\|0:49:3:58,50 0\|1:3:5:65,3 0/0:41:3 20 1110696 rs6040355 A G,T 67 PASS NS=2;DP=10;AF=0.333,0.667;AA=T;DB GT:GQ:DP:HQ 1\|2:21:6:23,27 2\|1:2:0:18,2 2/2:35:4 20 1230237 . T . 47 PASS NS=3;DP=13;AA=T GT:GQ:DP:HQ 0\|0:54:7:56,60 0\|0:48:4:51,51 0/0:61:2 20 1234567 microsat1 GTCT G,GTACT 50 PASS NS=3;DP=9;AA=G GT:GQ:DP 0/1:35:4 0/2:17:2 1/1:40:3 |
| **.gff** | text | GFF (General Feature Format) lines are based on the GFF standard file format. GFF lines have nine required fields that must be tab-separated. If the fields are separated by spaces instead of tabs, the track will not display correctly. For more information on GFF format, refer to [www.sanger.ac.uk/resources/software/gff](http://www.sanger.ac.uk/resources/software/gff). Formats for feature-by-feature data | (2.1a) Variant calling and annotation | browser hide all  track name="chr11" visibility=2  chr11 MRF feature 46772115 46772161 . - . TG5  chr11 MRF feature 46772668 46772695 . - . TG5  chr11 MRF feature 118521207 118521252 . + . TG21  chr11 MRF feature 118526315 118526343 . + . TG21 |
| **.bed** | text | The BED format is used to represent contiguous genomic regions. Formats for feature-by-feature data (coordinates 0-based). See [http://genome.ucsc.edu/FAQ/FAQformat.html#format3](http://genome.ucsc.edu/FAQ/FAQformat.html#format3) | (1.4) Advanced QC | chr1 1000 5000  chr3 500 600  chrX 4000 4250 |
| **.gtf3** | text | GTF (Gene Transfer Format) is a refinement to GFF that tightens the specification.  This format can be used to conveniently store the gene annotation data. Here is an example. The definition of the GFF3 format can be found here: [www.sequenceontology.org/gff3.shtml](http://www.sequenceontology.org/gff3.shtml) | (2.1a) Variant calling and annotation | 0 ##gff-version 3 1 ##sequence-region ctg123 1 1497228 2 ctg123 . gene 1000 9000 . + . ID=gene00001;Name=EDEN 3 ctg123 . TF_binding_site 1000 1012 . + . ID=tfbs00001;Parent=gene00001 4 ctg123 . mRNA 1050 9000 . + . ID=mRNA00001;Parent=gene00001;Name=EDEN.1 5 ctg123 . mRNA 1050 9000 . + . ID=mRNA00002;Parent=gene00001;Name=EDEN.2 6 ctg123 . mRNA 1300 9000 . + . ID=mRNA00003;Parent=gene00001;Name=EDEN.3 7 ctg123 . exon 1300 1500 . + . ID=exon00001;Parent=mRNA00003 8 ctg123 . exon 1050 1500 . + . ID=exon00002;Parent=mRNA00001,mRNA00002 9 ctg123 . exon 3000 3902 . + . ID=exon00003;Parent=mRNA00001,mRNA00003 10 ctg123 . exon 5000 5500 . + . ID=exon00004;Parent=mRNA00001,mRNA00002,mRNA00003 11 ctg123 . exon 7000 9000 . + . ID=exon00005;Parent=mRNA00001,mRNA00002,mRNA00003 12 ctg123 . CDS 1201 1500 . + 0 ID=cds00001;Parent=mRNA00001;Name=edenprotein.1 13 ctg123 . CDS 3000 3902 . + 0 ID=cds00001;Parent=mRNA00001;Name=edenprotein.1 14 ctg123 . CDS 5000 5500 . + 0 ID=cds00001;Parent=mRNA00001;Name=edenprotein.1 15 ctg123 . CDS 7000 7600 . + 0 ID=cds00001;Parent=mRNA00001;Name=edenprotein.1 |
| **.bowtie** | text | Default output of BOWTIE alignments. See <http://bowtie-bio.sourceforge.net/manual.shtml>) | (1.1) Alignment; (1.2) De novo assembly; (2.1b) CNVs calling | FC30L0PAA_103008:5:1:914:86 - chr5 79982038 GAGTTCAGTTATATGTTTGGGATTTTT XXXXXYXXXXXXXXXXXXXXXXXXXXX 0 17:C>T  FC30L0PAA_103008:5:1:1163:464 - chr5 147683327 TCCTTCTTTATTCTTAGGAAGAGTAAA XXXXX\XXXXXXZXXXXXXXXXXXXXX 0  FC30L0PAA_103008:5:1:945:514 + chr4 1952734 AACTGTTAAACTAATGAGCAAGTAACA XXXWXXXXXXXXXXXWXXXXXWWXXXX 0  FC30L0PAA_103008:5:1:1515:1379 - chr3 97819124 TGTTACTTATACTAACATTAGTTCTTC XXXXXYXXXXY[XXXXXXXXXXXXXXX 1  FC30L0PAA_103008:5:1:1063:82 + chr7 26196374 GCCAATTTGATGTTACTGTTTTACAAA XXXXXXXXXXX[XXXXXXXXXXXXXUX 0  FC30L0PAA_103008:5:1:1320:499 - chr8 81713349 TCAAAAATATAACAAAATAATGCTACG UIDWXXXXXXXWXXXWXXXXXXWXXXX 0 24:C>A  FC30L0PAA_103008:5:1:1016:521 - chr3 97819124 TGTTACTTATACTAACATTAGTTCTTC XXXXXMXXXXXXWXXXXWXXXXXXXXX 1  FC30L0PAA_103008:5:1:1104:69 - chr20 25543647 CATTGTTAGGCTACACTGAGGAAATGT WXXXXXXXXXXXXYXXWXXXXXXXXXX 0  FC30L0PAA_103008:5:1:1379:1394 - chr3 97819097 AGGCTTATGCGGAGGAGAATGTTTTCA XXXWWXXXXXXXYXXXXXXXXXXXXXX 1 |
| **.contig** | text | File that contains the contigs assembled by the de novo assembly software | (1.1) Alignment; (1.2) De novo assembly | The format is variable |
| **.scaffold** | text | File that contains the scaffolds assembled by the de novo assembly software | (1.1) Alignment; (1.2) De novo assembly | The format is variable |
| **.hits** | text | File that contains the input reads for CNVseq | (2.1b) CNVs calling | 1 1234  1 23456  chr2 999  chr2 999  X 1234 |
| **.hits.cnv** | text | File that contains the CNV called by CNVseq. | (2.1b) CNVs calling |  |
| **.gsap** | text | SVA manages its projects through a project file. This project file is in a flat text format, and must have a file name extension *.gsap* . | (2.1a) Variant calling and annotation;(2.1b) CNVs calling |  |
| **.bco** | text | Coverage files needed by SVA. | (2.1a) Variant calling and annotation |  |
