# Supplementary material for: Next Generation Sequence Analysis and Computational Genomics Using Graphical Pipeline Workflows
Source: Genes (Basel). 2012 Aug 30;3(3):545–75. doi: 10.3390/genes3030545 (PMC3490498; doi:10.3390/genes3030545)
Supplement: Supplementary File 1 — ZIP-Document (ZIP, 7447 KB) [file genes-03-00545-s001.zip › Supplementary/TableS2.doc]

**GPCG module computational complexity and file size simulated reads**

| **Pipeline module/step** | **Est. computational complexity, runtime (trivial/complex, quick/long)** | | **Input data type** | | **Input data size** | | **Output data type** | | **Output data size** |
| --- | --- | --- | --- | --- | --- | --- | --- | --- | --- |
| **Ia. Sequence Alignment** | | | | | | | | | |
| **Pipeline name with sub-modules and time of run (minutes)** |  |  | |  | |  | |  | |
| **BWA (SE) ~132 mins** BWA:Index \| 68.56; BWA:aln \| 63.08; BWA:samse/sampe \| 1.34 | complex, long jobs | ASCII text (FASTQ) | | 2.4 Gb | | ASCII text (SAM) | | 1.6 Gb | |
| **BWA (PE) ~144 mins** BWA:Index 68.54; BWA:aln 68.18; BWA:samse/sampe 7.54 | complex, long jobs | ASCII text (FASTQ) | | 2.4 Gb x 2 | | ASCII text (SAM) | | 3.4 Gb | |
| **BWA (SW)** BWA:Index 80.26; bwasw 226.07 **~306 mins** | complex, long jobs | ASCII text (FASTQ) | | 2.4 Gb | | ASCII text (SAM) | | 3.0 Gb | |
| **SOAP(SE)** SOAP2bwt-builder 74.08; SOAP2 20.11; SOAPtoSAM 22.39 **~117 min** | complex, long jobs | ASCII text (FASTQ) | | 2.4 Gb | | ASCII text (SAM) | | 870Mb | |
| **SOAP(PE)** SOAP2bwt-builder 114.08; SOAP2 39; SOAPtoSAM 32 **~185 mins** | complex, long jobs | ASCII text (FASTQ) | | 2.4 Gb x 2 | | ASCII text (SAM) | | 3.2 Gb | |
| PERM 151.11 **PERM(SE) ~ 151 mins** | complex, long jobs | ASCII text (FASTQ) | | 2.4 Gb | | ASCII text (SAM) | | 1 Gb | |
| **PERM(PE) ~ 192 mins** PERM 192.28 | complex, long jobs | ASCII text (FASTQ) | | 2.4 Gb x 2 | | ASCII text (SAM) | | 3 Gb | |
| **BOWTIE (SE) (fgene3) ~ 177 mins** BOWTIE indexing 153.08; BOWTIE alignment 24.27 | complex, long jobs | ASCII text (FASTQ) | | 2.4 Gb | | ASCII text (SAM) | | 1 Gb | |
| **BOWTIE (PE)** BOWTIE indexing 155.08; BOWTIE alignment 50.25 **~ 205 mins** | complex, long jobs |  | | 2.4 Gb x 2 | | ASCII text (SAM) | | 2.8 Gb | |
| **MAQ (SE)** MAQ fasta2Bfa 1.11; MAQ mapper 780.48; SamTools maq2samlong 0.42 **~ 781 mins** | complex, long jobs |  | | 2.4 Gb | | ASCII text (SAM) | | 2.3 Gb | |
| **MAQ (PE)** MAQ fasta2Bfa 1.11; MAQ mapper 1183.10; SamTools maq2samlong 1.19 **~ 1184 mins** | complex, long jobs |  | | 2.4 Gb x 2 | | ASCII text (SAM) | | 4.8 Gb | |
| **MOSAIK (SE)** Mosaik Build 8; Mosaik Build 2.33; Mosaik Jump 4; Mosaik Aligner 1020; Mosaik Text 4 **~1138 mins** | complex, long jobs |  | | 2.4 Gb | | ASCII text (SAM) | | 1.1 Gb | |
| **MOSAIK (PE) ~1521 mins** Mosaik Build 15; Mosaik Build 2.33; Mosaik Jump 4.33; Mosaik Aligner 1500 | complex, long jobs |  | | 2.4 Gb x 2 | | ASCII text (SAM) | | 2.9 Gb | |
| **Ib. De novo assembly (tested on 150k reads subset)** | | | | | | | | | |
| **SOAPdeNOVO (SE) (~1 min)** SOAP config; SOAP de novo | Quick (because of the reduced size of the simulated data) | |  | | 2.4 Gb | | ASCII text (contigs, scaffolds) | | variable |
| **SOAPdeNOVO (PE) (~2 mins)** SOAP config; SOAP de novo | Quick (because of the reduced size of the simulated data) | |  | | 2.4 Gb x 2 | | ASCII text (contigs, scaffolds) | | variable |
| **VELVET (SE)** fastx: Fastq2fasta; Velveth; Velvetg **(~1 min)** | Quick (because of the reduced size of the simulated data) | |  | | 2.4 Gb | | ASCII text (contigs) | | variable |
| **VELVET(PE)** fastx: Fastq2fasta; Velveth; Velvetg **(~2 mins)** | Quick (because of the reduced size of the simulated data) | |  | | 2.4 Gb x 2 | | ASCII text (contigs) | | variable |
| **ABYSS (SE)** fastx: Fastq2fasta; Abyss-pe **(~1 min)** | Quick (because of the reduced size of the simulated data) | |  | | 2.4 Gb | | ASCII text (contigs) | | variable |
| **ABYSS (PE)** fastx: Fastq2fasta; Abyss-pe **(~2 mins)** | Quick (because of the reduced size of the simulated data) | |  | | 2.4 Gb x 2 | | ASCII text (contigs) | | variable |
| **II. Post-alignment processing: Basic QC (23 minutes)** | | | | | | | | | |
| **Sub-modules and time of run (minutes)** |  | |  | |  | |  | |  |
| Indexing of the reference genome 1 | trivial, quick jobs | | ASCII text  (fasta) | | large (~3 Gb) | | index (fai) | | small (few MBs) |
| Clean SAM 2 | " | | ASCII text (SAM) | | large (3.4 Gb)* | | ASCII text (SAM) | | large (~3.2 Gb)* |
| SAM to BAM conversion 3 | " | | ASCII text (SAM) | | large (3.2 Gb) | | BINARY data (BAM) | | large (~2 Gb) |
| Sort BAM file 3.5 | " | | BINARY data (BAM) | | large (~2 Gb) | | BINARY data (BAM) | | large (~2 Gb) |
| Remove duplicates 2 | “ | | BINARY data (BAM) | | large (2 Gb) | | BINARY data (BAM) | | large (~2 Gb) |
| Fix mates 5.4 | " | | “ | | “ | | “ | | “ |
| SamTools: CamId (MD tag) 3 | “ | | “ | | “ | | “ | | “ |
| index BAM file 0.5 | " | | " | | " | | ‘ | | small (few Mb) |
| Validate Sam 1 | " | | " | | " | | BINARY  (BAM) | | large (~2 Gb) |
| **III. Advanced QC: ~ 565 mins** | | | | | | | | | |
| **Base quality recalibration, local realignment** **Sub-modules and time of run (minutes)**; Interval Creation 141; Realigning 90; Sort Sam 5; Mark duplicates 3; PICARD Index 2; GATK covariates calculation 103; Fix Mate information 4 min; Validate Sam 3 min; GATK covariates analysis 60; PICARD Index 3; GATK covariates calculation 95; GATK covariates analysis 70 **(~550 mins)** | complex, long jobs | | BINARY data (BAM) | | large (2 Gb) | | BINARY data (recalibrated, re-aligned BAM) | | large (2 Gb) |
| **QC stats, plots, graphs** **Sub-modules and time of run (minutes)**; Quality Score Distribution 3; Collect Insert size metrics 2; Mean Quality by cycle 2; Collect Alignment Summary Metrics 2; Collect GC bias metrics 3 min; GATK callability 2; Indexing 2 **(~15 mins)** | quick jobs | | BINARY data (BAM) | | large (2 Gb) | | PDF/text charts and tables | | small (few Mb) |
| **IV. Variant Calling, Annotation, Visualization** | | | | | | | | | |
| **Sub-modules and time of run (minutes)** |  | |  | |  | |  | |  |
| **SVA 1.0 ~ 1 hour 30 minutes** |  | |  | |  | |  | |  |
| PILEUP ~20 minutes | complex, long jobs (over 24 hours on large BAMs) | | BINARY data (BAM) | | large (2 Gb) | | ASCII text (PILEUP) | | very large (5 Gb) |
| VARIANTS ~10 minutes | trivial, quick job | | ASCII text (PILEUP) | | very large (5 Gb) | | ASCII text | | small (few Mb, .snpindel) |
| SNV ~10 minutes | trivial, quick job | | " | | " | | " | | small (few Mb, samtools) |
| INDELS ~10 minutes | trivial, quick job | | " | | " | | " | | small (few Mb, samtoolindels) |
| BCO ~10 minutes | trivial, quick job | | " | | " | | “ | | small (few Mb, .bco) |
| ERDS-CNVs ~10 minutes | trivial, quick | | “ | | “ | | “ | | small (few Mb, .events) |
| Create GSAP file | trivial, quick job | | Various ASCII text files (.snpindel, .samtools, .samtoolsindels, .bco, .events) | | large (2 Gb) | | ASCII text (GSAP) | | small (few Kbs) |
| SVA annotate 20 minutes | trivial, quick job | | multiple data files | | small (few Kbs) | | ASCII text (GSAP) | | small (few Kbs) |
| **Sub-modules and time of run (minutes)** |  | |  | |  | |  | |  |
| **SAMTOOLS-ANNOVAR** SamTools: Bam to VCF 25; Convert2annovar 2.5; Annotate Variation 2.5 **~30 mins** | trivial, quick job (~3-4 hours on the entire genome) | | BINARY data (BAM) | | large (2 Gb) | | ASCII text | | Medium, depending on how many annotation field required (Mb) |
| **UNIFIED GENOTYPER-ANNOVAR ~ 2 hours** GATK unified genotyper 2 hours; Convert2annovar 2.5 min; Annotate Variation 2.5 min | trivial, quick job (~3-4 hours on the entire genome) | | BINARY data (BAM) | | large (2 Gb) | | ASCII text | | Medium, depending on how many annotation field required (Mb) |
| **V. CNV calling** | | | | | | | | | |
| **Sub-modules and time of run (minutes)** |  | |  | |  | |  | |  |
| **ERDS** ~10 minutes | trivial, quick job (~3-4 hours on an entire genome) | | ASCII text (PILEUP) | | Very large (5 Gb) | | ASCII text | | small (few Mb, .snpindel) |
| **CNVer** Bowtie-build 0.2; Bowtie 861; Sam Format Converter 222; Samtools: Sort 312; Samtools: Index 0.2; CNVer 60.10 **~1380 mins** | complex, long jobs (over 24 hours on large BAMs) | | BINARY data (BAM) | | large (2 Gb) | | ASCII text | | Medium (Mb) |
| **CNVseq** Extract hits 33.18; Extract hits 19.51; CNV-Seq 352.35; CNV-R 24 **~210 mins** | trivial, quick job (~6-7 hours on an entire genome) | | BINARY data (BAM) | | large (2 Gb) | | ASCII text | | Small (few Mb) |

| **Genomics pipeline module computational complexity and file size real human high coverage data** | | | | | |
| --- | --- | --- | --- | --- | --- |
|  | | | | | |
| **Pipeline module/step and time of run (m)** | **Est. computational complexity, runtime (trivial/complex, quick/long)** | **Input data type** | **Est. input data size** | **Output data type** | **Est. output data size** |
| **I. Sequence Alignment** | | | | | |
| BWA (PE) 1 lane  (360 mins) | complex, long jobs | ASCII text (FASTQ) | Very large (~40 Gb) | ASCII text (SAM) | Very large (~70 Gb) |
|  | 1 lane 2x100 WGS input (240-320 mins to completion on 12 CPU core, 24GB RAM node) |  |  |  |  |
|  |  |  |  |  |  |
| BWA (PE) (all the flowcell, 2880 mins) | complex, long jobs | ASCII text (FASTQ) | Very large (~320 Gb) | ASCII text (SAM) | Very Large (~560 Gb) |
|  |  |  | ~40 GB per lane |  | ~ 70 Gb per lane |
|  |  |  |  |  |  |

**GPCG module computational complexity and file size real reads**
